# Pequi (*Caryocar brasiliense*, Camb) Bark Extract Reduces ROS Production in Diabetic Human Coronary Endothelial Cells

**DOI:** 10.3390/antiox14101167

**Published:** 2025-09-25

**Authors:** Karla M. S. Braga, Eugenio G. Araujo, Frank W. Sellke, M. Ruhul Abid

**Affiliations:** 1 Cardiovascular Research Center, Division of Cardiothoracic Surgery, Department of Surgery, Rhode Island Hospital, Warren Alpert Medical School, Brown University, Providence, RI 02903, USA; karla_da_silva_braga@yahoo.com (K.M.S.B.); earaujo@ufg.br (E.G.A.); frank_sellke@brown.edu (F.W.S.); 2 School of Veterinary Medicine, Federal University of Goias, Goiania 74690-900, Brazil

**Keywords:** alternative medicine, coronary artery endothelium, endogenous antioxidant enzymes, hypoxia, natural products, type 2 diabetes

## Abstract

Reactive oxygen species (ROS) overproduction contributes to endothelial dysfunction in Type 2 diabetes mellitus (T2DM). Pequi (*Caryocar brasiliense*, Camb), a native Brazilian fruit, is rich in polyphenolic antioxidants. We investigated whether its ethanolic bark extract modulates ROS levels and promotes proliferation in human coronary artery endothelial cells from patients with diabetes (D-HCAECs). Cells were treated with pequi extract under normoxic, hypoxic, or H_2_O_2_-induced oxidative stress conditions. Cytosolic and mitochondrial ROS levels, cell proliferation, and the expression of antioxidant proteins (Nrf2, HO-1, SOD1, SOD2, catalase, and GPx1) were assessed. Pequi significantly reduced cytosolic ROS under normoxia and both cytosolic and mitochondrial ROS under stress. It also upregulated antioxidant enzymes through the Nrf2 pathway and enhanced D-HCAEC proliferation under all tested conditions. These results suggest that pequi’s antioxidant effects may be mediated by the increased expression of endogenous enzymes, leading to improved redox balance and endothelial function in diabetic coronary vasculature.

## 1. Introduction

Optimal coronary vascular function and related vasomotor activity are primarily dependent on endothelial function, which is often affected by factors such as diabetes, hypertension, hyperlipidemia, and obesity [1]. Diabetes (DM) is one of the most common chronic non-communicable diseases with significant cardiovascular morbidity and mortality. DM was also associated with vascular complications and increased mortality during the coronavirus disease 2019 (COVID-19) pandemic [2].

Type 2 diabetes mellitus (T2DM) corresponds to 90–95% of all cases of diabetes [3]. The main features are a systemic inflammatory condition accompanied by hyperglycemia and insulin resistance (IR) or a decreased metabolic response to insulin in tissues such as skeletal muscle, adipose tissue, and the liver, with reduced insulin synthesis by pancreatic beta cells. As the disease progresses, hyperglycemia promotes mitochondrial dysfunction and induces increased levels of reactive oxygen species (ROS) known as “oxidative stress” in several tissues, including endothelial cells [4].

In T2DM, coronary endothelial cells (ECs) have been reported to exhibit decreased endothelial nitric oxide synthase (eNOS) levels, increased oxidant levels, and inflammatory markers leading to coronary vascular pathologies, including coronary microvascular dysfunction [5].

The endogenous antioxidant system maintains cellular oxidative balance, preventing the accumulation of oxidants. This crucial balance may be perturbed due to disease or altered metabolic conditions. ROS are essential mediators of oxidative stress and its complications. The outcome may be more devastating for patients with diabetes since the excess ROS production is coupled with diminished endogenous antioxidant function and increased systemic inflammation. Hyperglycemia, ROS accumulation, and the inflammatory response are critical factors in initiating and progressing vascular endothelial cell (EC) damage, leading to microvascular dysfunction. Microvascular dysfunction in T2DM may lead to cardiovascular disease (CVD), including myocardial ischemia, myocardial infarction, peripheral arterial disease, chronic kidney disease, and retinopathy [6].

Research by several groups has shown that naturally occurring compounds can improve vascular EC function. The addition of polyphenolic compounds has modulated several cellular signaling pathways, thereby promoting vascular homeostasis [7]. Additionally, strategies involving the dietary intake of polyphenolic compounds, such as the Mediterranean diet, have been reported to be associated with a reduced incidence of CVD in obese individuals [8]. Furthermore, polyphenols and natural compounds, such as curcumin, resveratrol, naringenin, cinnamon, capsaicin, berberine, and genistein, among others, have also been shown to be effective in managing T2DM and its complications [9].

Pequi (*Caryocar brasiliense*, Camb) is a typical edible fruit from the Brazilian savanna-like biome “Cerrado”, widely used in popular medicine. Phenolic compounds are abundant in the extract obtained from the bark (mesocarp), the pulp, and the nut oil of pequi [10]. In addition to the benefits reported, the extract was shown to have very low toxicity potential [11,12].

Increasing attention has been given to nutraceuticals—bioactive compounds derived from foods that provide health benefits beyond basic nutrition. Nutraceuticals, such as polyphenols, flavonoids, and plant-derived antioxidants, have been widely investigated for their role in reducing oxidative stress, modulating inflammatory pathways, and improving endothelial function in metabolic and cardiovascular disorders [13]. Within this context, pequi may be considered a promising nutraceutical source as its bark extract is rich in polyphenolic compounds.

For over two decades, our group has been studying oxidative stress in microvascular function in health and disease [1,14,15,16] and has even proposed new therapeutic approaches, such as using human bone–mesenchymal stem-cell-derived extracellular vesicles to improve damaged endothelium [17]. Accordingly, the current study aimed to investigate whether the ethanolic extract of pequi bark modulates reactive oxygen species (ROS) levels and enhances cellular proliferation in diabetic human coronary artery endothelial cells (D-HCAECs).

## 2. Materials and Methods

### 2.1. Pequi Extract Preparation

Pequi bark samples (and mesocarps) were harvested in Central Brazil (15.032232″ S and 49.942103″ W at 730.5 m altitude) and prepared as previously reported [18]. In brief, after the bark samples were crushed into a powder and dried, the pulverized material was subjected to a cold maceration process using 95% (*w*/*v*) ethanol as the extracting liquid (1:3). After maceration, filtration, and subsequent concentration in a rotary evaporator at 40 °C, we stored the final ethanol-free product (ethanolic extract of pequi bark) at −20 °C, protected from light.

The extract was subjected to High-Performance Liquid Chromatography coupled with High-Resolution Mass Spectrometry Analysis. The main compounds observed were gallic acid, protocatechuic acid, as well as quercetin and catechin (Table 1). Other compounds present in the extract were gentisic, caffeic, p-coumaric, vanillic, and ellagic acids, as well as picatechin, rutin, naringenin, luteolin, and kaempferol. (See Supplementary Materials).

### 2.2. Cell Culture Procedure

D-HCAECs Type II (Cat. #CC-2922) were purchased from Lonza (Walkersville, MD, USA). After thawing, D-HCAECs were transferred to 100 × 20 mm tissue culture dishes with Endothelial Cell Basal Medium-2 (EBM-2, Cat. #3156, Thermo Fisher Scientific, Waltham, MA, USA), supplemented with Microvascular Endothelial Cell Medium-2 EGM^®^-2 MV Single Quotes^®^ Kit (Cat. # CC-4147) and 5% fetal bovine serum (Lonza, Basel, Switzerland). Cells were maintained in a humidified atmosphere (90 ± 2%) at 37 ± 1 °C, with 5% CO_2_. Fresh media was added every other day and trypsinized according to the supplier’s protocols. Passages of 3–5 cells were used for further experiments.

### 2.3. Experimental Groups and Proliferation Assay in D-HCAECs

When confluent, D-HCAECs were trypsinized to form a cell suspension normalized to 6 × 10^4^ cells/mL, transferred to a 96-well plate, and pre-incubated with extract at 10 μg/mL or without extract (vehicle control) for 24 h, resulting in five groups of six repetitions each. Except for controls, we added 100 μM H_2_O_2_ to the cells for six hours. Nuclear stain DAPI was applied to each well at a concentration of 1 μg/mL and discarded after 15 min. Cells were observed using a fluorescence microscope, and scanned images were analyzed with ImageJ version 1.54p software.

### 2.4. Stress Induction in D-HCAECs

Pequi extract was added to the media at 10 or 25 μg/mL, either before establishing hypoxia (Hypoxia Protocol) or 48 h before adding H_2_O_2_, except in negative controls (DMSO only, extract vehicle).

We simulated hypoxia by removing oxygen from a Modular Incubator Chamber (Hypoxia Chamber, MIC-101, Billups-Rothenberg, San Diego, CA, USA) using a 95% N_2_ and 5% CO_2_ gas mixture at a flow rate of 20 L/min. A one-way valve was placed in each hole, allowing gas from the tank to purge the hypoxic gas. Petri dishes were placed in the hypoxia chambers containing sterile water to provide adequate humidification for the cultures. Two identical 96-well cell culture plates (twin culture) were prepared, one of which was placed in the hypoxic chamber, and the other was maintained in normoxia as a control. After seven minutes, the gas flow was shut down, and the chamber was sealed by closing the clamps to maintain a 1% oxygen level in the hypoxic chamber. Subsequently, the sealed chamber was placed in a conventional incubator for 48 h at 37 °C. An alternative oxidative stress condition was induced by adding 100 μM H_2_O_2_ to the media of D-HCAECs for six hours.

Pequi extract was added to the media of the experimental group of D-HCAECs at concentrations of 10 or 25 μg/mL, based on previous work with the extract [18]. The pequi extract vehicle (DMSO only) was added to the D-HCAECs as a negative control. Since the experiments were conducted separately, a DMSO negative control was used for each pequi extract concentration (10 or 25 μg/mL).

### 2.5. Determination of Cytosolic ROS Production in D-HCAECs

Following D-HCAEC pre-treatment with different pequi extract concentrations and the induction of hypoxia (1% oxygen) or oxidative stress (100 μM H_2_O_2_), 25 μM 2,7-dichlorodihydrofluorescein diacetate (H2DCF-DA) fluorescent probe (Sigma-Aldrich, St. Louis, MO, USA) was added. D-HCAECs were maintained at 37 °C, in the dark, for an additional 30 min. Upon oxidation, the nonfluorescent H2DCF-DA turns into the fluorescent 2′,7′-dichlorofluorescein (DCF). The fluorescence intensity, at excitation and emission wavelengths of 485 nm and 528 nm, respectively, was measured using a microplate reader. The optical densities were normalized by subtracting the respective background values from wells without DCF reagent.

### 2.6. Mitochondrial ROS Measurement in D-HCAECs

D-HCAECs (1 × 10^4^ cells/well) were grown as previously described [17]. MitoSox^TM^ Red reagent (Invitrogen, Carlsbad, CA, USA) solution was freshly prepared by adding dimethyl sulfoxide (DMSO) to a MitoSox vial (500 µM). Modified HBSS (Hank’s Balanced Salt Solution, JRH Biosciences, Lenexa, KS, USA) was mixed with MitoSox reagent according to the manufacturer’s protocol. A total of 100 µL of the mix was added to each well with D-HCAECs and incubated at 37 °C for 15 min. The absorbance was measured at an excitation wavelength of 510 nm and an emission wavelength of 580 nm. Optical densities were normalized by subtracting the respective background values from wells without the MitoSox reagent.

### 2.7. Western Blotting

Cells were grown to 80–90% confluence. Two groups were treated with 25 μg/mL of pequi extract for 48 h, and then H_2_O_2_ was added to one of the groups. Two pequi extract-negative control groups (one containing DMSO only and the other containing vehicle only) were established, with one treated with H_2_O_2_ and the other left untreated. Cell lysates were prepared, and Western Blot (WB) analyses were performed as previously described [14]. We used primary antibodies against Nrf2 (#12721), HO-1 (#26416), SOD1 (catalogue #37385T), SOD2 (#13141S), Catalase (#12980S), and GPX-1 (#3206S). All antibodies were purchased from Cell Signaling (Danvers, MA, USA).

### 2.8. Statistical Analysis

All experiments were performed with a minimum of three independent biological replicates, and technical replicates were included within each assay. Data were first inspected for completeness and potential outliers using descriptive statistics and graphical exploration (boxplots and residual plots). Normality of data distributions was evaluated using the Shapiro–Wilk test, and homogeneity of variances was assessed using Levene’s test.

For comparisons between two groups (e.g., treatment vs. control under the same condition), Welch’s *t*-test was applied because it does not assume equal variances between groups and is, therefore, more robust for small sample sizes or unequal variances. For experiments involving more than two groups (e.g., multiple concentrations of pequi extract across normoxia, hypoxia, or H_2_O_2_-induced stress), a one-way analysis of variance (ANOVA) was performed, followed by Tukey’s post hoc test to adjust for multiple comparisons. When two independent factors were involved (e.g., treatment concentration × stress condition), a two-way ANOVA was employed to evaluate main effects and interactions.

Results from proliferation and ROS assays were converted to arbitrary units, with control means set at 100%, to facilitate comparison across experiments. Data from Western blot densitometry were normalized to GAPDH expression and compared to respective controls using Welch’s *t*-test.

All statistical analyses were conducted in Prism software (GraphPad, Version 10.0, San Diego, CA, USA). Results are presented as mean ± standard deviation (SD). A two-tailed significance level of *p* < 0.05 was considered statistically significant. Exact *p*-values are reported where appropriate, and non-significant results are indicated as “NS”.

## 3. Results

### 3.1. Pequi Extract Enhances Proliferation in D-HCAECs Under Stress Conditions

**Figure 1:**
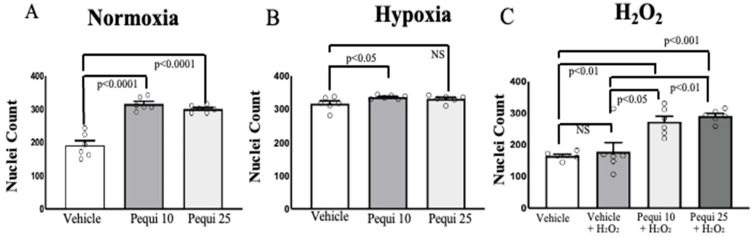
Pequi increases diabetic human coronary artery endothelial cell proliferation. D-HCAECs were pre-treated for 24 h with 0 (vehicle), 10, or 25 μg/mL of pequi extract. Cells were subject to normoxia (**A**) or hypoxia (**B**) for 48 h or to 100 μM H_2_O_2_ for six hours (**C**). Under hypoxic conditions, cell proliferation significantly increased in D-HCAECs treated with 10 μg/mL compared to the vehicle-only (DMSO) control (**B**). D-HCAECs incubated with 100 μM H_2_O_2_ showed increased proliferation when cells were pre-treated with pequi extract (10 or 25 μg/mL) (**C**). Statistical analysis was conducted using Welch’s *t*-test. Variance analysis was performed using ANOVA. Values with *p* > 0.05 were considered non-significant. Bars indicate SEM, and blank dots represent arbitrary unit values.

The addition of pequi extract (10 and 25 μg/mL) under normoxic conditions significantly increased D-HCAEC proliferation as compared to the vehicle (DMSO) control (Figure 1A). Under hypoxia, D-HCAEC proliferation increased significantly (by 6.2 ± 2.15%; *p* < 0.05) when treated with 10 μg/mL of pequi extract, although not with 25 μg/mL (*p* = 0.1255) (Figure 1B). On the other hand, when we added H_2_O_2_ (100 μM) to the media for six hours, the addition of 10 μg/mL and 25 μg/mL of pequi extract increased proliferation by 54 ± 19% (*p* < 0.05) and 63.7 ± 16.85% (*p* < 0.01), respectively, as compared to vehicle control with H_2_O_2_ (Figure 1C). Variance analysis demonstrated significant changes among vehicle-only, vehicle with H_2_O_2_, versus H_2_O_2_ groups treated with pequi extract, in both 10 μg/mL (*p* = 0.003) and 25 μg/mL (*p* = 0.0002) concentrations (Figure 1C).

### 3.2. Treatment with Pequi Extract Reduces Cytosolic ROS Levels in D-HCAECs

**Figure 2:**
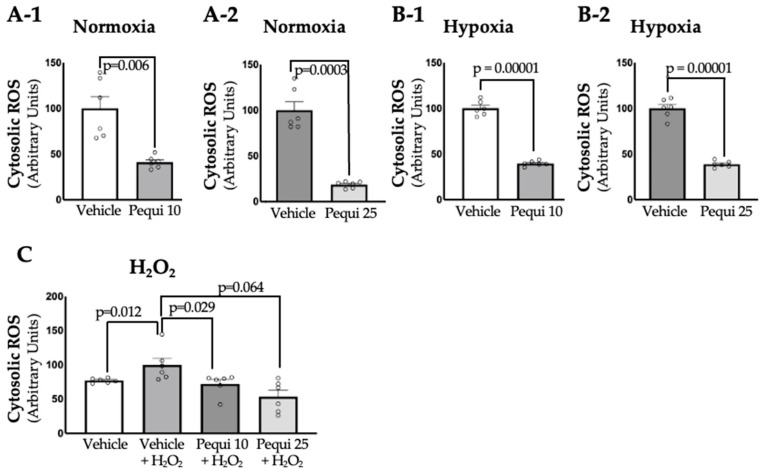
Pequi reduces cytosolic ROS in diabetic human coronary artery endothelial cells (D-HCAECs). Cells were pre-treated with 0 (control), 10, or 25 μg/mL of pequi extract for 24 h, followed by normoxia (**A-1**,**A-2**), 48 h of hypoxia (**B-1**,**B-2**), or hyperoxia via 6-h exposure to 100 μM H_2_O_2_ (**C**). Pequi extract decreased ROS levels in all treatment groups compared to the control group. Notably, the decrease in ROS levels was more significant at the 25 μg/mL dose in both conditions of oxidative stress, hypoxia (**B-2**), and H_2_O_2_ (**C**). The extract vehicle (DMSO) served as the negative control for all pequi extract concentrations tested (10 or 25 μg/mL). For Welch’s *t*-test, values with *p* > 0.05 were considered non-significant. Bars indicate SEM, and blank dots represent arbitrary unit values.

Upon treatment with pequi extract, cytosolic ROS levels in D-HCAECs were measured in both control (vehicle) groups and pequi extract-treated groups under three conditions: normoxia, hypoxia (48 h), and oxidative stress, i.e., treatment with H_2_O_2_ for six hours (Figure 1). Interestingly, ROS reduction by pequi extract was observed in D-HCAECs that were maintained in normoxia for 48 h (Figure 2(A-1,A-2)); in ECs subjected to hypoxia for 48 h (Figure 2B); as well as in ECs treated with H_2_O_2_ for 6 h (Figure 2C). Although both extract concentrations (10 and 25 μg/mL) reduced ROS production, the effect was more pronounced in the 25 μg/mL pequi group, see Figure 2(A-2,B-2). Together, these results suggest that pequi extract can significantly reduce cytosolic ROS levels in D-HCAECs under normoxic and stress (both hypoxic and oxidative stress) conditions.

### 3.3. Pequi Extract Also Reduces Mitochondrial ROS Levels in D-HCAECs

**Figure 3:**
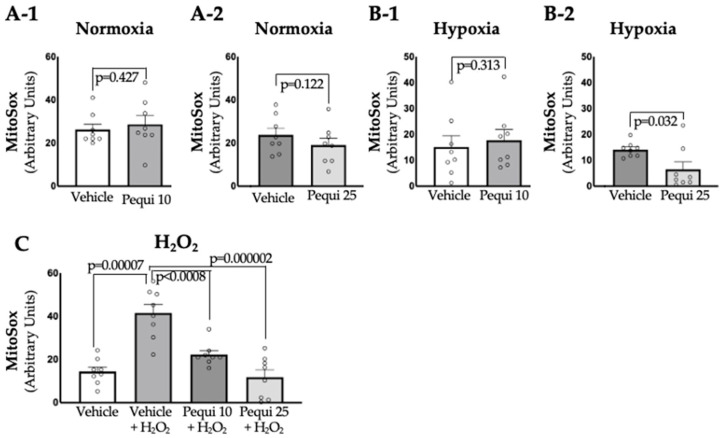
Pequi reduces mitochondrial ROS in diabetic human coronary artery endothelial cells (D-HCAECs). Cells were pre-treated with 0 (control), 10, or 25 μg/mL of pequi extract for 24 h, followed by normoxia (**A-1**,**A-2**), 48 h of hypoxia (**B-1**,**B-2**), or a 6-hour exposure to 100 μM H_2_O_2_ (**C**). Pequi extract decreased mitochondrial ROS production in the hypoxia group pre-treated with 25 μg/mL (**B-2**). Although adding H_2_O_2_ increased mitochondrial ROS levels in D-HCAECs not treated with the extract, adding pequi (10 or 25 μg/mL) reduced ROS levels (**C**). The extract vehicle (DMSO) served as the negative control for all pequi extract concentrations tested (10 or 25 μg/mL). For Welch’s *t*-test, values with *p* > 0.05 were considered non-significant. Bars indicate SEM, and blank dots represent arbitrary unit values.

Like cytosolic ROS, pequi extract significantly reduced mitochondrial (mito)-ROS in D-HCAECs under hypoxia and H_2_O_2_ treatment conditions (Figure 3(B-2,C)). Interestingly, there were no changes in mito-ROS in D-HCAECs treated with pequi extract (10 μg/mL and 25 μg/mL) and incubated under normoxic conditions (Figure 3(A-1,A-2)), or when treated with pequi at 10 μg/mL under hypoxic conditions (Figure 3(B-1)). For the groups treated with pequi extract at 25 μg/mL, mitochondrial ROS levels were reduced by 64.29 ± 6% in hypoxia (Figure 2B) and by 74.7 ± 12.53% in D-HCAECs treated with H_2_O_2_ (Figure 3C) but not in D-HCAECs under normoxia (Figure 3(A-2)). Together, these findings suggest that under stress conditions, including hypoxia and oxidative stress (H_2_O_2_), pequi extract at a concentration of 25 μg/mL is effective in significantly reducing mitochondrial reactive oxygen species (mito-ROS).

### 3.4. Pequi Extract-Induced Increased Expression of Antioxidant Enzymes Is Associated with the Nrf2 Pathway in D-HCAECs

**Figure 4:**
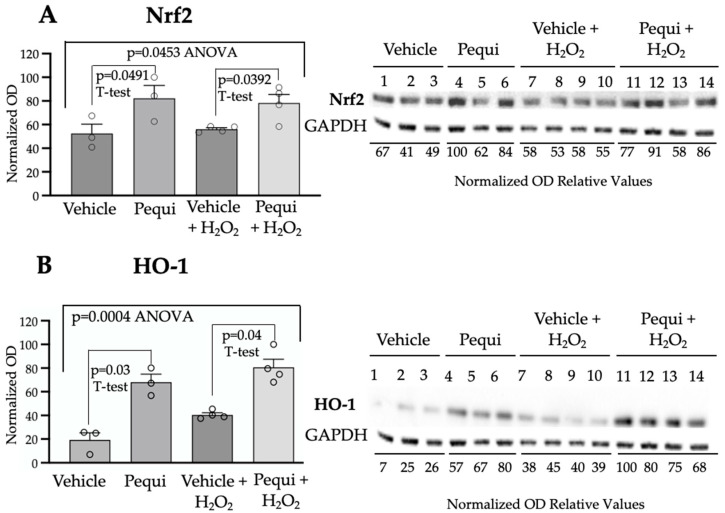
Pequi extract increases expression of Nrf2 and HO-1. Cells were cultured and pre-treated with pequi extract (25 μg/mL) or respective control (extract vehicle DMSO) for 48 h under normoxic conditions, with or without the addition of 100 μM H_2_O_2_. Total protein content was extracted for Western blot analysis of Nrf2 (**A**) and HO-1 (**B**). Western blots were normalized using GAPDH as a loading control. Statistical analysis was performed between the pequi and respective control groups using Welch’s *t*-test (*p* < 0.05), and ANOVA (*p* < 0.05) was used to compare all groups. Bars indicate SEM, and blank dots in the graphs represent the normalized optical density (OD) relative values obtained for each sample.

**Figure 5:**
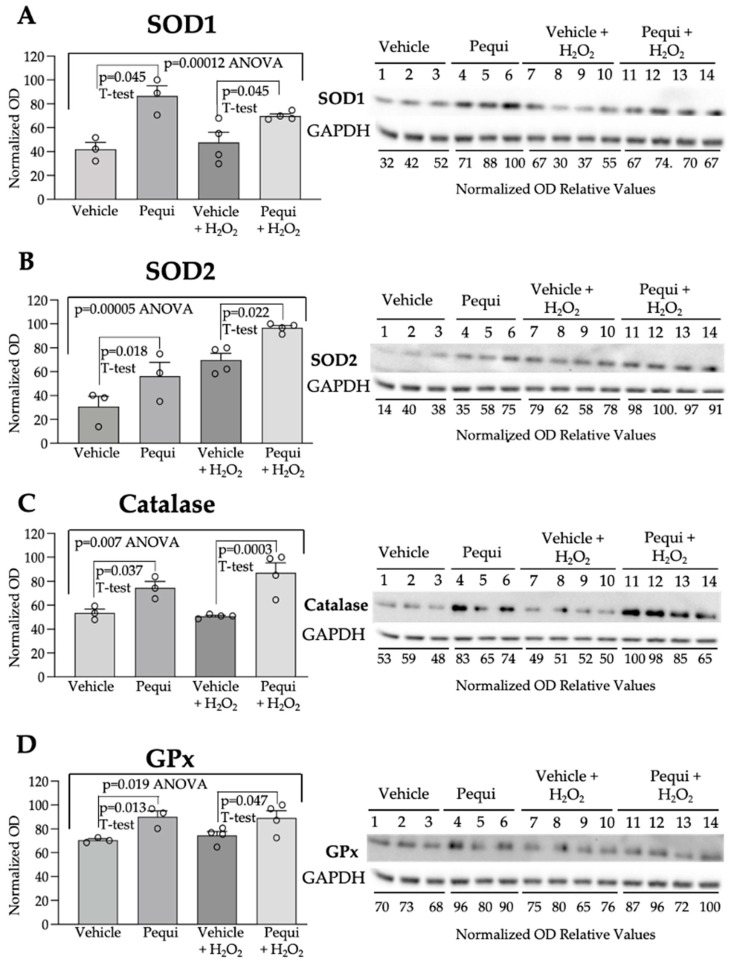
Pequi extract increases expression of SOD1, SOD2, catalase, and glutathione peroxidase. Cells were cultured and pre-treated with pequi extract (25 μg/mL) for 48 h under normoxic conditions, or respective control (extract vehicle DMSO) for 48 h under normoxic conditions, with or without the addition of 100 μM H_2_O_2_. Total protein content was extracted for Western blot analysis of SOD1 (**A**), SOD2 (**B**), catalase (**C**), and glutathione peroxidase (GPx) (**D**). Western blots were normalized using GAPDH as a loading control. Statistical analysis was performed between the pequi and respective control groups using Welch’s *t*-test (*p* < 0.05), and ANOVA (*p* < 0.05) was used to compare all groups. Bars indicate SEM, and blank dots in the graphs represent the normalized optical density (OD) relative values obtained for each sample.

Since pequi extract decreased ROS levels, we next examined whether pequi affects Nrf2 expression and other endogenous antioxidant defense mechanisms in D-HCAECs using Western blot (WB) analysis. Cells were treated with 25 μg/mL of pequi extract or control (DMSO, extract vehicle) for 48 h under normoxic conditions, with or without the induction of oxidative stress by the addition of 100 μM H_2_O_2_, and then subjected to WB (Figure 4 and Figure 5).

The expression of antioxidant transcription factor Nrf2 was significantly increased (*p* = 0.0491) in the pequi extract-treated D-HCAECs compared to vehicle-treated control cells (Figure 4A). In parallel, D-HCAECs were treated with 100 μM H_2_O_2_ to induce oxidative stress. Pequi also increased Nrf2 expression (*p* = 0.0392) in the 100 μM H_2_O_2_-treated D-HCEACs (Figure 4A). As expected, a similar effect was observed in HO-1 expression (Figure 4B).

Furthermore, major endogenous antioxidant enzymes were significantly activated by pequi extract (Figure 5). SOD1 expression (Figure 5A) was increased by adding extract alone (*p* = 0.045), and also when D-HCAECs were treated with 100 μM H_2_O_2_ (*p* = 0.045). A similar trend was observed for SOD2 (Figure 5B, *p* = 0.018 and 0.022, respectively), catalase (CAT, Figure 5C, *p* = 0.037 and 0.003, respectively), and GPx (Figure 5D, *p* = 0.013 and 0.047, respectively).

## 4. Discussion

The current study demonstrates that pequi extract decreases cytosolic ROS levels and increases proliferation in D-HCAECs subjected to normoxia, hypoxia, and oxidative stress conditions. Increased levels of ROS are often associated with microvascular pathology in ischemic heart disease, causing endothelial dysfunction and coronary artery disease, leading to myocardial ischemia and infarction [19]. Since therapeutic interventions are frequently unsuccessful [20], even in recent approaches such as the use of mesenchymal stem-cell-derived extracellular vesicles [21], preventive measures—including dietary supplements [22]—have been widely proposed. Among those supplements stand polyphenols, molecules found in various plants worldwide—such as the Brazilian Pequi (*C. brasiliense*)—which are implicated in reducing oxidative stress [23].

The effects of pequi extract, as shown in this study, had a substantial impact, considering the poor response of diabetic endothelial cells to stress [7]. Since HCAECs in diabetes are more apoptotic due partly to mitochondrial calcium overload, coronary microvascular rarefaction due to endothelial cell dysfunction causes increased morbidity and mortality in this disease [7]. Insulin resistance and hyperinsulinemia impair microvascular endothelium-dependent coronary vasodilatation in prediabetes through increased oxidative stress, inflammation, and dyslipidemia [5].

Cytosolic ROS levels decreased in D-HCAECs under normoxic conditions (Figure 1) after the addition of pequi extract, and the same group of cells exhibited significant proliferation when treated with pequi (Figure 3). Since the cells were not subjected to stress conditions, such as hypoxia or H_2_O_2_ addition to the media, these results could be unexpected if the cells had not been obtained from patients with diabetes. However, diabetes constitutes, in itself, a stress factor to the endothelial cell [24].

ROS levels decrease in D-HCAECs, confirming that the pequi extract has antioxidant properties. Indeed, qualitative High-Pressure Liquid Chromatography coupled with High-Resolution Mass Spectrometry (HPLC-HRMS) analysis showed that the pequi extract that we prepared contains gallic, protocatechuic, gentisic, caffeic, p-coumaric, vanillic, and ellagic acids, as well as catechin, epicatechin, rutin, quercetin, naringenin, luteolin, and kaempferol [18]. Other studies have explored the molecular mechanisms underlying the antioxidant capacity of polyphenols, such as those found in pequi, which is attributed to the regulation of redox enzymes by reducing ROS production and the modulation of phase II enzymes responsible for the cellular oxidative response [13,23].

Based on the observed decrease in ROS levels in D-HCAECs following pequi treatment, our finding that pequi activates the Nrf2/HO-1 pathway in diabetic endothelial cells is highly significant as it supports the hypothesis that pequi reduces ROS by activating antioxidant enzymes (Figure 4 and Figure 5). The KEAP1/NRF2/antioxidant response element pathway is one of the most effective intracellular antioxidant stress pathways, playing a crucial role in diabetes-induced endothelial dysfunction [25].

Our pequi extract is rich in phenolic compounds [18]. As verified here, the activation of the Nrf2 pathway is believed to be a beneficial effect of polyphenols in diabetic cells. For instance, the polyphenolic Sinapic acid induced Nrf2 and HO-1 mRNA expression levels in the myocardia of streptozocin-induced diabetic rats. It ameliorated cardiac dysfunction and cardiomyopathy by improving hyperglycemia, hyperlipidemia, inflammation, oxidative stress, and apoptosis [26]. A similar effect was observed in streptozotocin-induced type 2 diabetic mice subjected to myocardial ischemia and reperfusion, where the well-known phenol resveratrol restored the expression of Nrf2 and HO-1 in the heart and reduced infarct size [27].

Kaempferol is one of the components of the pequi extract used in this investigation. Others have reported that kaempferol significantly activated the Nrf2/HO-1 signaling pathway in human umbilical vein endothelial cells (HUVECs) subjected to 100 mmol/L of H_2_O_2_ compared to H_2_O_2_ alone—a protocol identical to the one we employed. Those findings were consistent with the role of kaempferol in reducing oxidative stress through the Nrf2/HO-1 pathway [28]. Furthermore, the administration of kaempferol to streptozotocin-diabetic rats significantly preserved the systolic and diastolic functions of the left ventricle (LV), lowered reactive oxygen species (ROS) levels, increased LV levels of manganese superoxide dismutase 2 (SOD2), and stimulated nuclear protein levels of Nrf2 [29]. Although the increase in SOD2 was attributed to total LV lysates [29], which primarily included cardiomyocytes, it was consistent with our results using only cultured D-HCAECs (Figure 5B). Moreover, ECs rely on glycolysis rather than oxidative phosphorylation for ATP production [30], which is why we observed increased cytosolic ROS and SOD1 levels.

Another component of our pequi extract that has been previously associated with the induction of Nrf2/HO-1 and modulation of endogenous antioxidant enzymes in diabetes is quercetin. For instance, in diabetic male Wistar rats fed a high-fat diet, quercetin caused a significant increase in the SOD and CAT cardiac tissue activities [31]. Additionally, in hyperglycemic rats fed a high-cholesterol (HC) diet, quercetin increased the activity of SOD1, CAT, and GSH peroxidase by 75%, 163%, and 77%, respectively [32]. This report is quite substantial, given that the same endogenous antioxidant enzymes are upregulated by the pequi extract, as revealed in our results.

Our findings further support the growing evidence that positions plant-derived polyphenols as potential nutraceuticals for cardiovascular health. The antioxidant and pro-proliferative effects of pequi bark extract observed in diabetic endothelial cells align with the concept of nutraceuticals as natural products capable of preventing or mitigating chronic disease through molecular mechanisms related to redox balance and vascular homeostasis [33]. Considering the increasing prevalence of Type 2 diabetes mellitus (T2DM) and its vascular complications, the incorporation of pequi extract into nutraceutical strategies may represent a complementary approach to conventional therapies, warranting further translational and clinical investigation.

**Figure 6:**
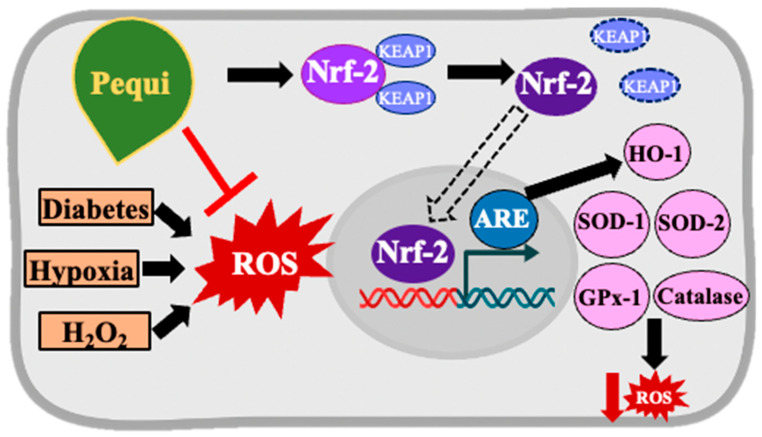
Proposed mechanism of pequi extract to decrease ROS production in diabetic human coronary artery endothelial cells (D-HCAECs) subjected to oxidative stress conditions. Oxidative stress, promoted by H2O2, hypoxia, or diabetes, stimulates the Nrf2 pathway, an effect that is enhanced when pequi extract is added to the media. Nrf2 uncouples from the Nrf2-Keap1 complex, translocates to the nucleus, binds to ARE, and promotes the transcription of mRNA for antioxidant enzymes, thereby decreasing ROS levels. Nrf2—nuclear factor (erythroid-derived 2)-like 2; ARE—antioxidant response element; ROS—reactive oxygen species; HO-1—phase II detoxifying enzyme heme-oxygenase-1; SOD—superoxide dismutase; GPx—glutathione peroxidase.

Taken together, our results demonstrate that the polyphenol-rich extract of a native Brazilian plant has decreased ROS levels, increased proliferation, and enhanced the expression of the Nrf2/HO-1 pathway, while also stimulating the endogenous antioxidant enzymes SOD1, SOD2, CAT, and GPx in coronary artery endothelial cells obtained from patients with diabetes. Further investigation is necessary to unveil the interaction between the Nrf2 and glucose pathways and the effects of the extract in diabetic endothelial cells, as proposed in Figure 6. Pequi extract may develop into an alternative, natural treatment for diabetes-related oxidative stress morbidity in the cardiovascular system.

### Limitations

Nevertheless, this study has limitations since it was restricted to in vitro experiments using diabetic human coronary artery endothelial cells. Although our findings demonstrate reduced ROS and enhanced antioxidant enzyme expression, they may not fully reflect the complex in vivo environment where endocrine, immunological, and metabolic interactions occur and should be addressed in future experiments. Additionally, only two concentrations of extract (10 and 25 μg/mL) were tested; broader dose–response evaluations should be conducted in a subsequent experiment.

Finally, we did not assess antioxidant enzyme expression under hypoxic conditions due to technical variability, which remains a priority for future work. The experiments are beyond the scope of the current experiment and will be performed in a future study.

## 5. Conclusions

In conclusion, the ethanolic extract obtained from the bark of the pequi (*Caryocar brasiliense*) induces proliferation. It promotes the drastic reduction in cytosolic ROS levels in diabetic human coronary artery endothelial cells subjected to oxidative stress conditions while prompting the expression of the Nrf2/HO-1 pathway and increasing the levels of the endogenous antioxidant defense enzymes superoxide dismutase 1, catalase, and glutathione peroxidase. These findings point to a potential in vivo antioxidant effect of the pequi extract in diabetes and ischemic heart disease.

## Figures and Tables

**Table 1:** Characterization of the main compounds present in the pequi (*Caryocar brasiliense*) bark ethanolic extract by HPLC-MS.

Identified Compound	Molecular Formula	RT (Min)	Standard RT (Min)	Detected Mass	Calculated Mass	Error (ppm)	Fragments (*m*/*z*)
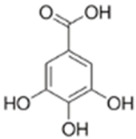 Gallic Acid	C_7_H_6_O_5_	14.25	14.26	169.01349	169.01370	2.013	169.01349 125.02338
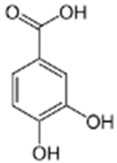 Protocatechuic Acid	C_7_H_6_O_4_	19.09	19.15	153.01843	153.01879	1.273	153.01843 109.0283
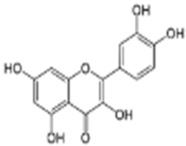 Quercetin	C_15_H_10_O_7_	27.78	27.78	301.03561	301.03561	4.585	301.03561 178.99792 151.00279
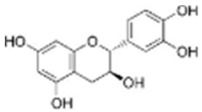 Catechin	C_15_H_14_O_6_	20.05	20.01	289.07199	289.07122	4.585	289.07199

## Data Availability

The original contributions presented in this study are included in the article/Supplementary Materials. Further inquiries can be directed to the corresponding author(s).

## Supplementary Materials

The following supporting information can be downloaded at: https://www.mdpi.com/article/10.3390/antiox14101167/s1.

## Abbreviations

The following abbreviations are used in this manuscript:

ROS	Reactive oxygen species
DM	Diabetes mellitus
T2DM	Type 2 diabetes mellitus
EC/ECs	Endothelial cell(s)
HCAEC	Human coronary artery endothelial cell
D-HCAEC	Diabetic human coronary artery endothelial cell
mito-ROS	Mitochondrial reactive oxygen species
Nrf2	Nuclear factor (erythroid-derived 2)-like 2
HO-1	Heme-oxygenase-1
SOD1	Superoxide dismutase 1
SOD2	Superoxide dismutase 2
GPx1/GPx	Glutathione peroxidase 1
CAT	Catalase
DAPI	4′,6-Diamidino-2-Phenylindole (nuclear stain)
DCF	2′,7′-Dichlorofluorescein
H2DCF-DA	2,7-Dichlorodihydrofluorescein diacetate
DMSO	Dimethyl sulfoxide
HBSS	Hank’s Balanced Salt Solution
MIC	Modular Incubator Chamber
WB	Western blot
ANOVA	Analysis of variance
ARE	Antioxidant response element
KEAP1	Kelch-like ECH-associated protein 1
HPLC-HRMS	High-Pressure Liquid Chromatography–High-Resolution Mass Spectrometry
LV	Left ventricle
ATP	Adenosine Triphosphate
HUVECs	Human umbilical vein endothelial cells
NIH	National Institutes of Health
