# Supplementary material for: Pequi (Caryocar brasiliense, Camb) Bark Extract Reduces ROS Production in Diabetic Human Coronary Endothelial Cells
Source: Antioxidants (Basel). 2025 Sep 25;14(10):1167. doi: 10.3390/antiox14101167 (PMC12562227; doi:10.3390/antiox14101167)

C:\Users\...\Claudiane\A'

17/07/2018 18:47:58

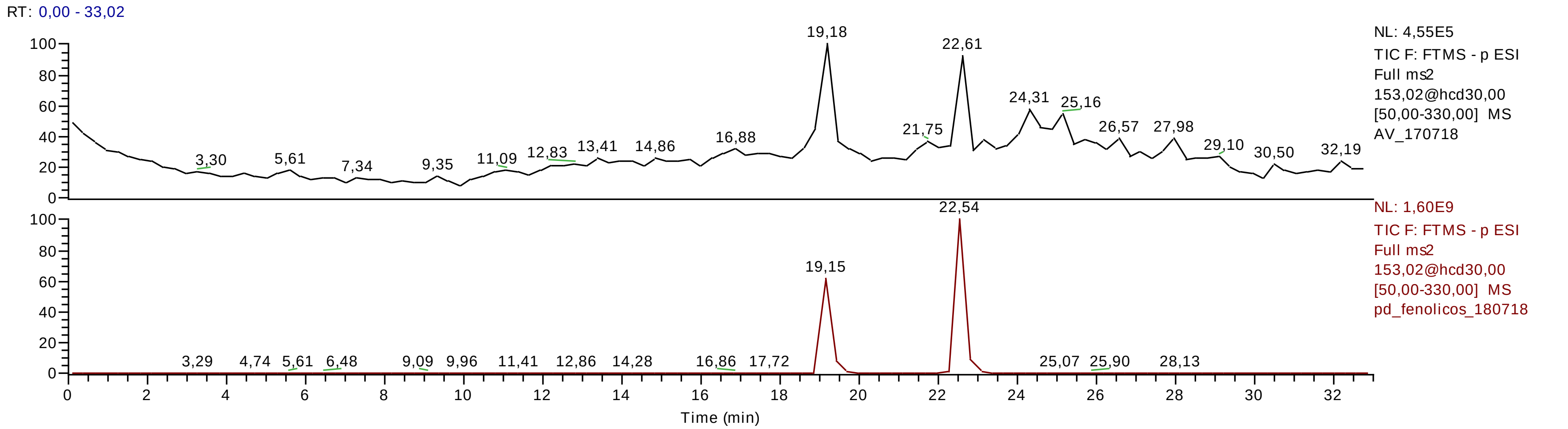

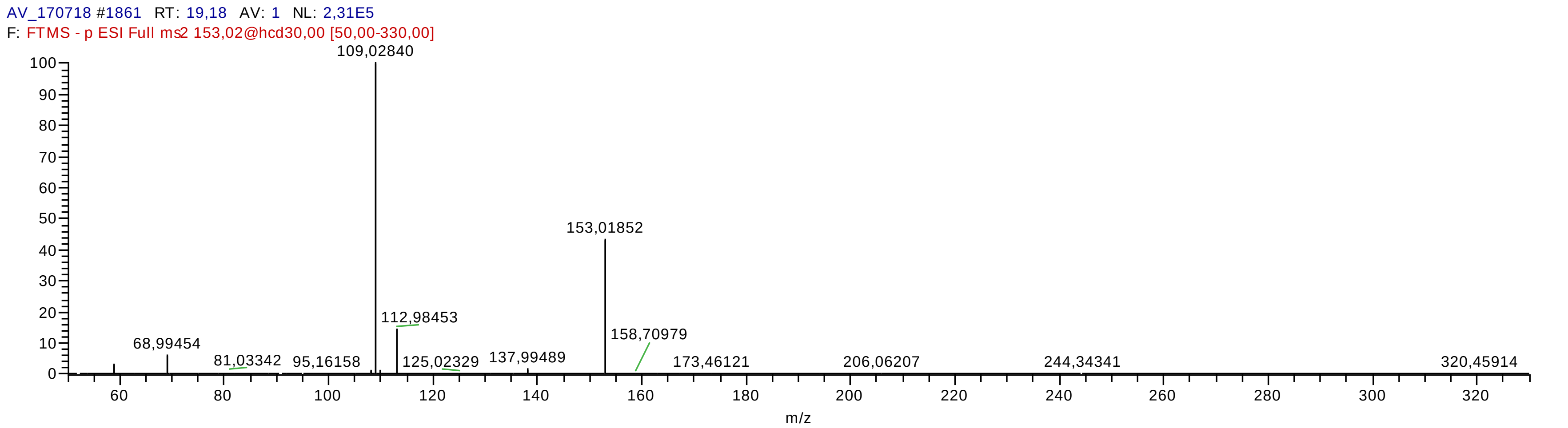

C:\Users\...\Claudian

17/07/2018 18:47:58

RT: 0,00 - 33,02

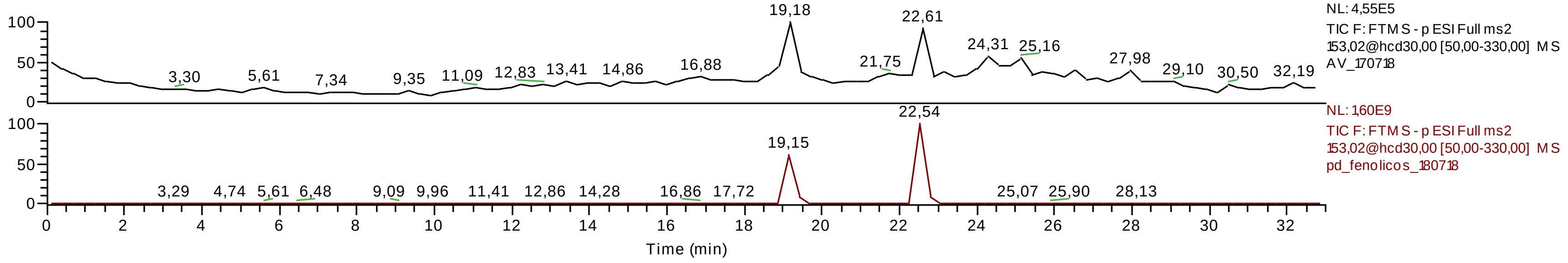

AV_170718 #2197 RT: 22,61 AV: 1 NL: 1,40E5
F: FTMS - p ESI Full ms2 153,02@hcd30,00 [50,00-330,00]

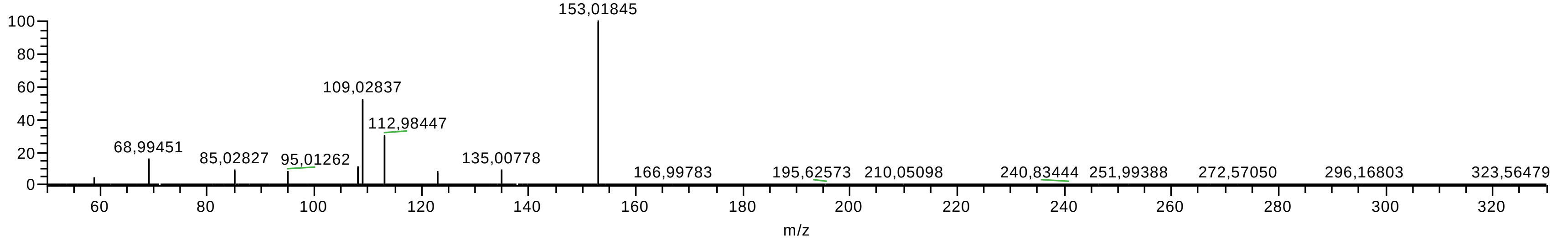

pd_fenolicos_180718 #2197 RT: 22,54 AV: 1 NL: 6,32E8
F: FTMS - p ESI Full ms2 153,02@hcd30,00 [50,00-330,00]

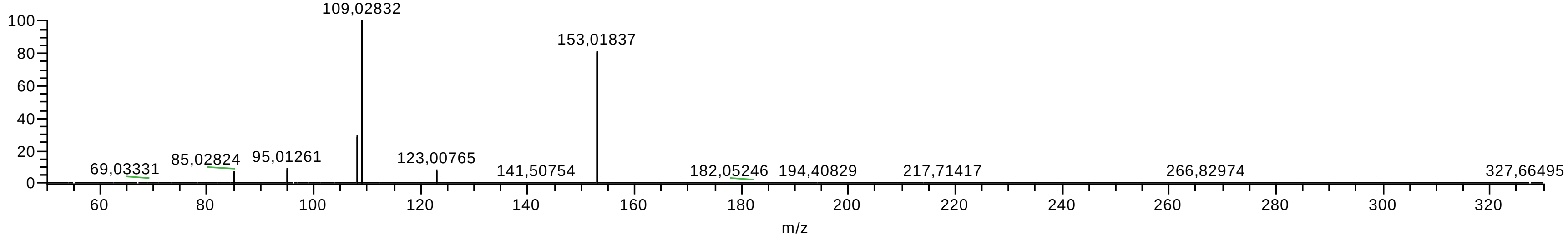

C:\Users\...\Claudiane\AV 17/07/2018 18:47:58

RT: 0,00 - 33,02

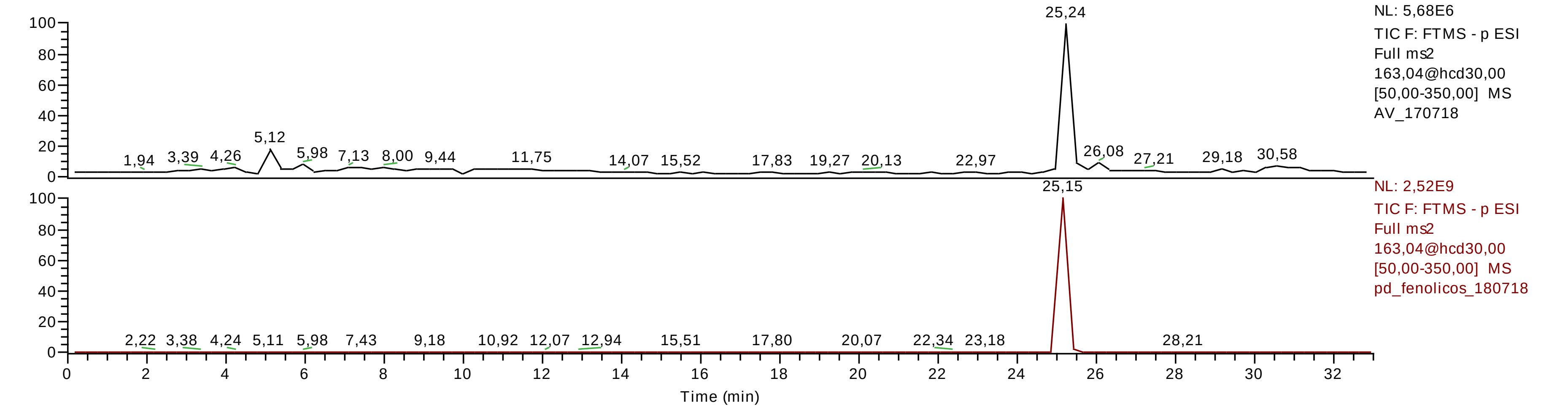

AV_170718 #2457 RT: 25,24 AV: 1 NL: 4,32E6
F: FTMS - p ESI Full ms2 163,04@hcd30,00 [50,00-350,00]

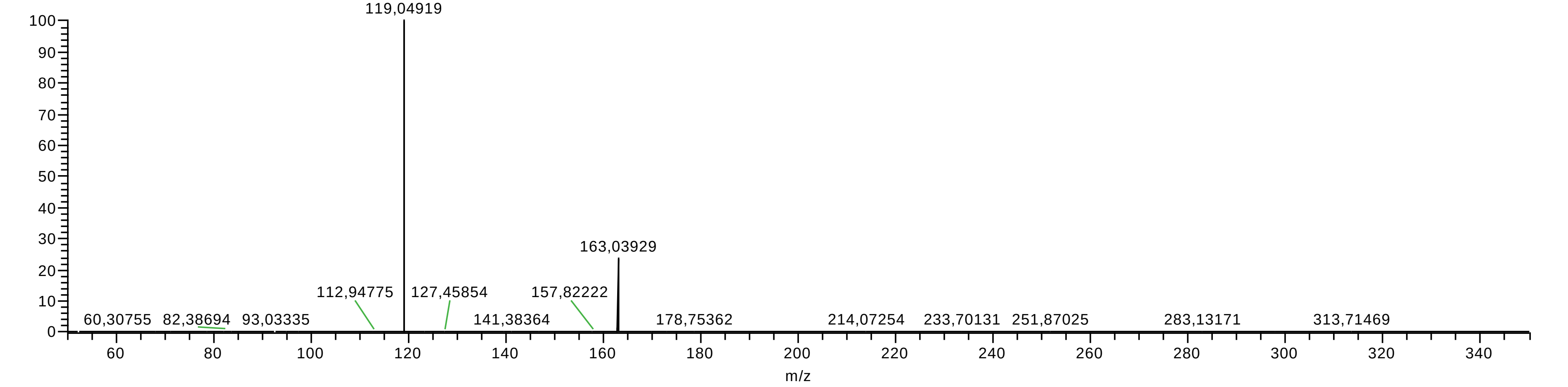

C:\Users\...\Claudian 17/07/2018 18:47:58

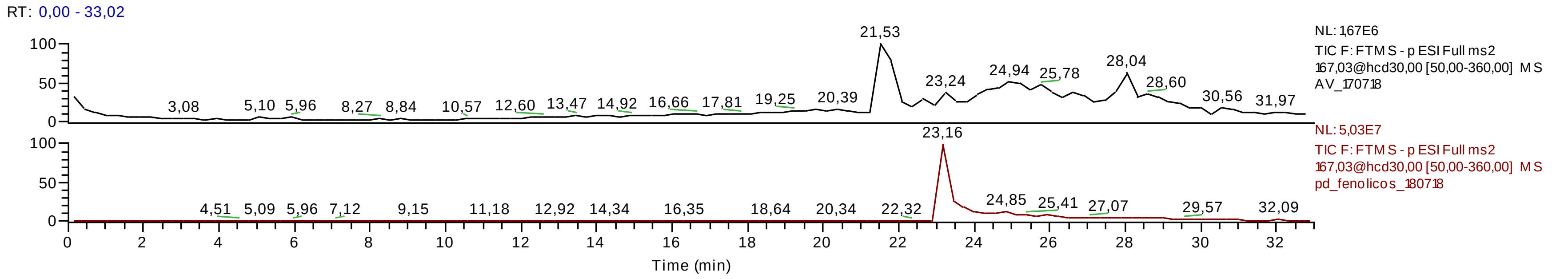

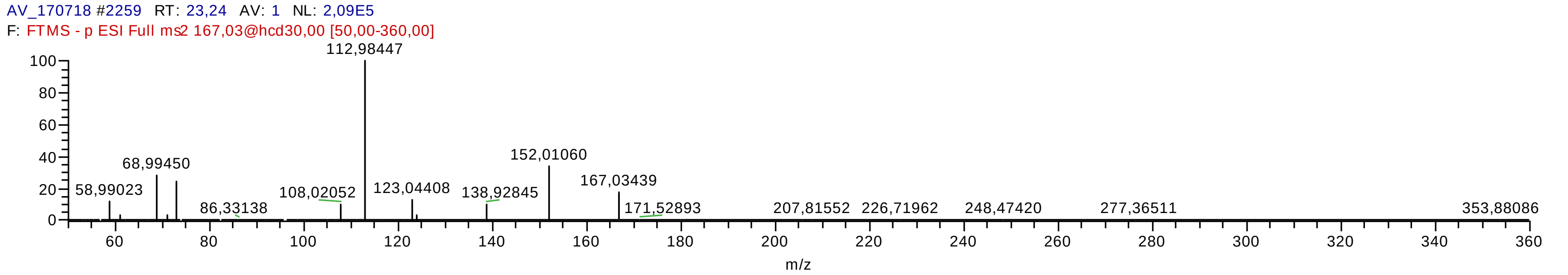

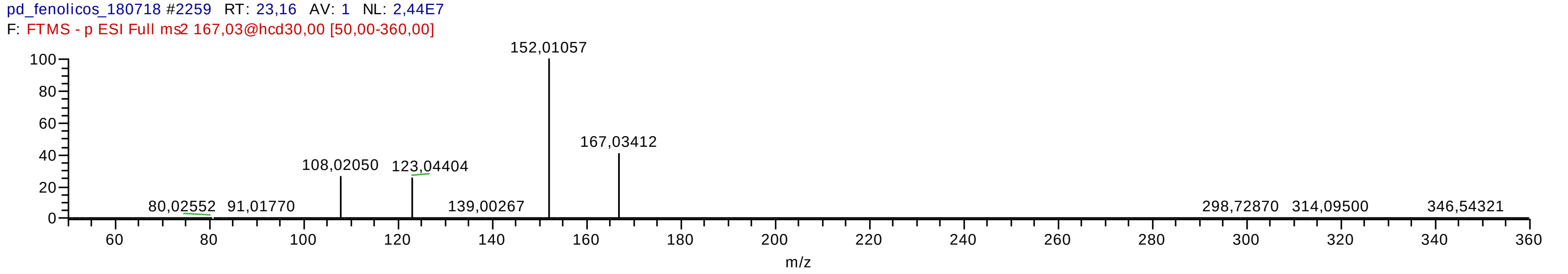

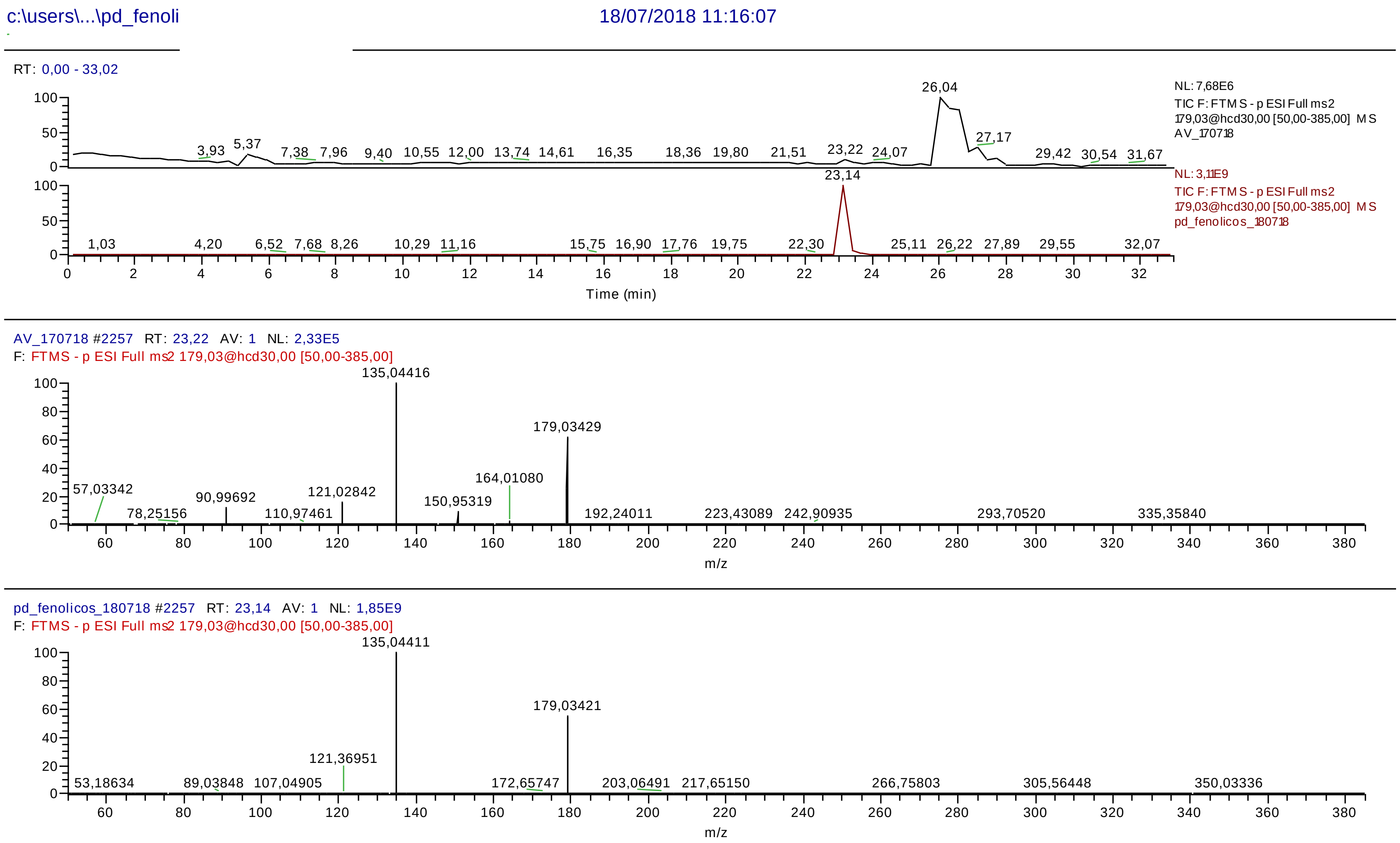
c:\users\...\pd_fenoli
18/07/2018 11:16:07
RT: 0,00 - 33,02
NL: 7,68E6
TIC F: FTMS - p ESI Full ms2 179,03@hcd30,00 [50,00-385,00] MS AV_170718
NL: 3,11E9
TIC F: FTMS - p ESI Full ms2 179,03@hcd30,00 [50,00-385,00] MS pd_fenolicos_180718
Time (min)
AV_170718 #2257 RT: 23,22 AV: 1 NL: 2,33E5
F: FTMS - p ESI Full ms2 179,03@hcd30,00 [50,00-385,00]
135,04416
179,03429
164,01080
57,03342
121,02842
90,99692
150,95319
78,25156
110,97461
192,24011
223,43089
242,90935
293,70520
335,35840
m/z
pd_fenolicos_180718 #2257 RT: 23,14 AV: 1 NL: 1,85E9
F: FTMS - p ESI Full ms2 179,03@hcd30,00 [50,00-385,00]
135,04411
179,03421
121,36951
53,18634
89,03848
107,04905
172,65747
203,06491
217,65150
266,75803
305,56448
350,03336
m/z

C:\Users\...\Claudian

17/07/2018 18:47:58

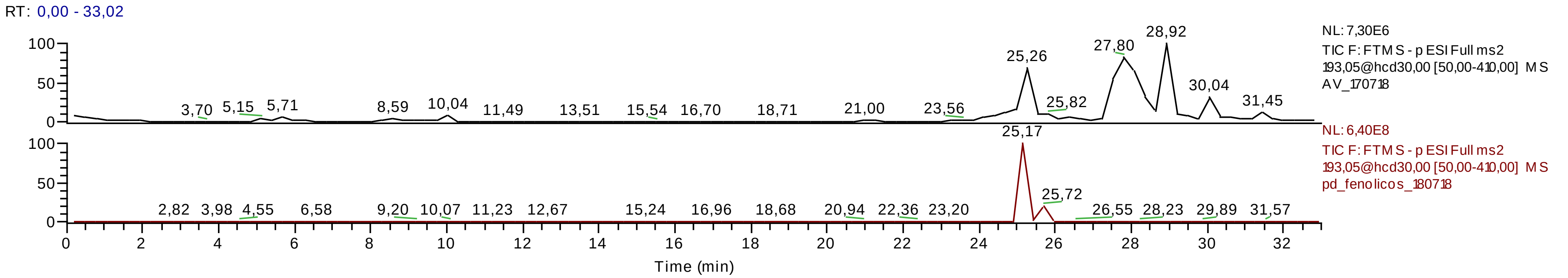

AV_170718 #2459 RT: 25,26 AV: 1 NL: 1,38E6
F: FTMS - p ESI Full ms2 193,05@hcd30,00 [50,00-410,00]

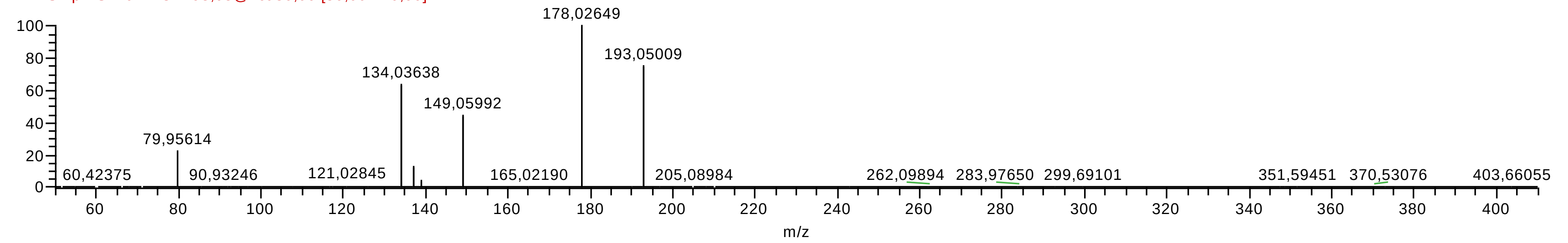

pd_fenolicos_180718 #2459 RT: 25,17 AV: 1 NL: 2,16E8
F: FTMS - p ESI Full ms2 193,05@hcd30,00 [50,00-410,00]

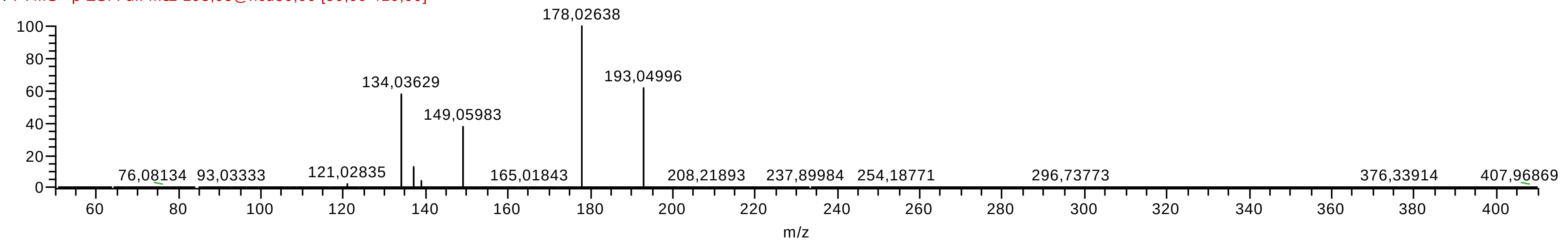

C:\Users\...\Claudiane\A\' 17/07/2018 18:47:58

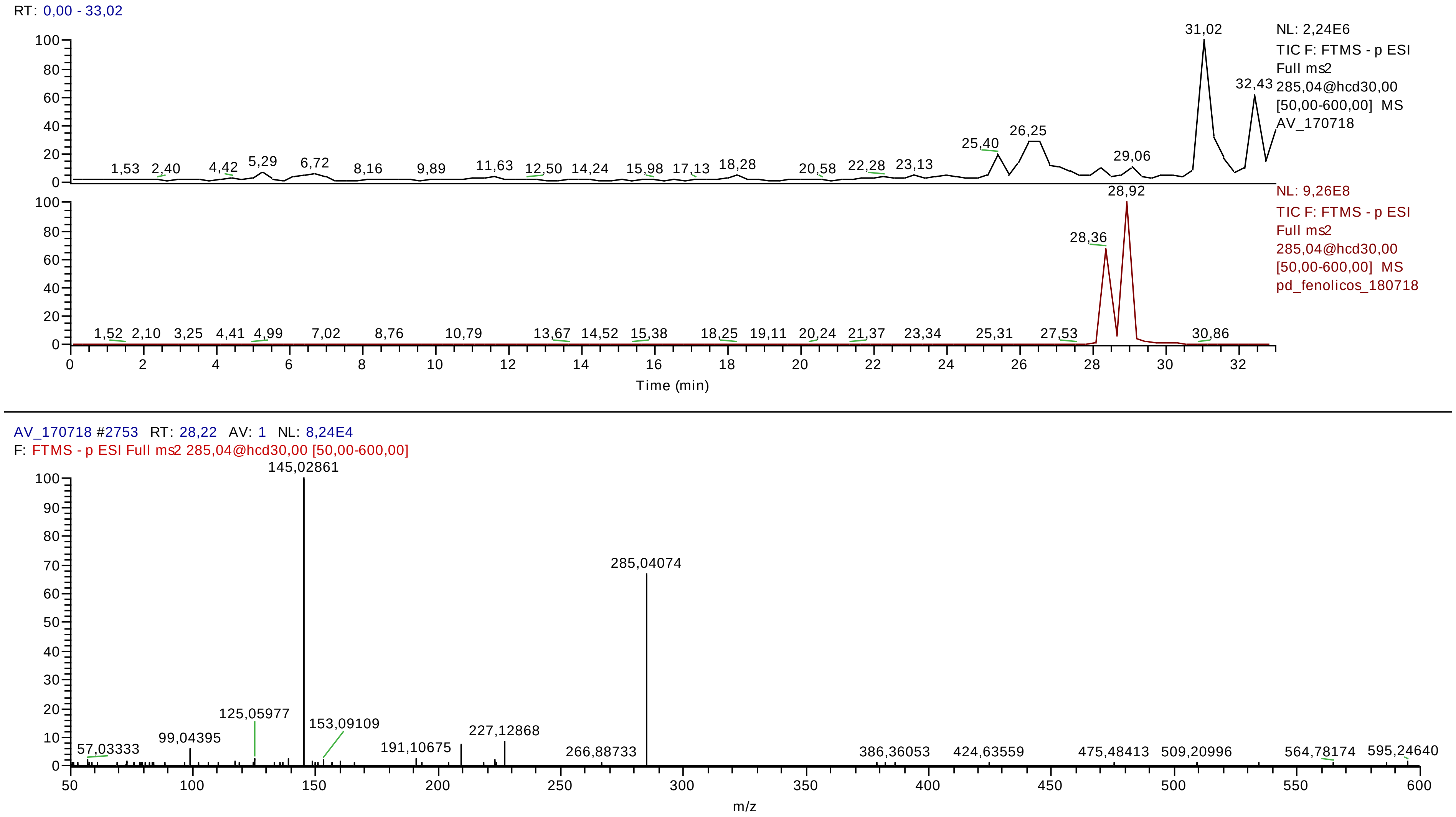

c:\users\...\pd_fenoli 18/07/2018 11:16:07

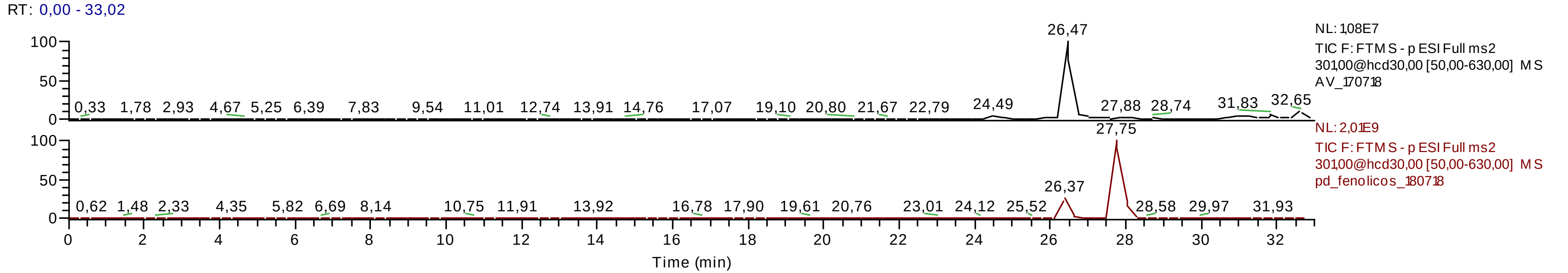

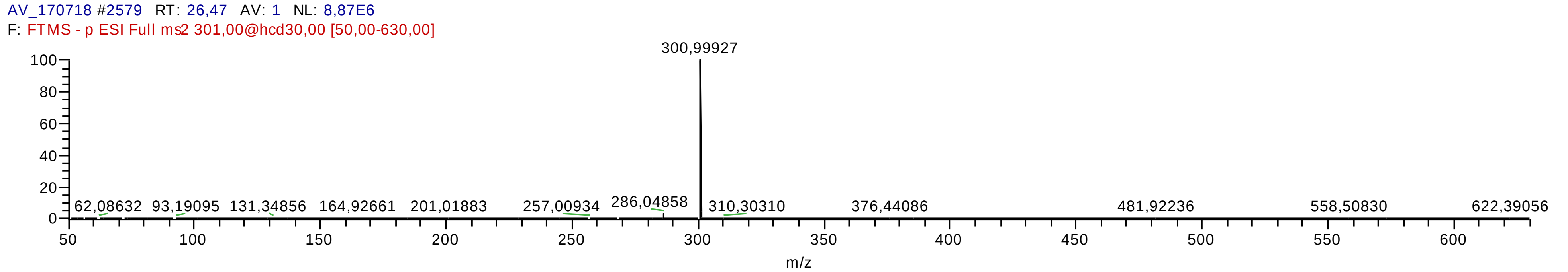

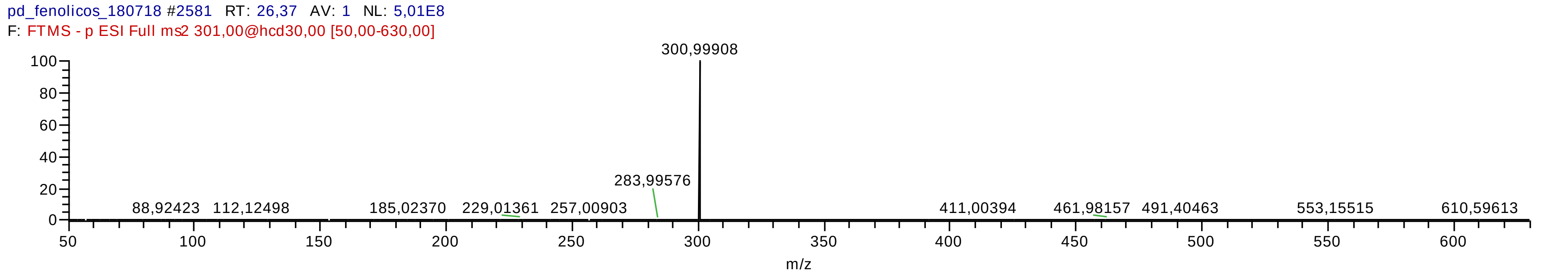

C:\Users\...\Claudiane\AV_17071[illegible] 17/07/2018 18:47:58

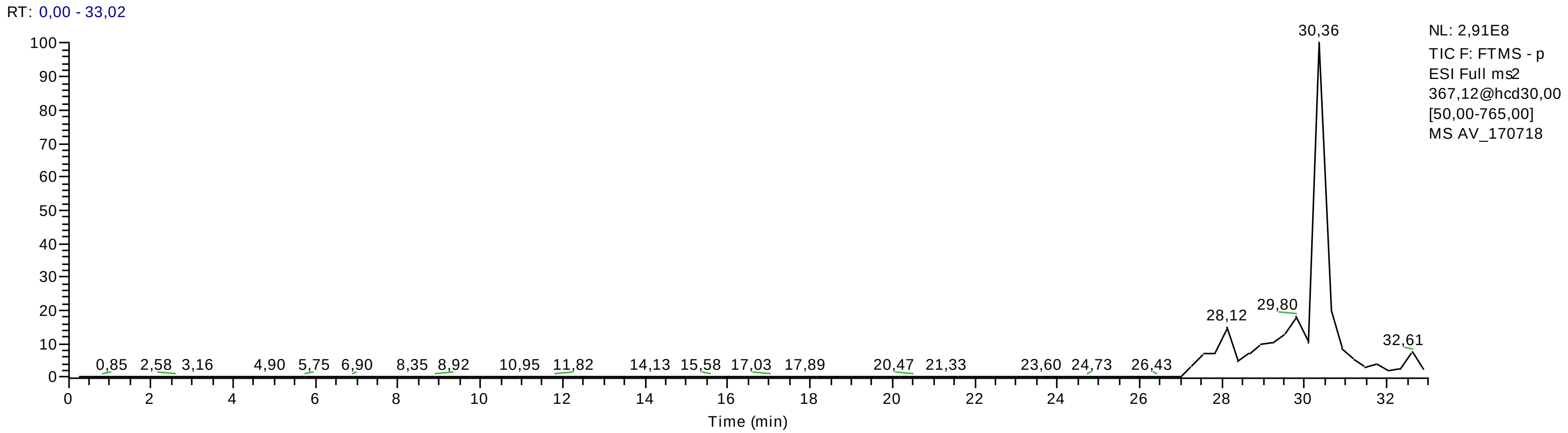

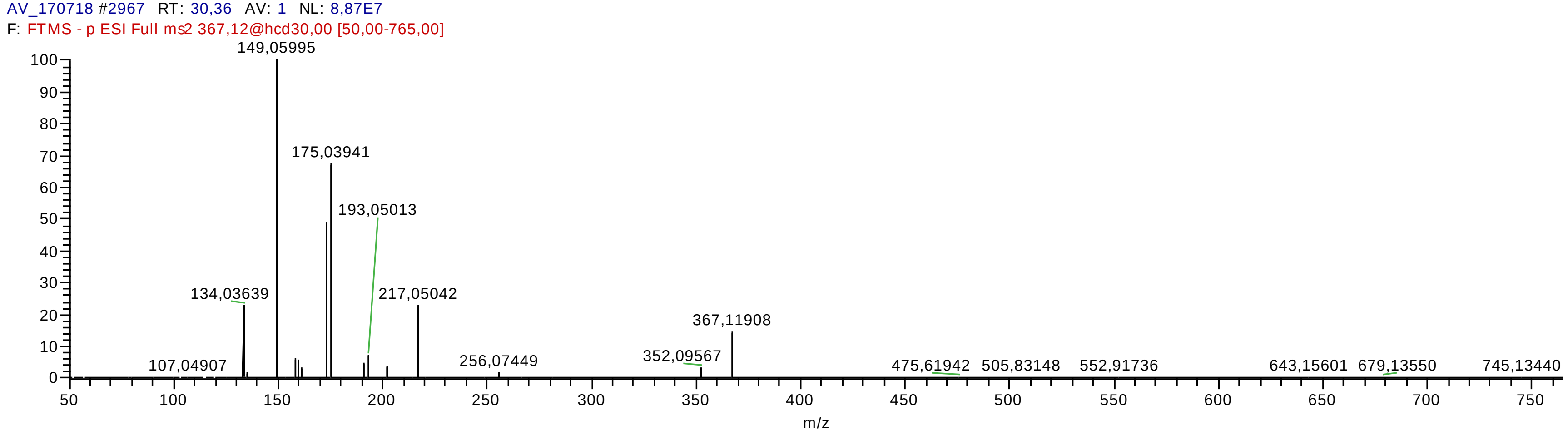

C:\Users\...\Claudiane\A\ 17/07/2018 10:45:28

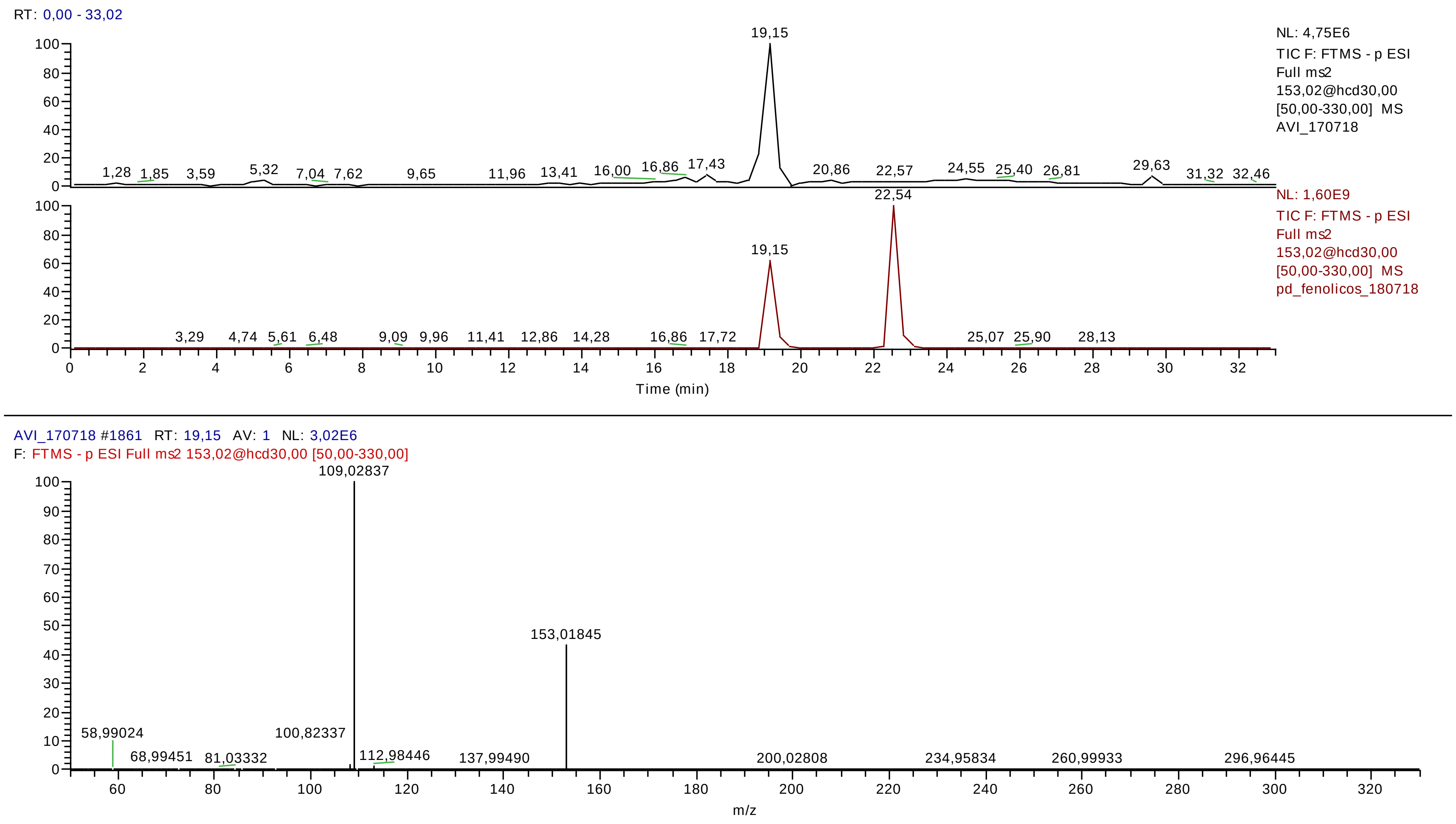

C:\Users\...\Claudian

17/07/2018 10:45:28

RT: 0,00 - 33,02

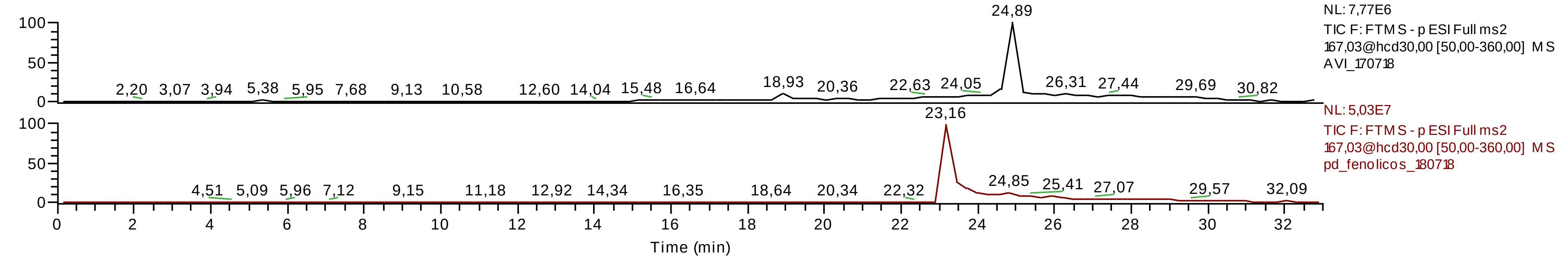

AVI_170718 #2427 RT: 24,89 AV: 1 NL: 5,57E6
F: FTMS - p ESI Full ms2 167,03@hcd30,00 [50,00-360,00]

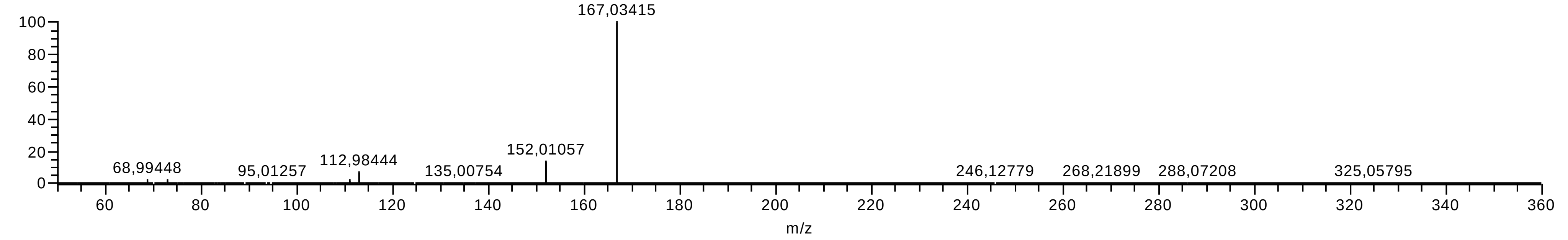

pd_fenolicos_180718 #2259 RT: 23,16 AV: 1 NL: 2,44E7
F: FTMS - p ESI Full ms2 167,03@hcd30,00 [50,00-360,00]

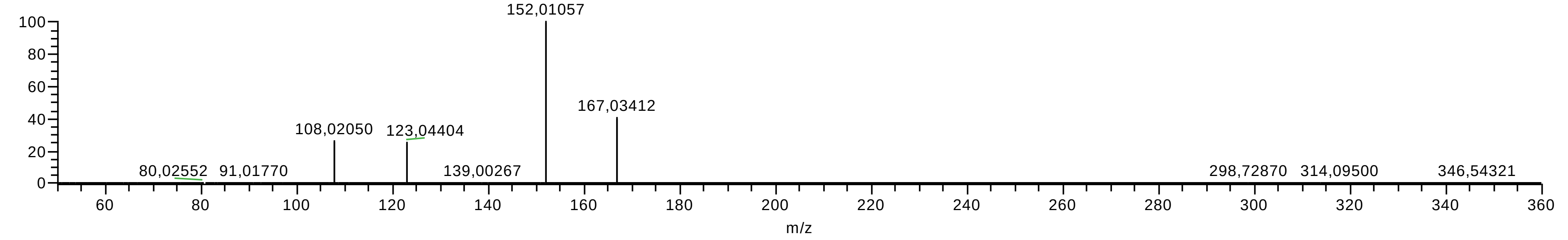

C:\Users\...\Claudiane\A\ 17/07/2018 10:45:28

RT: 0,00 - 33,02

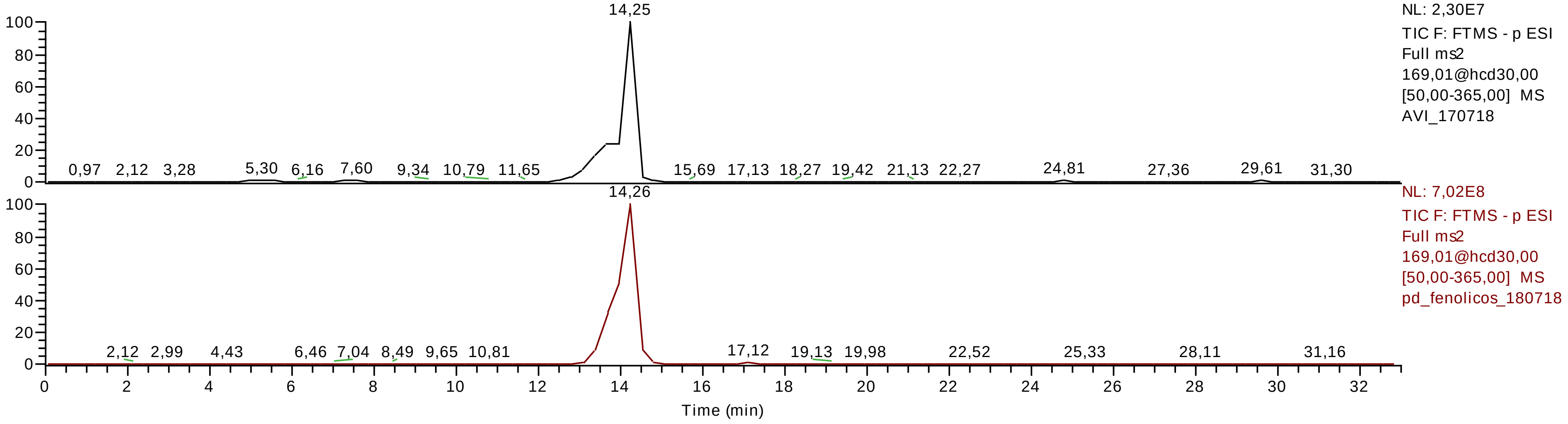

AVI_170718 #1383 RT: 14,25 AV: 1 NL: 1,14E7
F: FTMS - p ESI Full ms2 169,01@hcd30,00 [50,00-365,00]

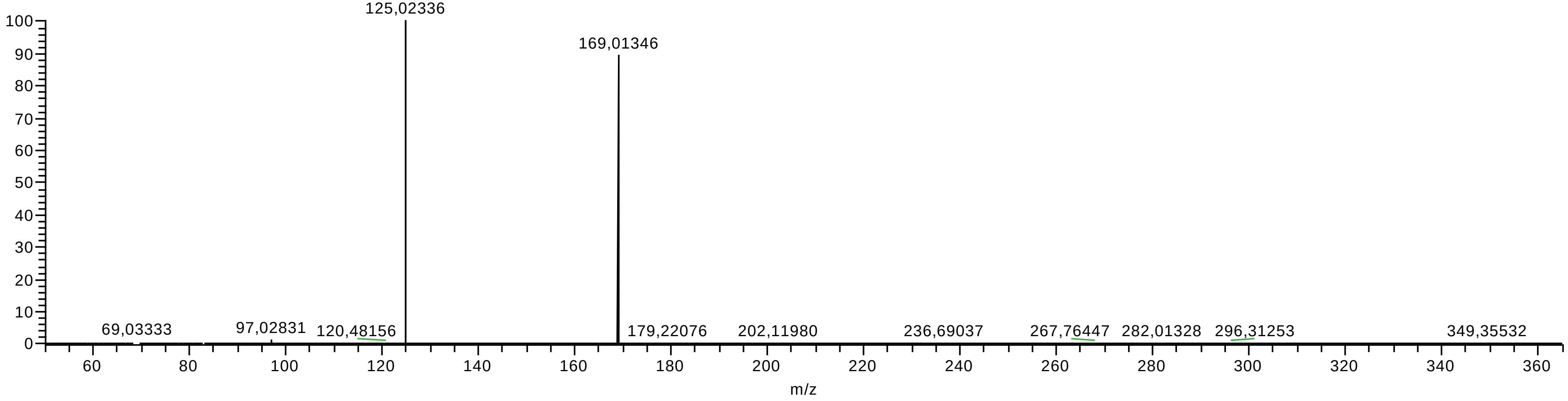

C:\Users\...\Claudian

17/07/2018 10:45:28

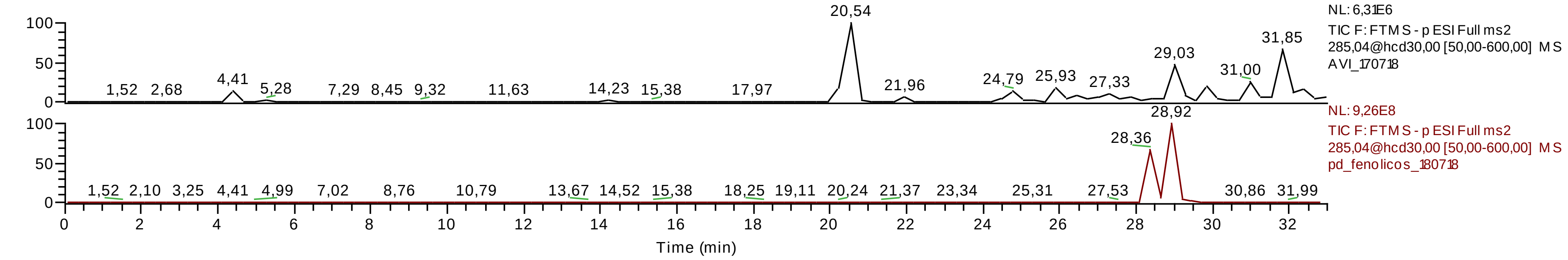

AVI_170718 #2837 RT: 29,03 AV: 1 NL: 1,82E6
F: FTMS - p ESI Full ms2 285,04@hcd30,00 [50,00-600,00]

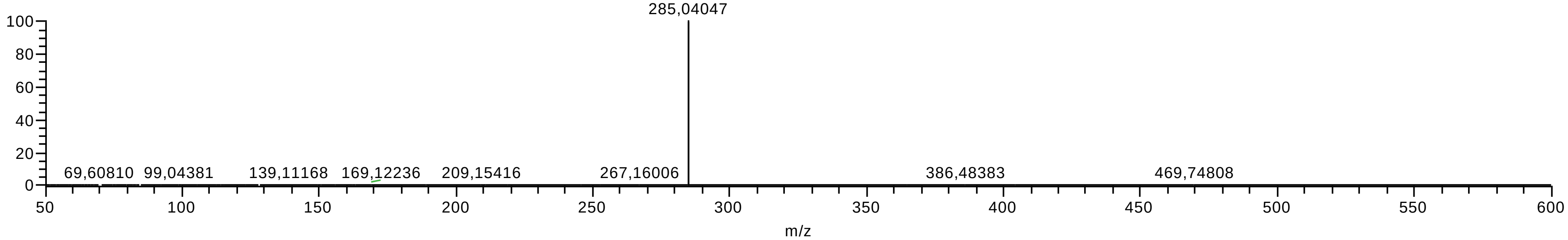

pd_fenolicos_180718 #2837 RT: 28,92 AV: 1 NL: 8,20E8
F: FTMS - p ESI Full ms2 285,04@hcd30,00 [50,00-600,00]

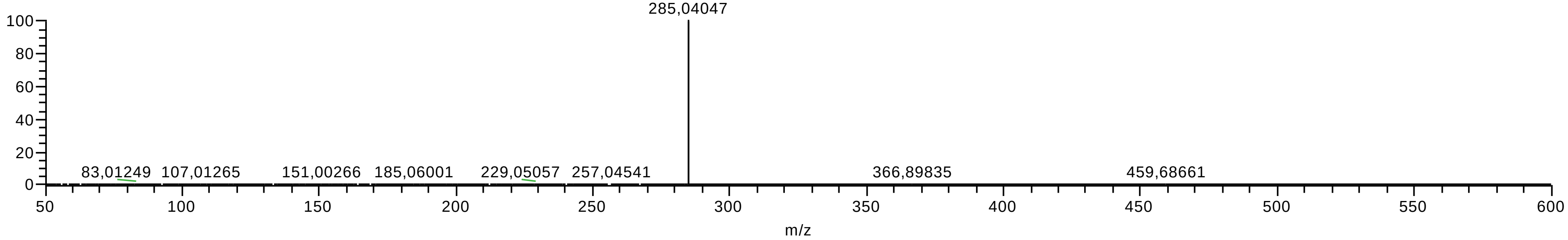

c:\users\...\pd_fenoli 18/07/2018 11:16:07

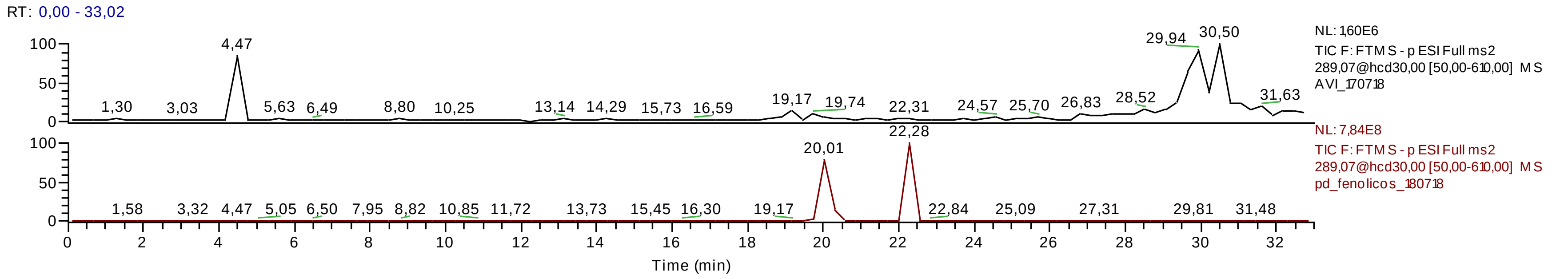

AVI_170718 #1947 RT: 20,03 AV: 1 NL: 2,03E4
F: FTMS - p ESI Full ms2 289,07@hcd30,00 [50,00-610,00]

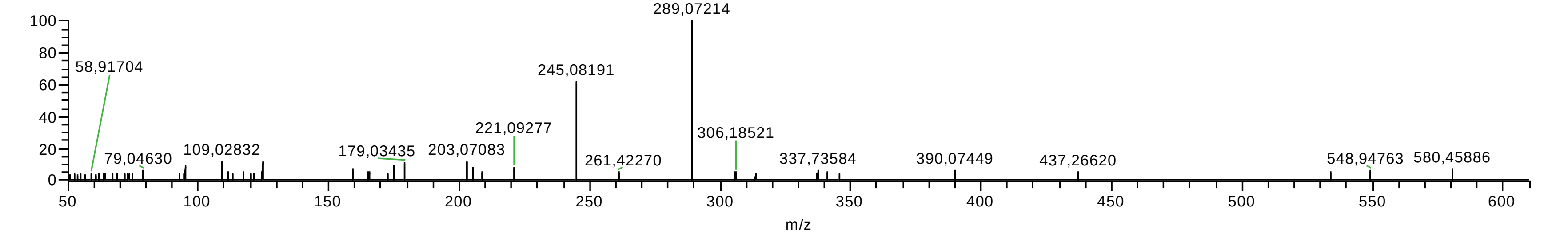

pd_fenolicos_180718 #1947 RT: 20,01 AV: 1 NL: 1,71E8
F: FTMS - p ESI Full ms2 289,07@hcd30,00 [50,00-610,00]

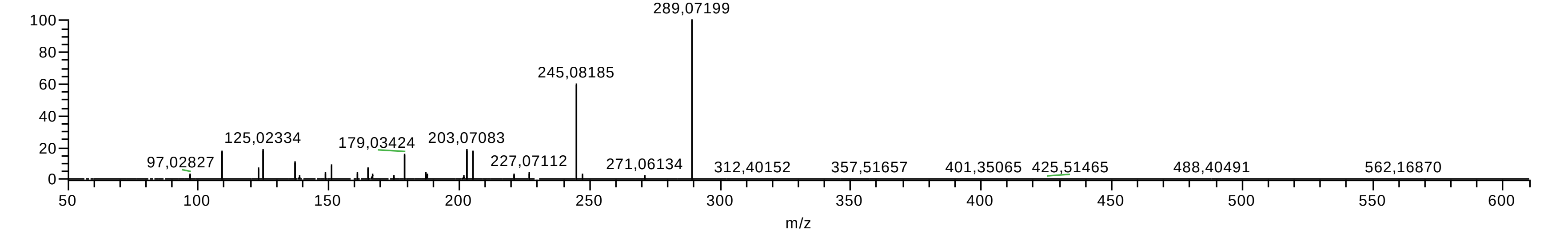

C:\Users\...\Claudian

17/07/2018 10:45:28

RT: 0,00 - 33,02

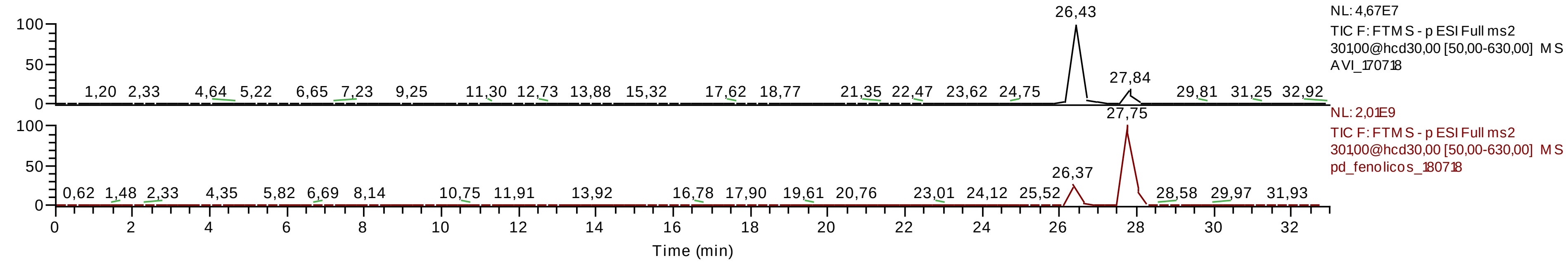

AVI_170718 #2579 RT: 26,43 AV: 1 NL: 4,36E7
F: FTMS - p ESI Full ms2 301,00@hcd30,00 [50,00-630,00]

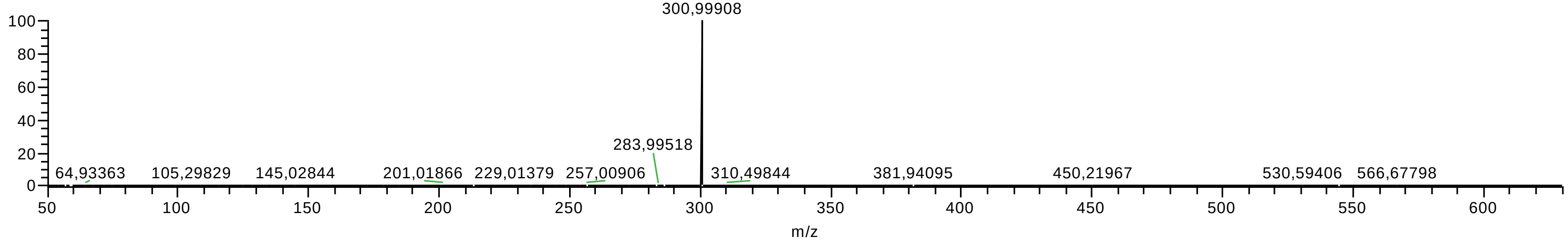

AVI_170718 #2579 RT: 26,43 AV: 1 NL: 1,48E5
F: FTMS - p ESI Full ms2 301,00@hcd30,00 [50,00-630,00]

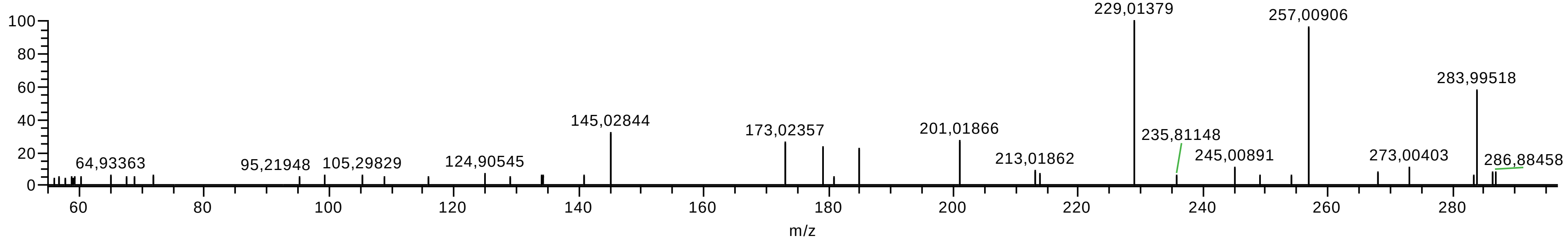

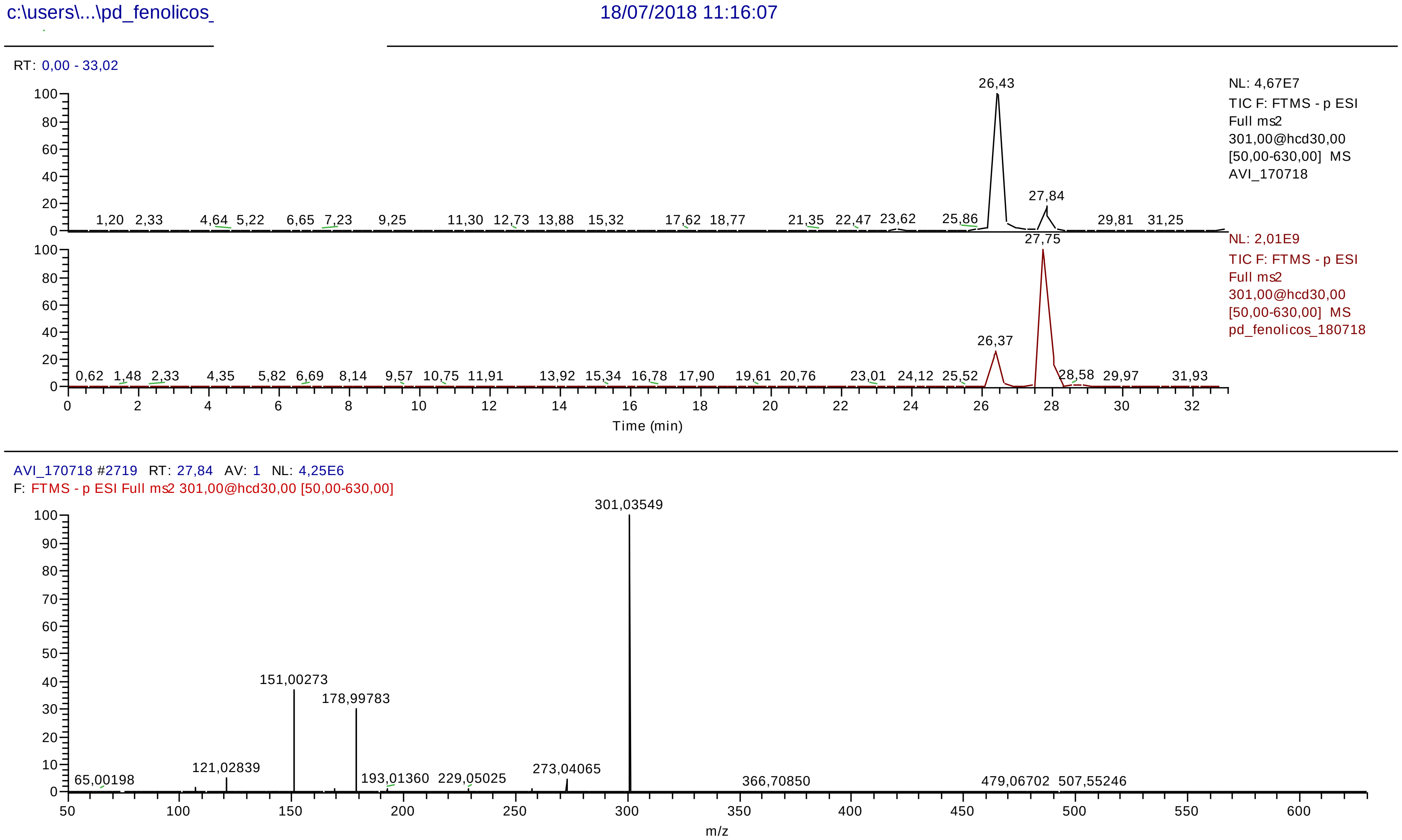

c:\users\...\pd_fenolicos_
18/07/2018 11:16:07
RT: 0,00 - 33,02
NL: 4,67E7
TIC F: FTMS - p ESI Full ms2 301,00@hcd30,00 [50,00-630,00] MS AVI_170718
26,43
27,84
1,20 2,33 4,64 5,22 6,65 7,23 9,25 11,30 12,73 13,88 15,32 17,62 18,77 21,35 22,47 23,62 25,86 29,81 31,25
NL: 2,01E9
TIC F: FTMS - p ESI Full ms2 301,00@hcd30,00 [50,00-630,00] MS pd_fenolicos_180718
27,75
26,37
0,62 1,48 2,33 4,35 5,82 6,69 8,14 9,57 10,75 11,91 13,92 15,34 16,78 17,90 19,61 20,76 23,01 24,12 25,52 28,58 29,97 31,93
Time (min)
AVI_170718 #2719 RT: 27,84 AV: 1 NL: 4,25E6
F: FTMS - p ESI Full ms2 301,00@hcd30,00 [50,00-630,00]
301,03549
151,00273
178,99783
65,00198
121,02839
193,01360
229,05025
273,04065
366,70850
479,06702
507,55246
m/z

C:\Users\...\Claudiane\A\ 17/07/2018 10:45:28

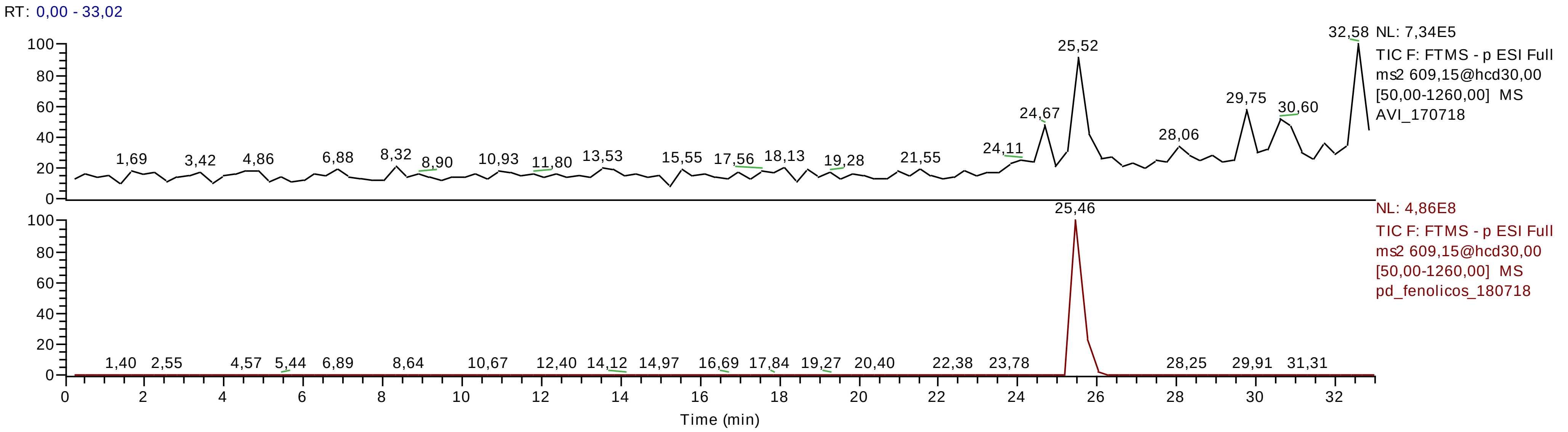

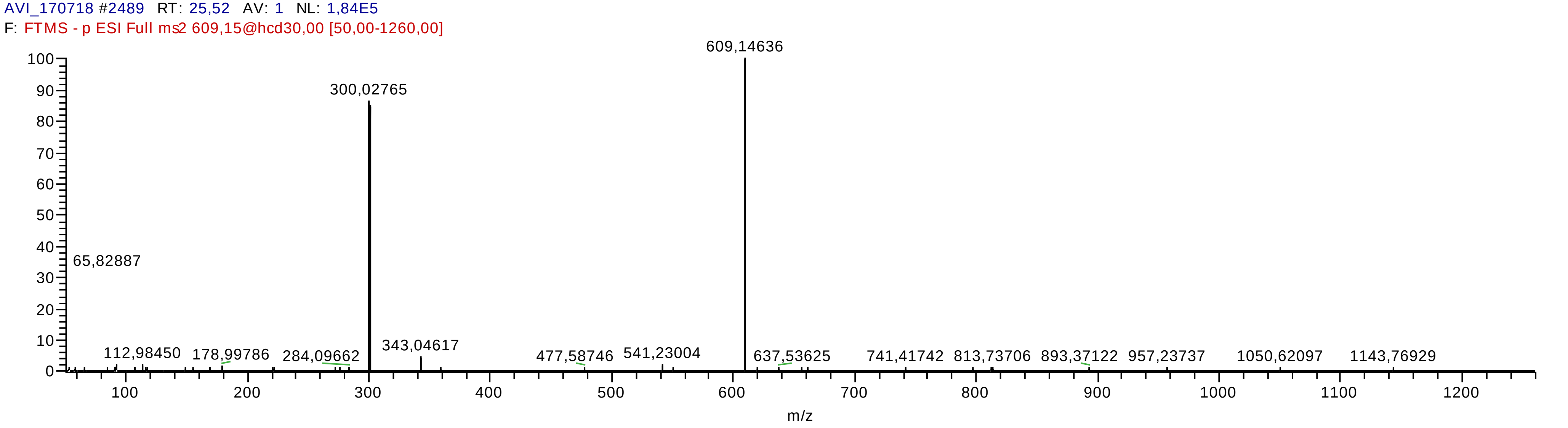

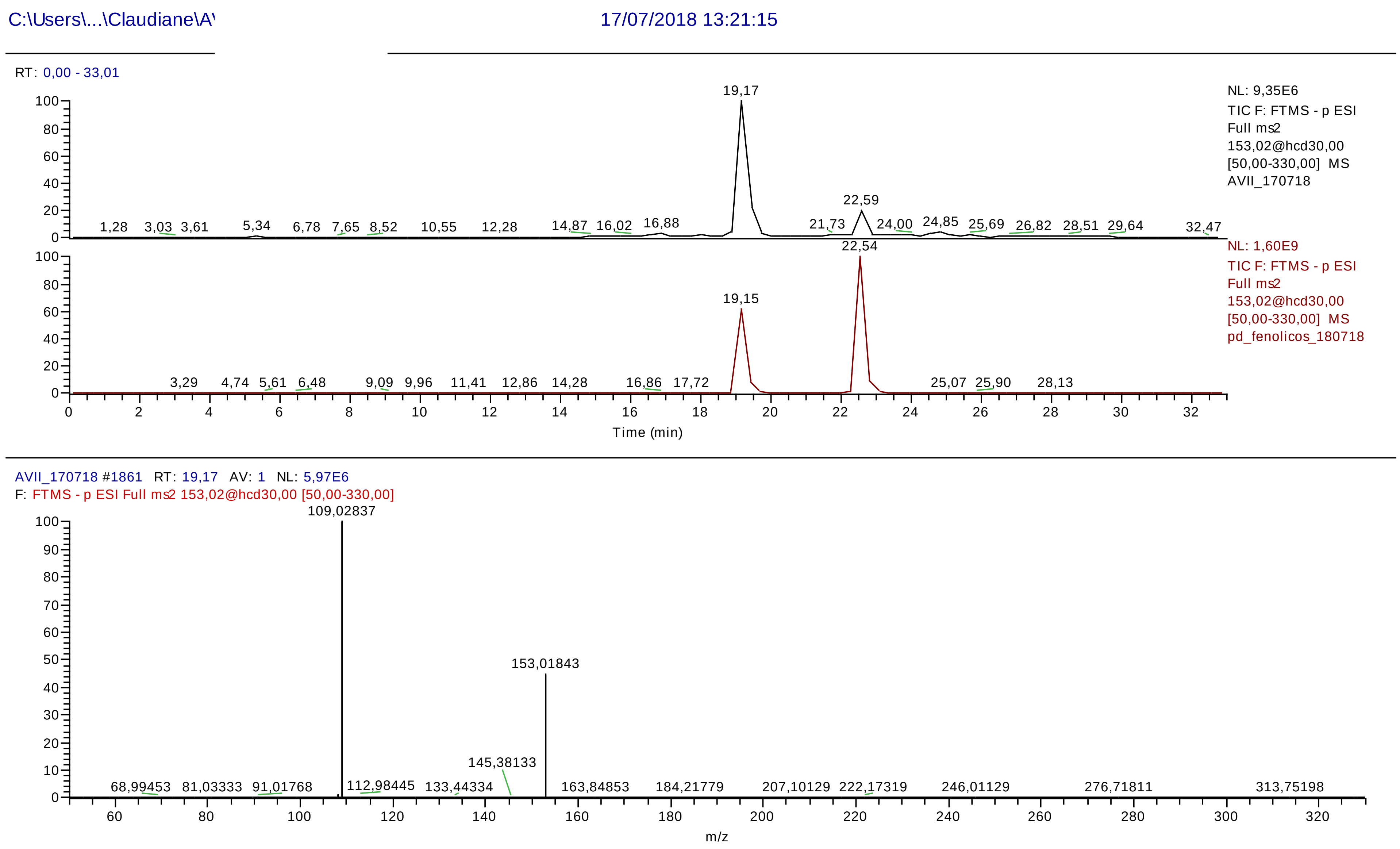

C:\Users\...\Claudiane\A\
17/07/2018 13:21:15
RT: 0,00 - 33,01
NL: 9,35E6
TIC F: FTMS - p ESI
Full ms2
153,02@hcd30,00
[50,00-330,00] MS
AVII_170718
19,17
22,59
NL: 1,60E9
TIC F: FTMS - p ESI
Full ms2
153,02@hcd30,00
[50,00-330,00] MS
pd_fenolicos_180718
19,15
22,54
Time (min)
AVII_170718 #1861 RT: 19,17 AV: 1 NL: 5,97E6
F: FTMS - p ESI Full ms2 153,02@hcd30,00 [50,00-330,00]
109,02837
153,01843
145,38133
68,99453
81,03333
91,01768
112,98445
133,44334
163,84853
184,21779
207,10129
222,17319
246,01129
276,71811
313,75198
m/z

C:\Users\...\Claudiane\A\ 17/07/2018 13:21:15

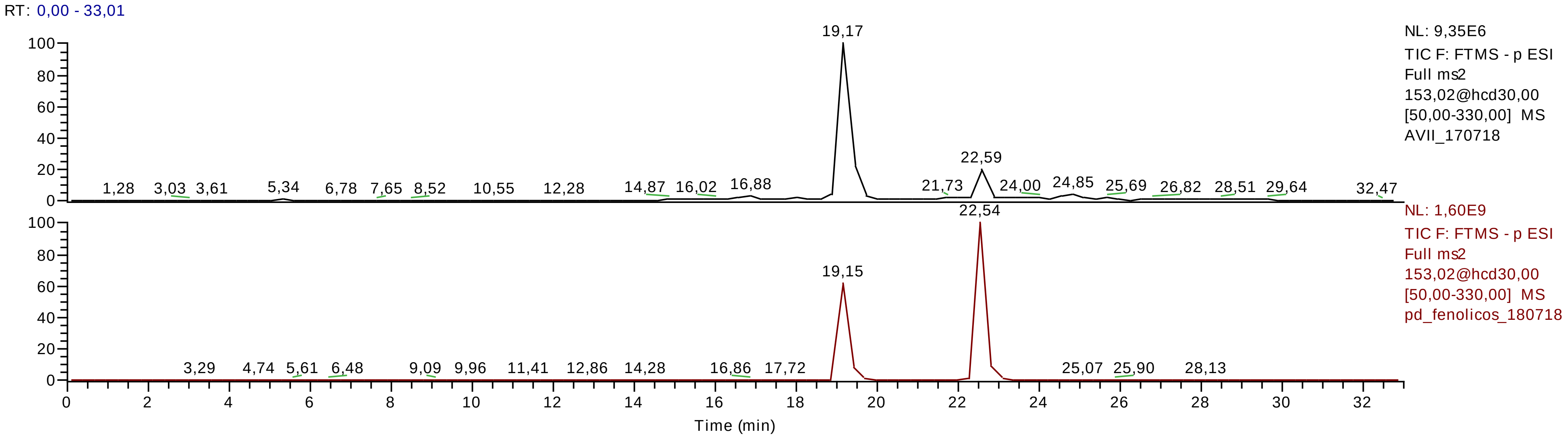

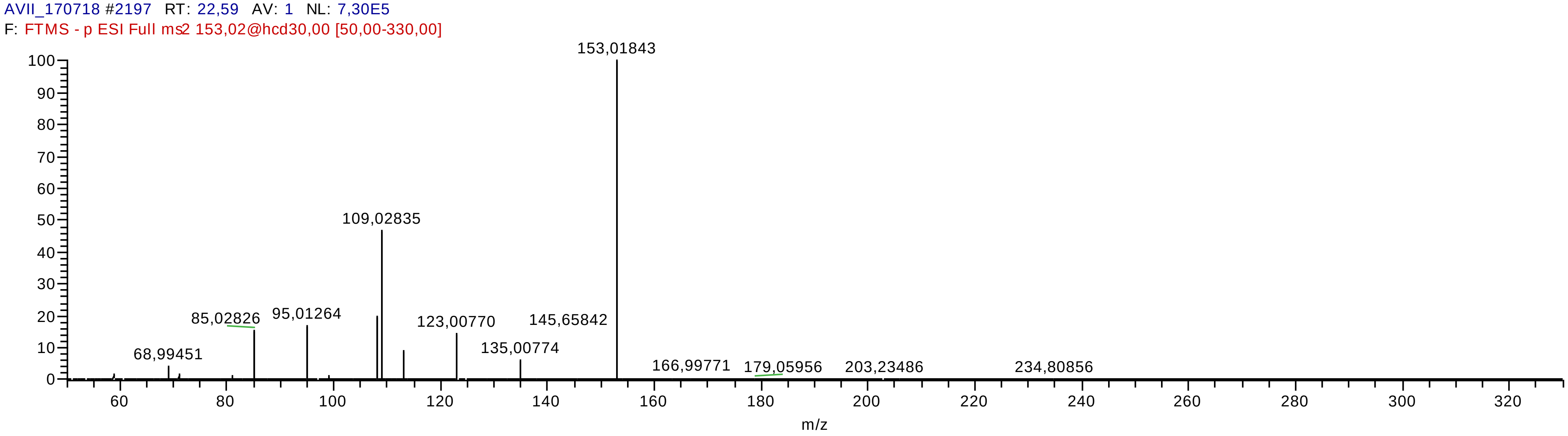

C:\Users\...\Claudiane\A\ 17/07/2018 13:21:15

RT: 0,00 - 33,01

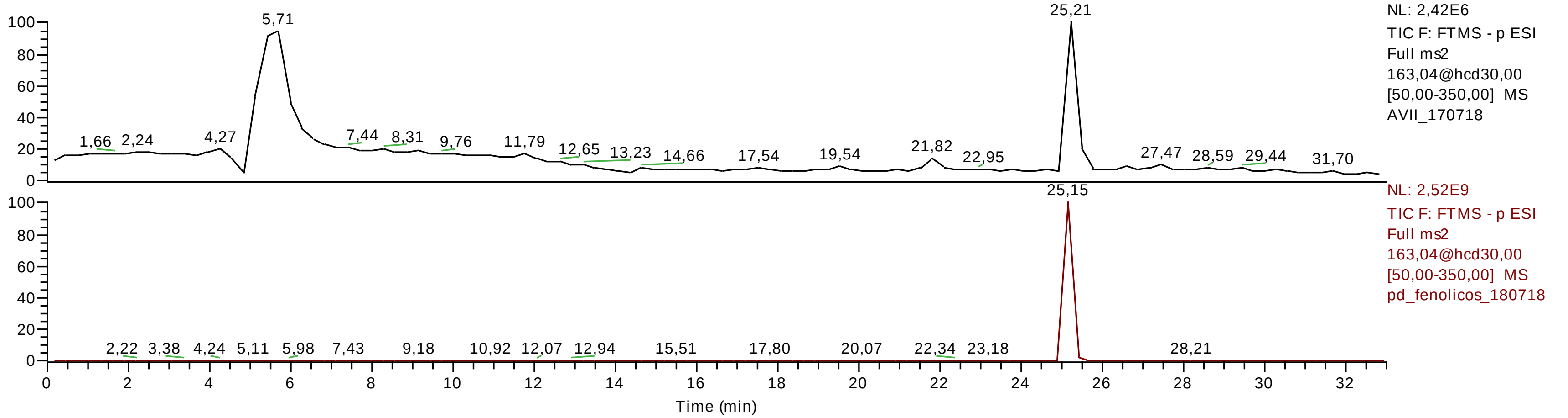

AVII_170718 #2457 RT: 25,21 AV: 1 NL: 1,68E6
F: FTMS - p ESI Full ms2 163,04@hcd30,00 [50,00-350,00]

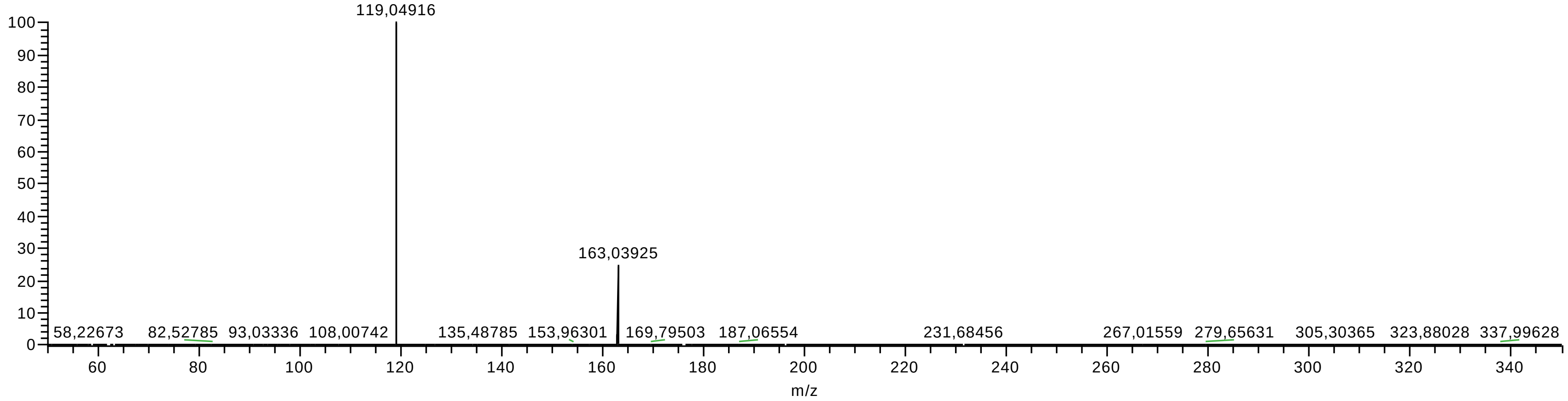

C:\Users\...\Claudiane\A' 17/07/2018 13:21:15

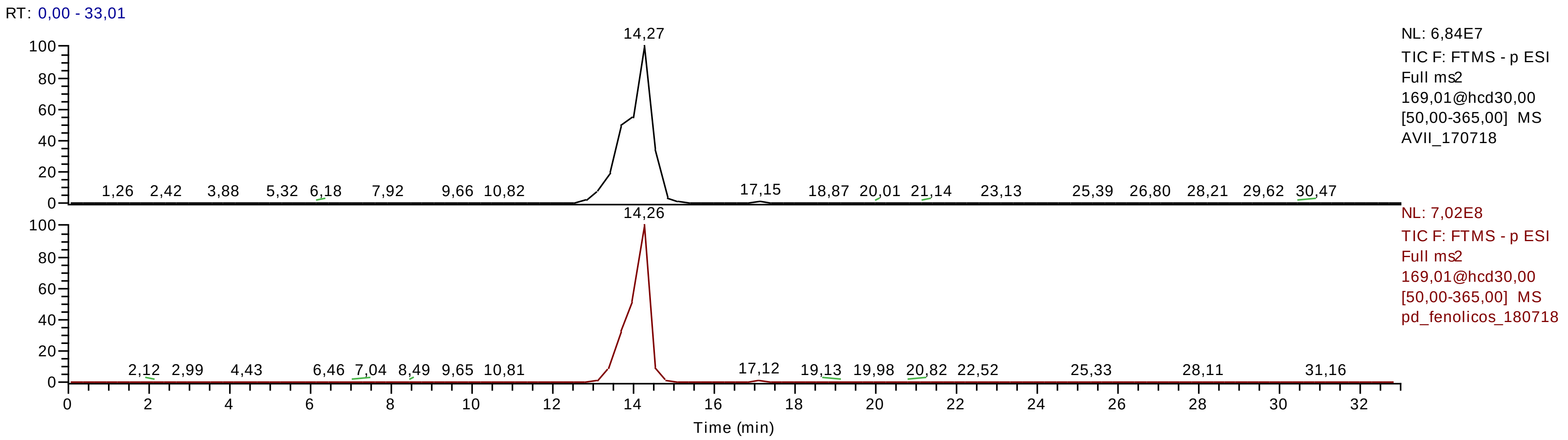

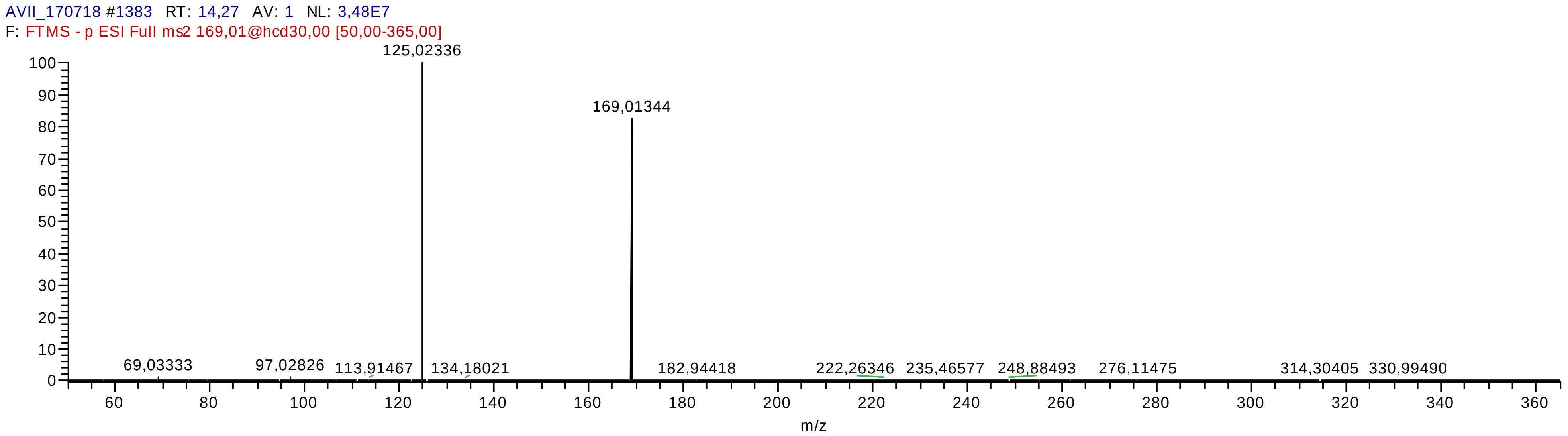

C:\Users\...\Claudiane\A\ 17/07/2018 13:21:15

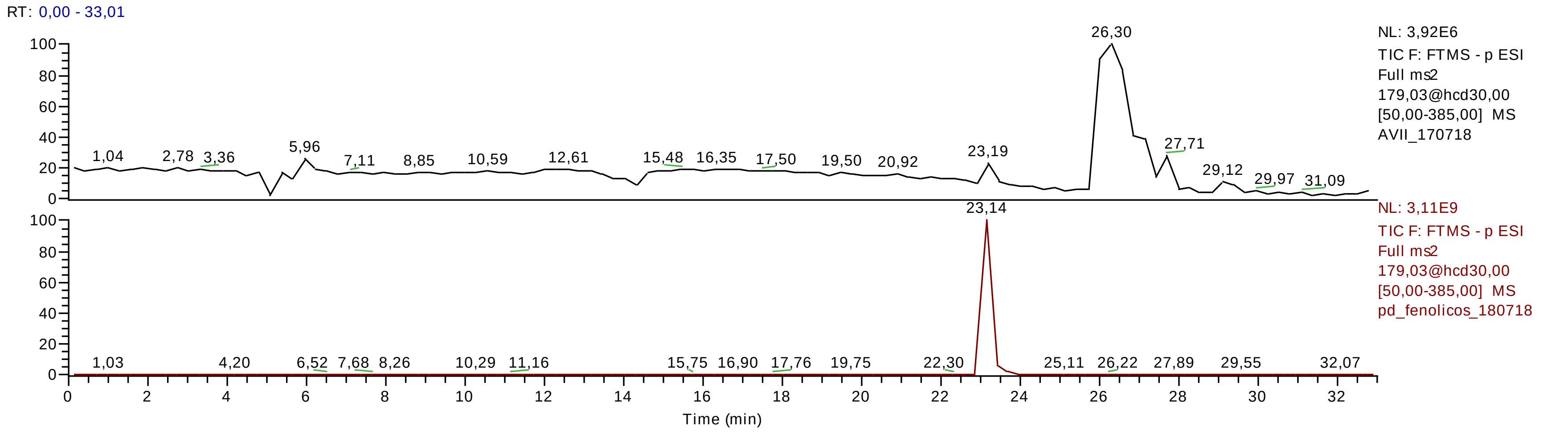

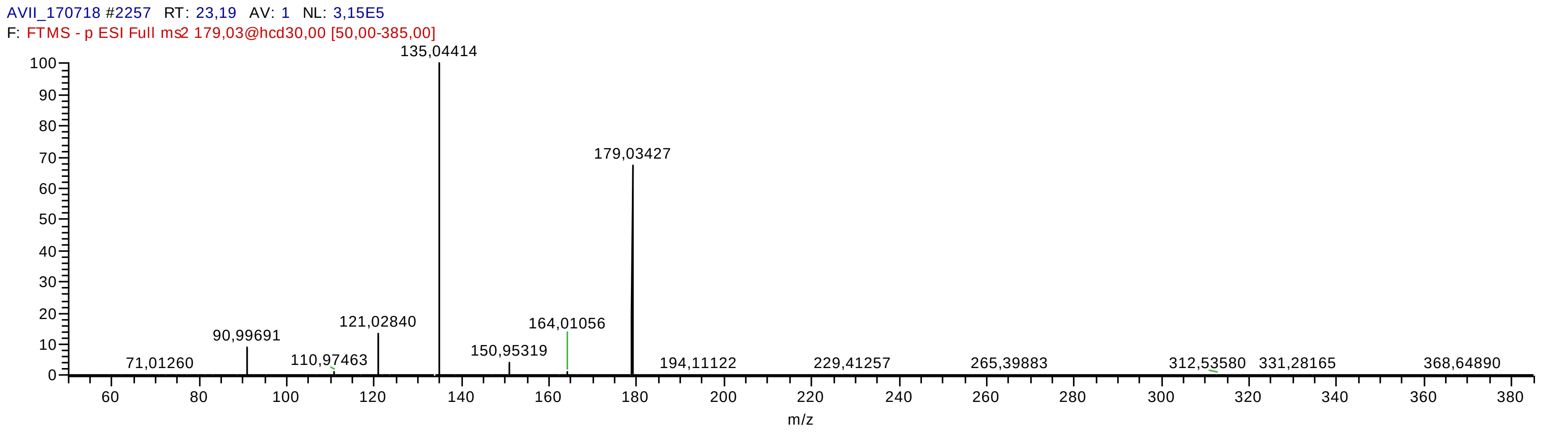

C:\Users\...\Claudiane\A\ 17/07/2018 13:21:15

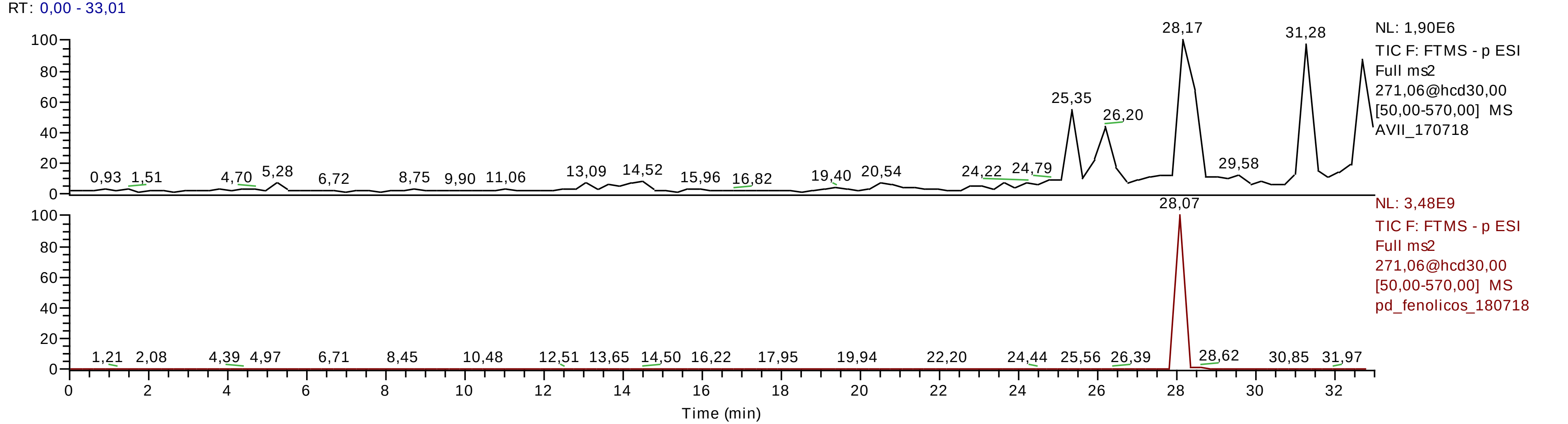

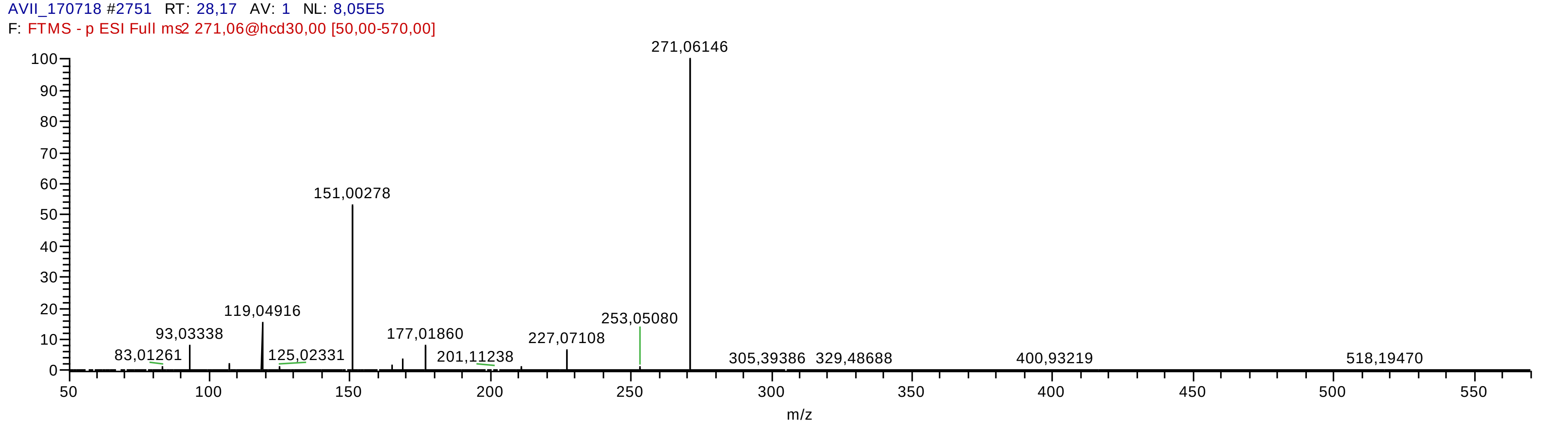

C:\Users\...\Claudiane\A\   17/07/2018 13:21:15

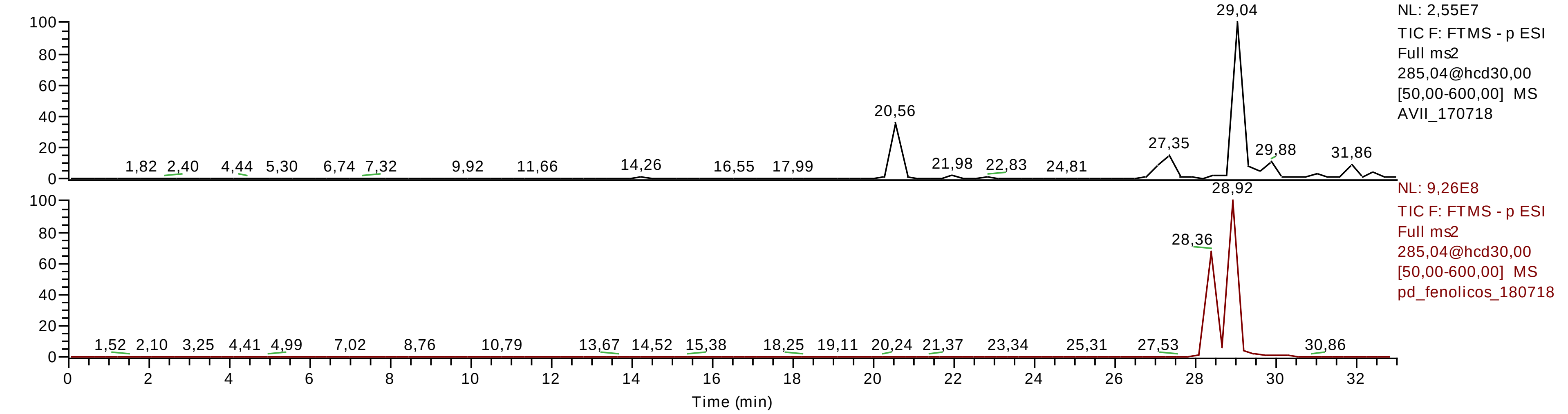

AVII_170718 #2837 RT: 29,04 AV: 1 NL: 2,31E7
F: FTMS - p ESI Full ms2 285,04@hcd30,00 [50,00-600,00]

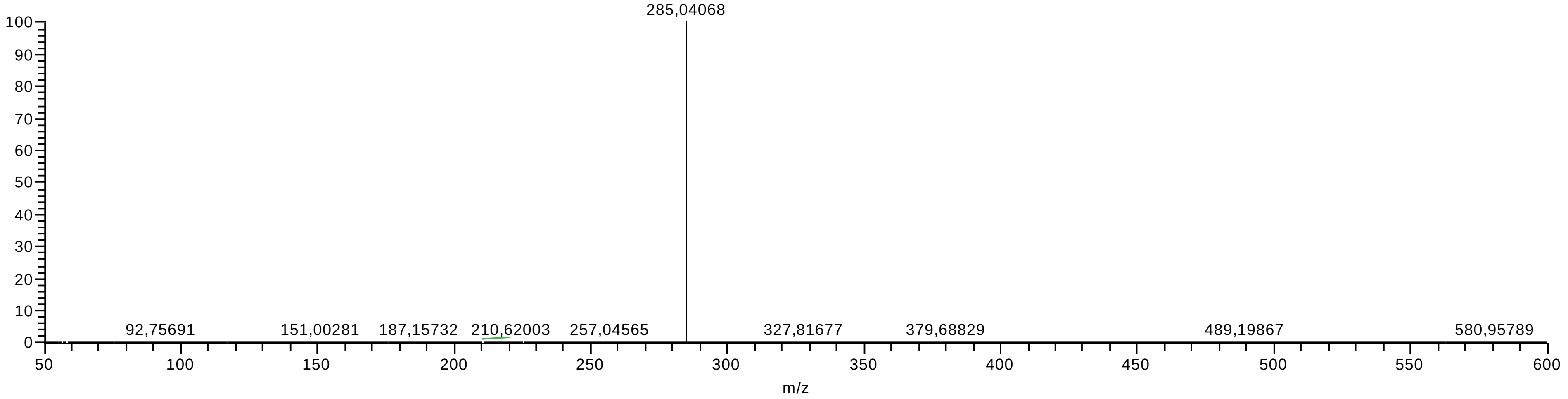

C:\Users\...\Claudiane\A\ 17/07/2018 13:21:15

RT: 0,00 - 33,01

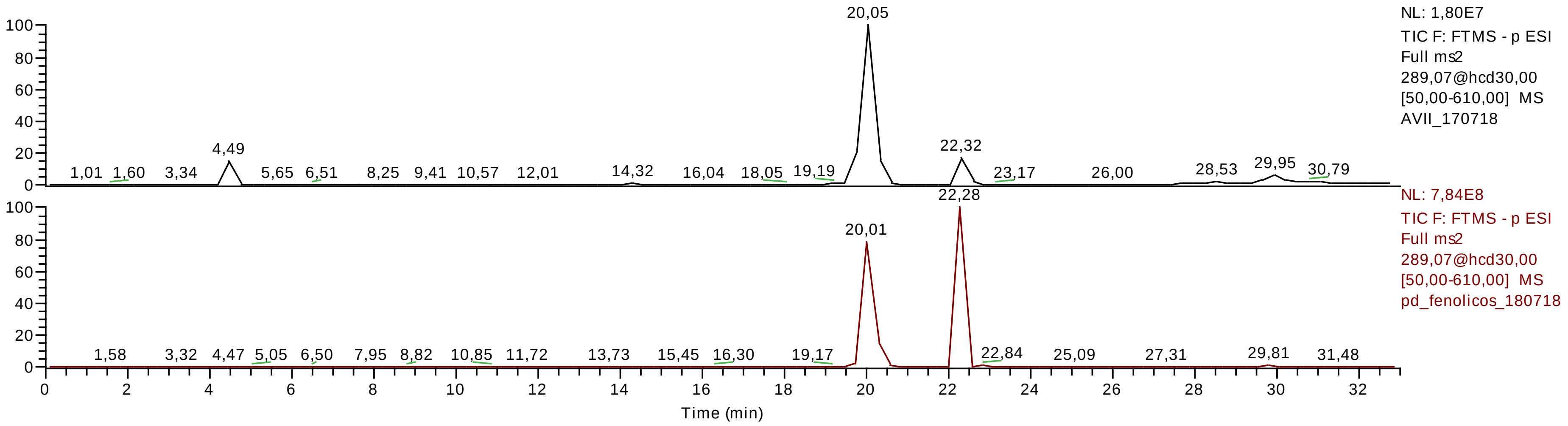

AVII_170718 #1947 RT: 20,05 AV: 1 NL: 5,55E6
F: FTMS - p ESI Full ms2 289,07@hcd30,00 [50,00-610,00]

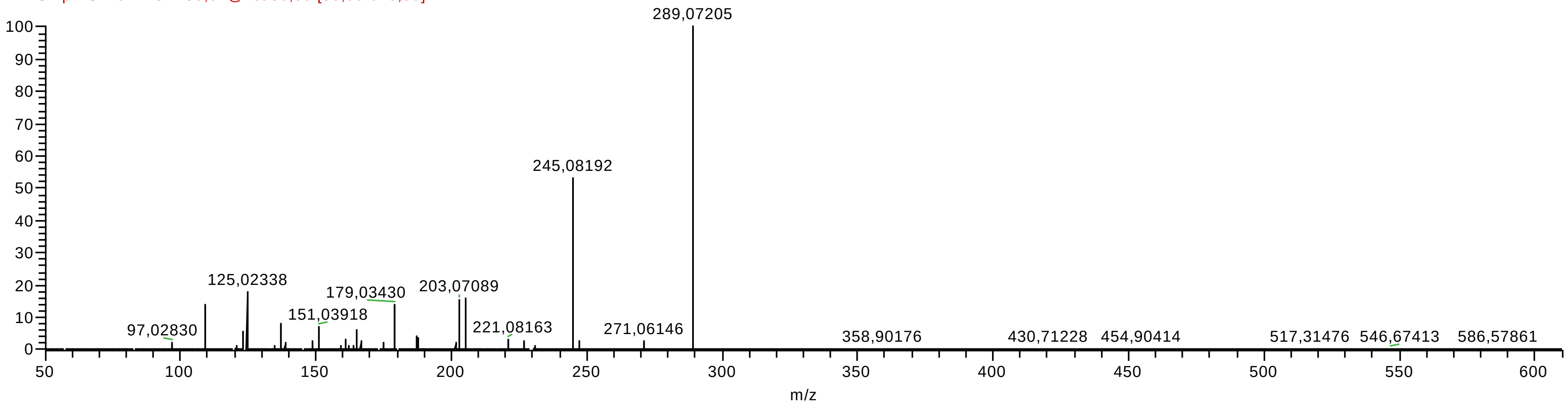

C:\Users\...\Claudiane\A\ 17/07/2018 13:21:15

RT: 0,00 - 33,01

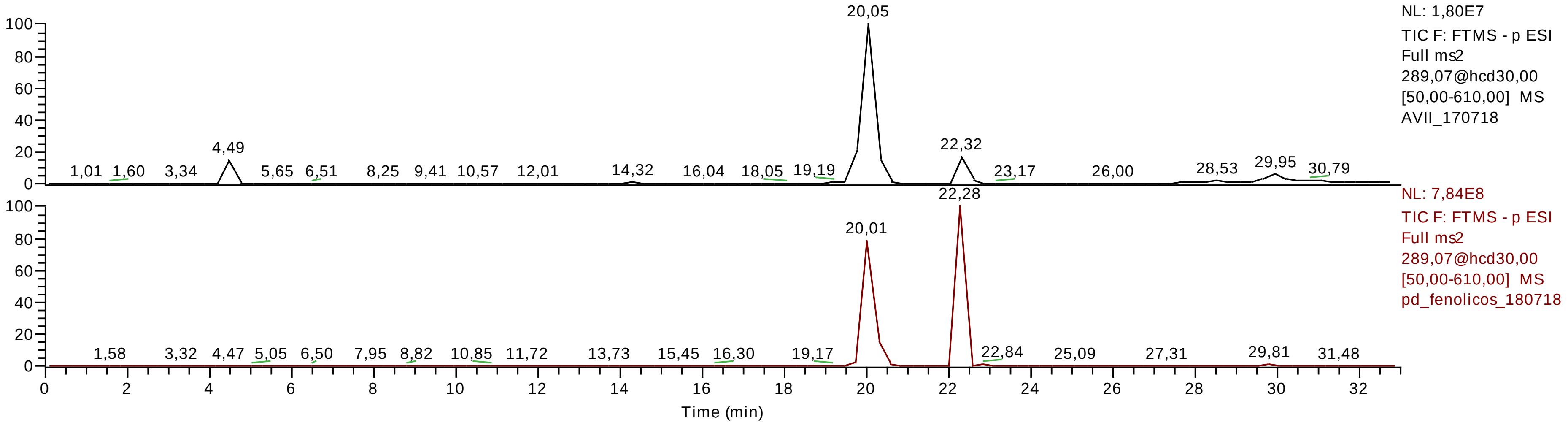

AVII_170718 #2171 RT: 22,32 AV: 1 NL: 9,52E5
F: FTMS - p ESI Full ms2 289,07@hcd30,00 [50,00-610,00]

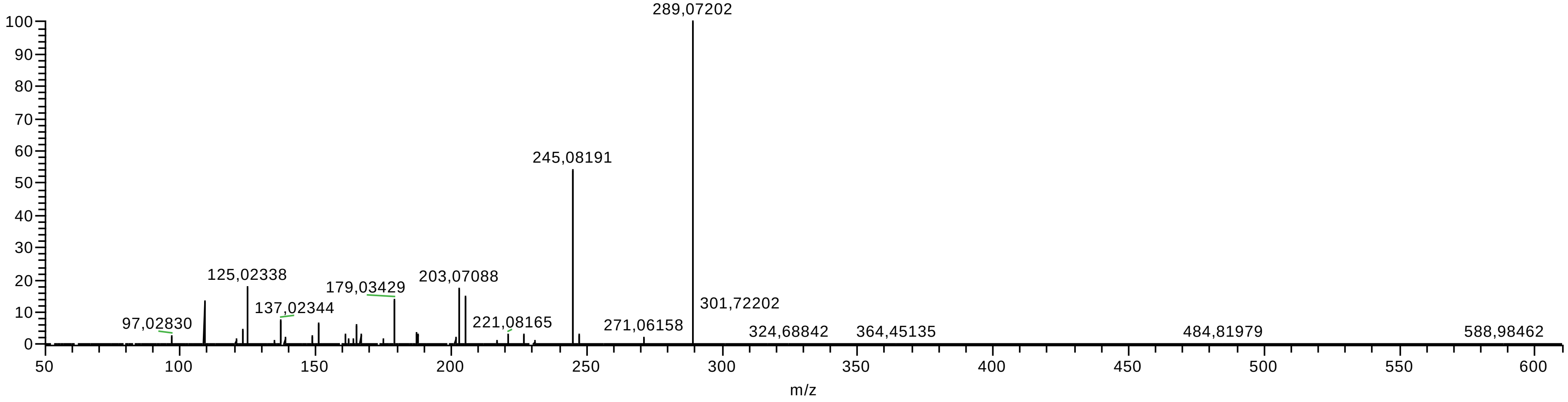

C:\Users\...\Claudiane\A\ 17/07/2018 13:21:15

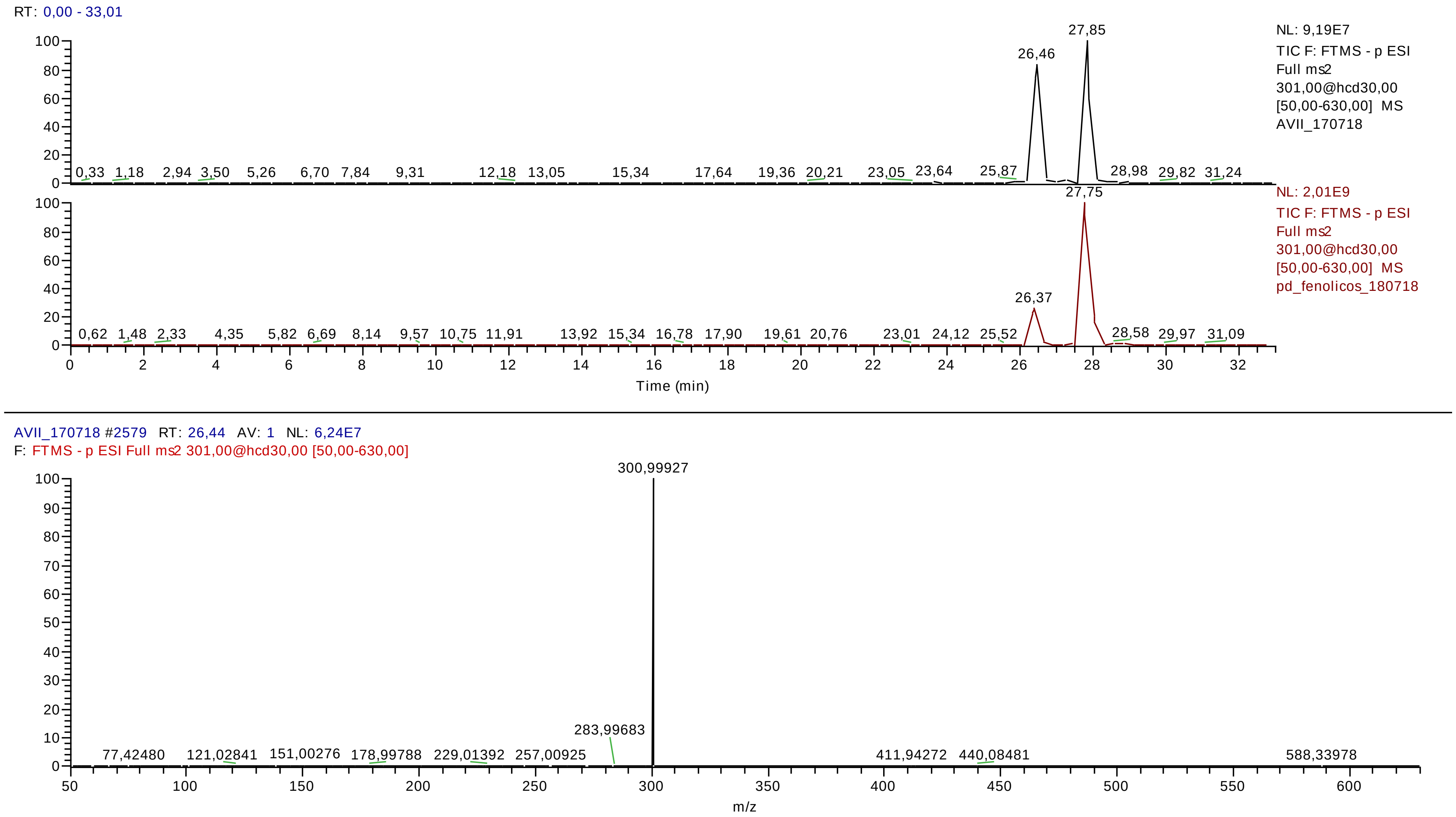

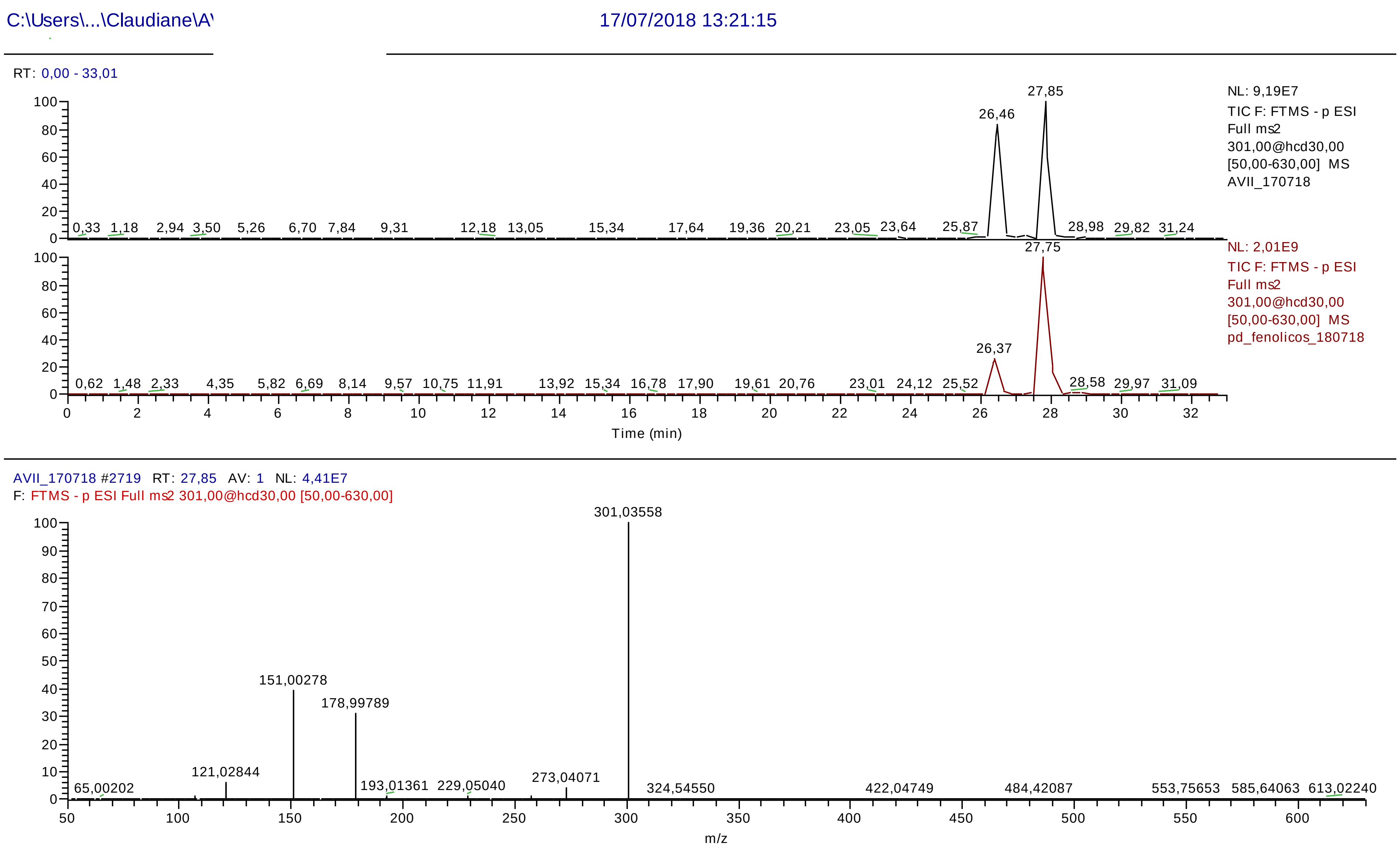
C:\Users\...\Claudiane\A\
17/07/2018 13:21:15
RT: 0,00 - 33,01
NL: 9,19E7
TIC F: FTMS - p ESI Full ms2 301,00@hcd30,00 [50,00-630,00] MS AVII_170718
27,85
26,46
NL: 2,01E9
TIC F: FTMS - p ESI Full ms2 301,00@hcd30,00 [50,00-630,00] MS pd_fenolicos_180718
27,75
26,37
Time (min)
AVII_170718 #2719 RT: 27,85 AV: 1 NL: 4,41E7
F: FTMS - p ESI Full ms2 301,00@hcd30,00 [50,00-630,00]
301,03558
151,00278
178,99789
121,02844
273,04071
65,00202
193,01361
229,05040
324,54550
422,04749
484,42087
553,75653
585,64063
613,02240
m/z

C:\Users\...\Claudiane\A' 17/07/2018 13:21:15

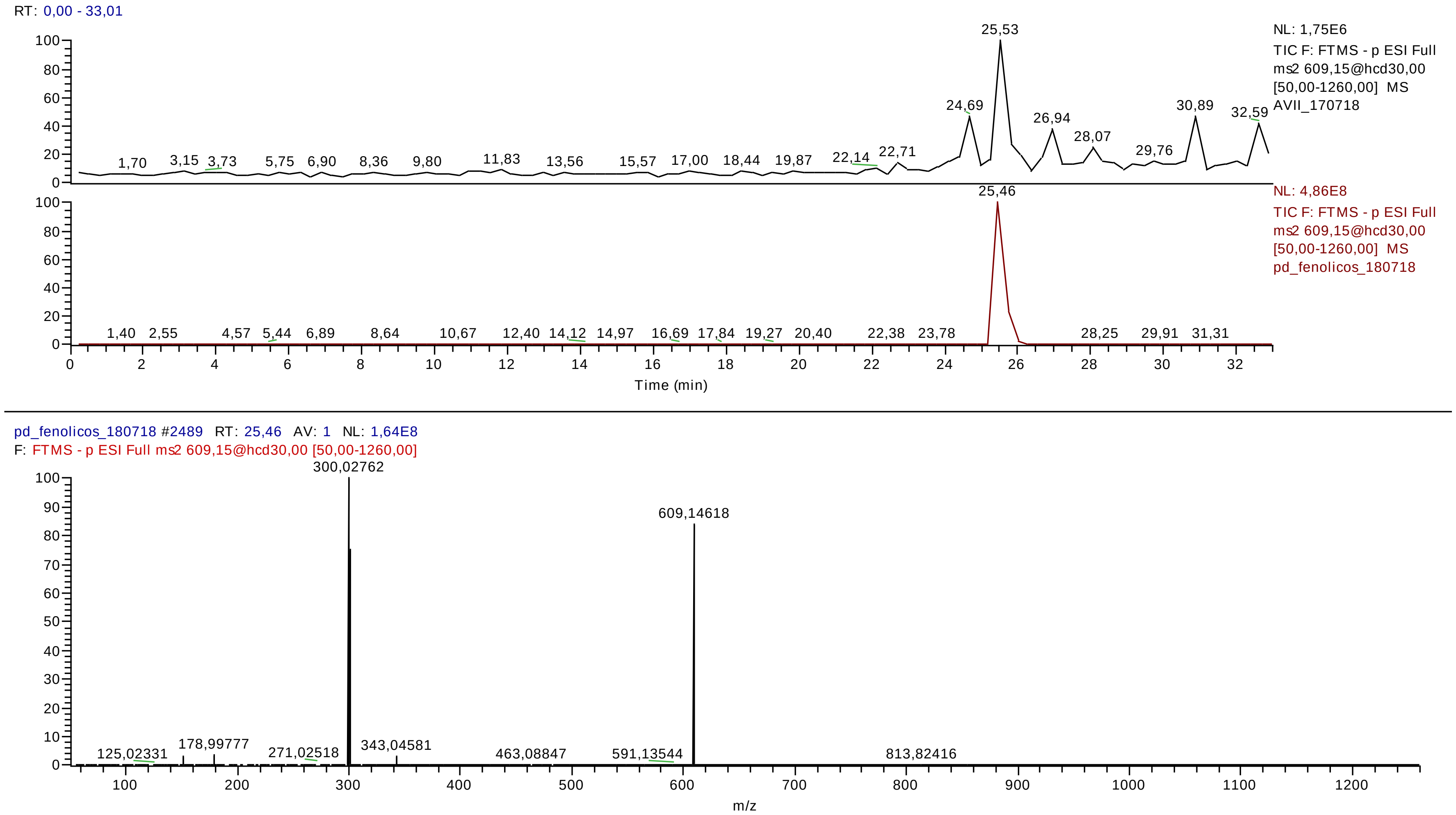

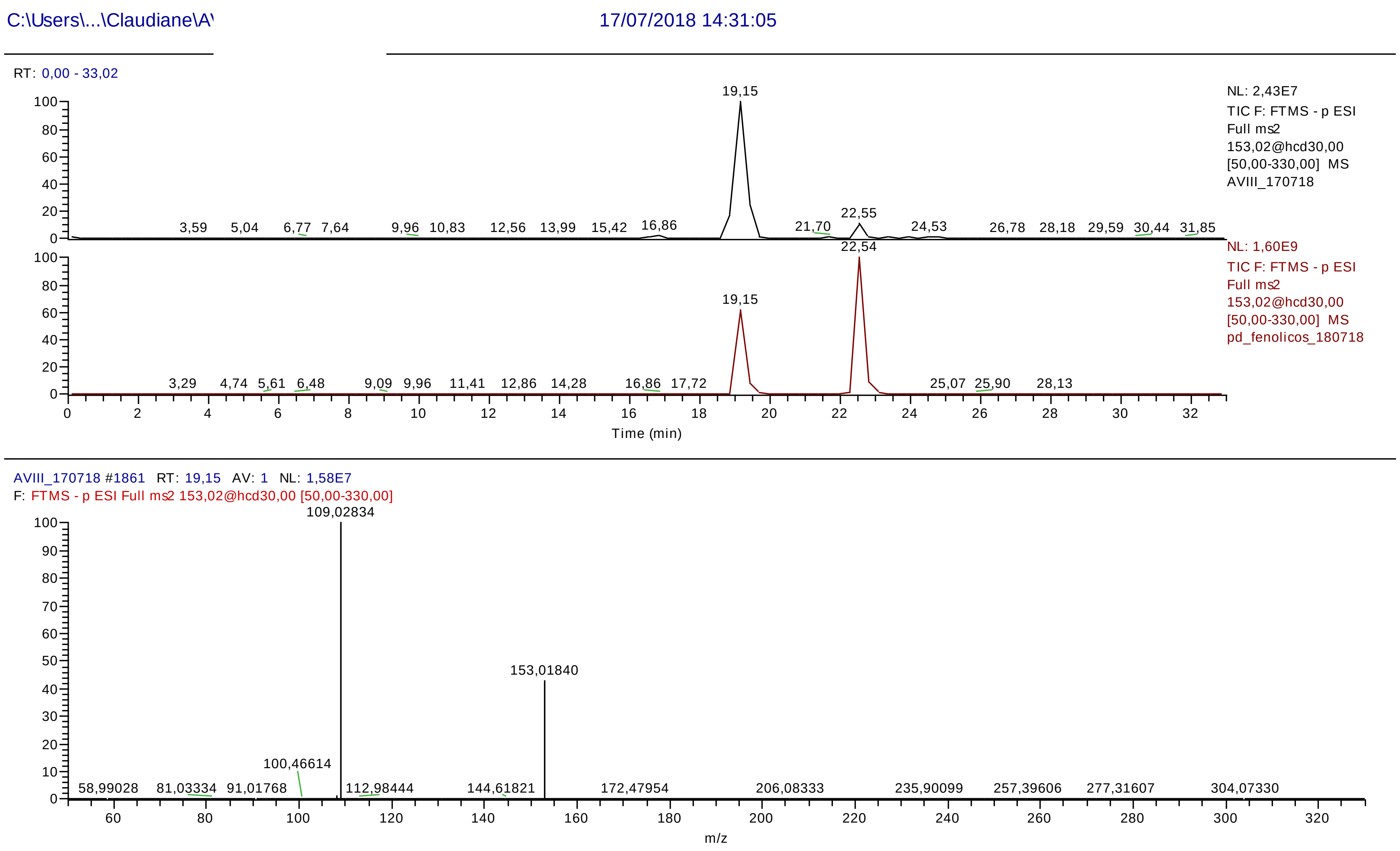
C:\Users\...\Claudiane\A'
17/07/2018 14:31:05
RT: 0,00 - 33,02
NL: 2,43E7
TIC F: FTMS - p ESI Full ms2 153,02@hcd30,00 [50,00-330,00] MS AVIII_170718
19,15
22,55
NL: 1,60E9
TIC F: FTMS - p ESI Full ms2 153,02@hcd30,00 [50,00-330,00] MS pd_fenolicos_180718
22,54
19,15
Time (min)
AVIII_170718 #1861 RT: 19,15 AV: 1 NL: 1,58E7
F: FTMS - p ESI Full ms2 153,02@hcd30,00 [50,00-330,00]
109,02834
153,01840
100,46614
58,99028
81,03334
91,01768
112,98444
144,61821
172,47954
206,08333
235,90099
257,39606
277,31607
304,07330
m/z

C:\Users\...\Claudiane\A\

17/07/2018 14:31:05

RT: 0,00 - 33,02

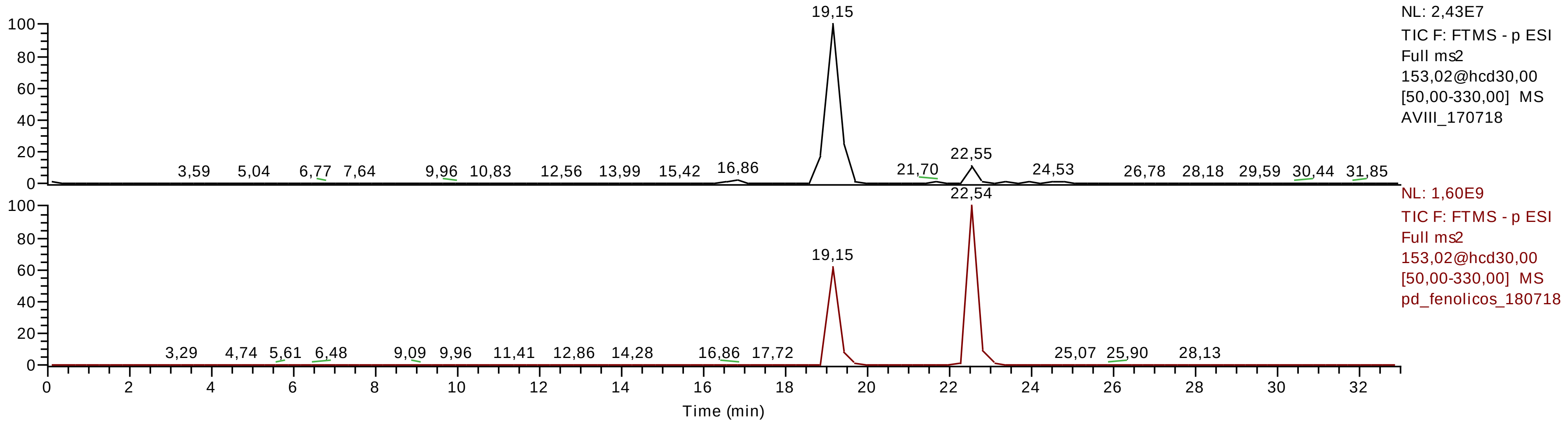

AVIII_170718 #2197 RT: 22,55 AV: 1 NL: 9,53E5
F: FTMS - p ESI Full ms2 153,02@hcd30,00 [50,00-330,00]

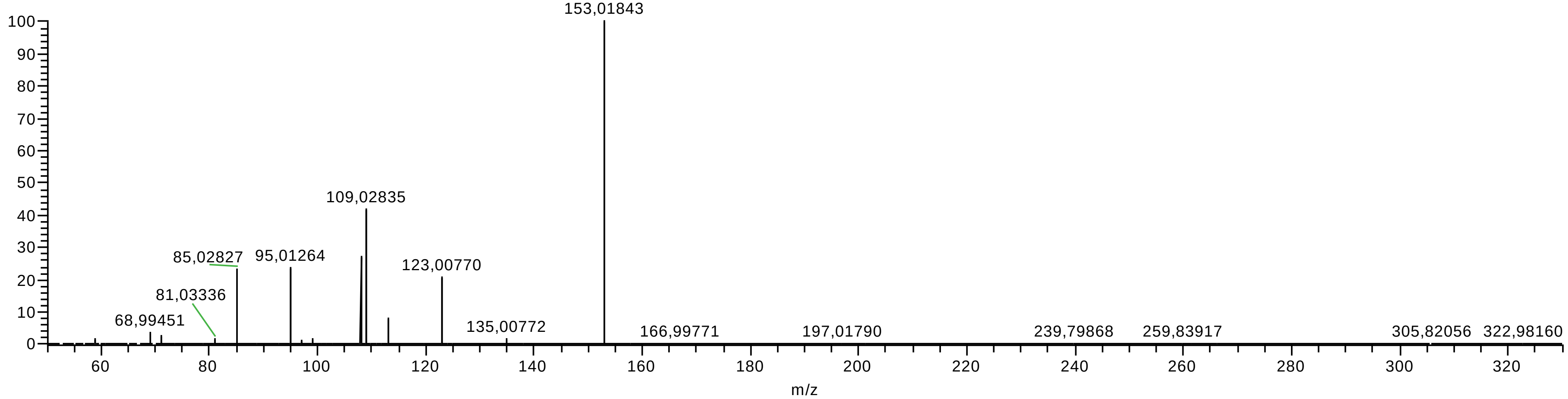

C:\Users\...\Claudiane\A\ 17/07/2018 14:31:05

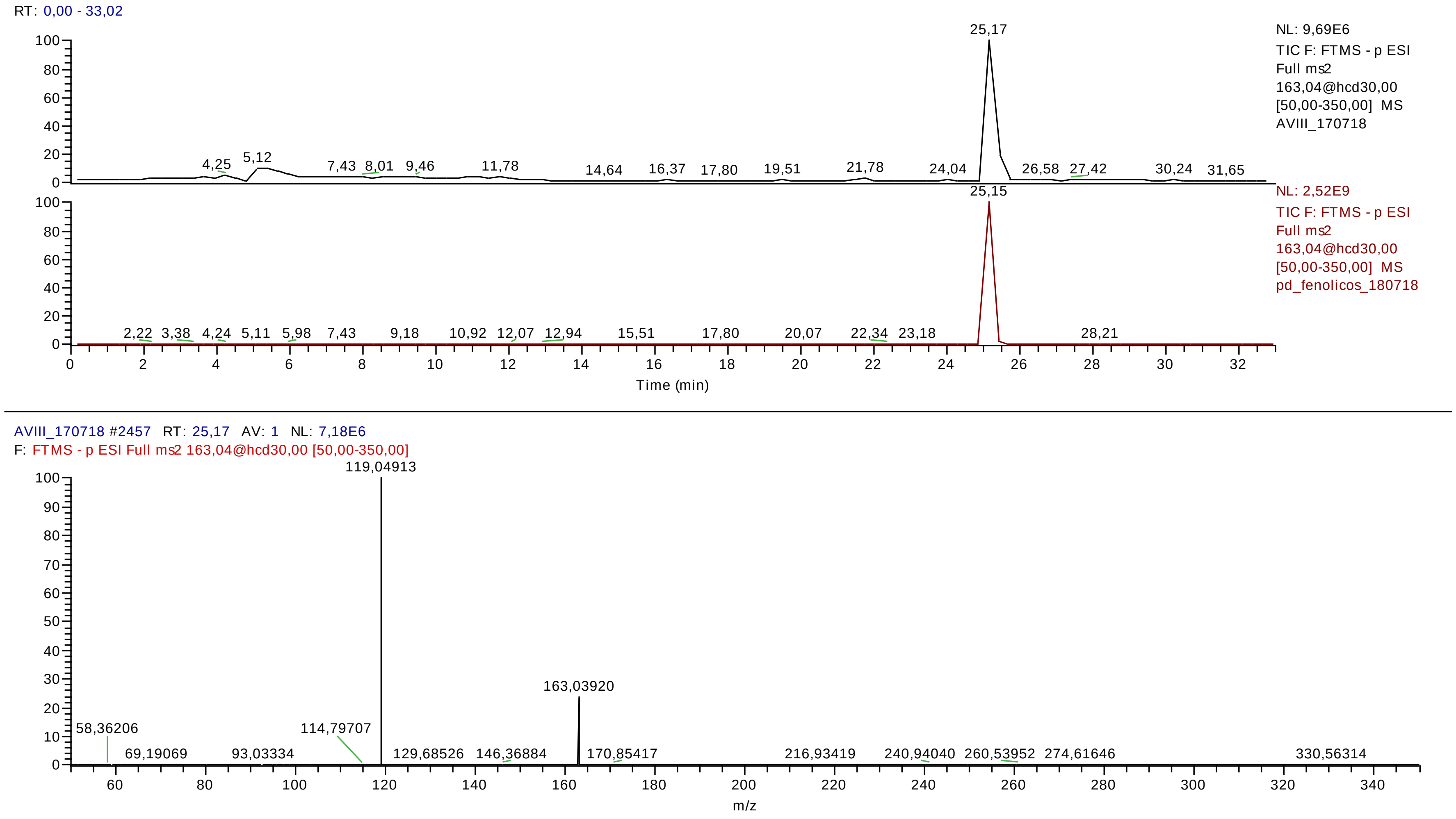

C:\Users\...\Claudiane\A\ 17/07/2018 14:31:05

RT: 0,00 - 33,02

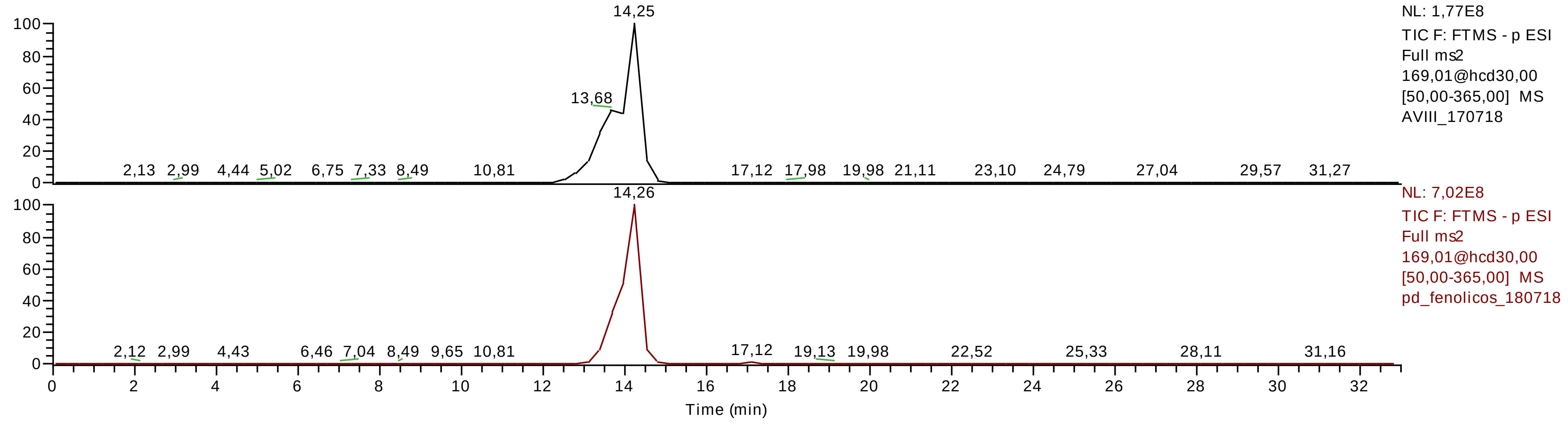

AVIII_170718 #1383 RT: 14,25 AV: 1 NL: 8,85E7
F: FTMS - p ESI Full ms2 169,01@hcd30,00 [50,00-365,00]

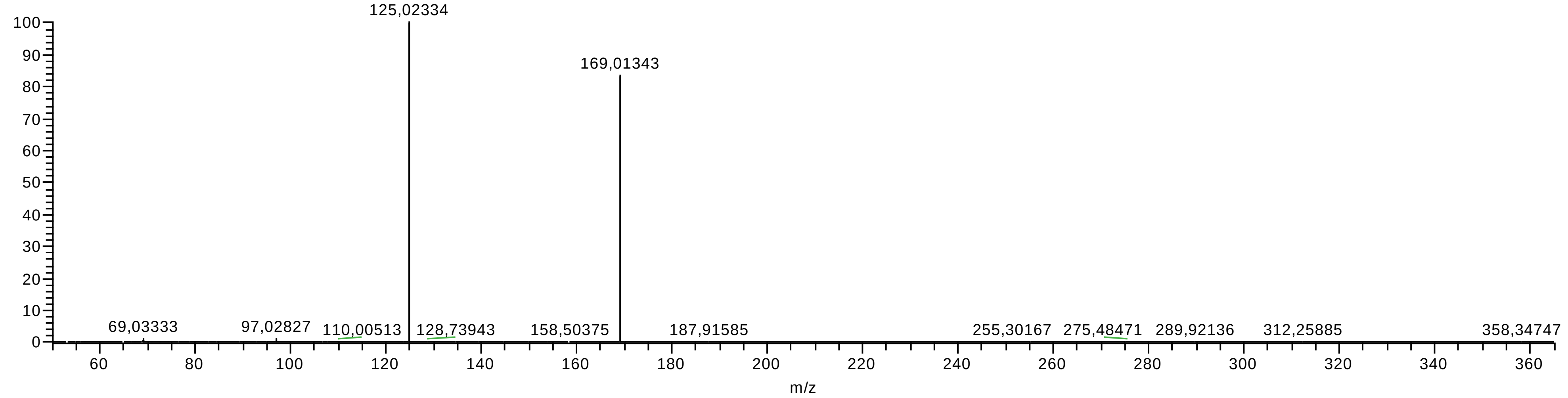

C:\Users\...\Claudiane\A\ 17/07/2018 14:31:05

RT: 0,00 - 33,02

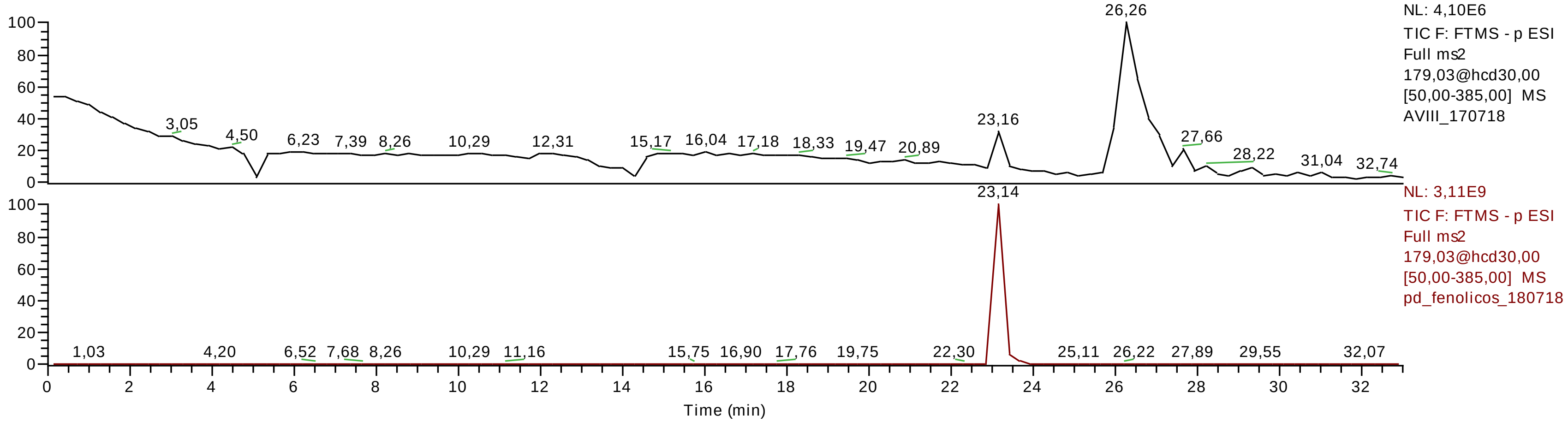

AVIII_170718 #2257 RT: 23,16 AV: 1 NL: 5,79E5
F: FTMS - p ESI Full ms2 179,03@hcd30,00 [50,00-385,00]

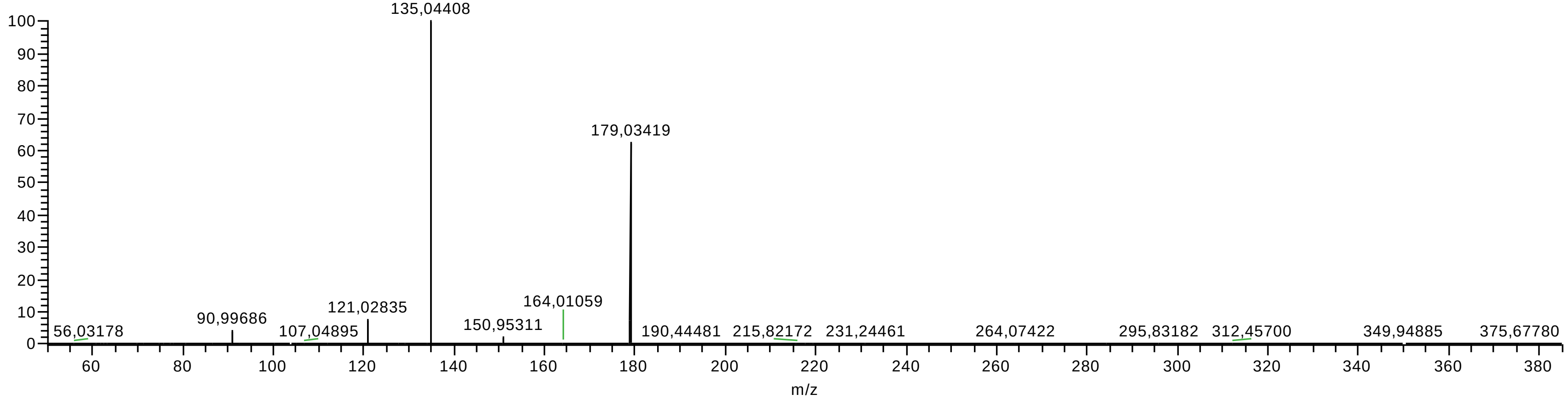

C:\Users\...\Claudiane\A\ 17/07/2018 14:31:05

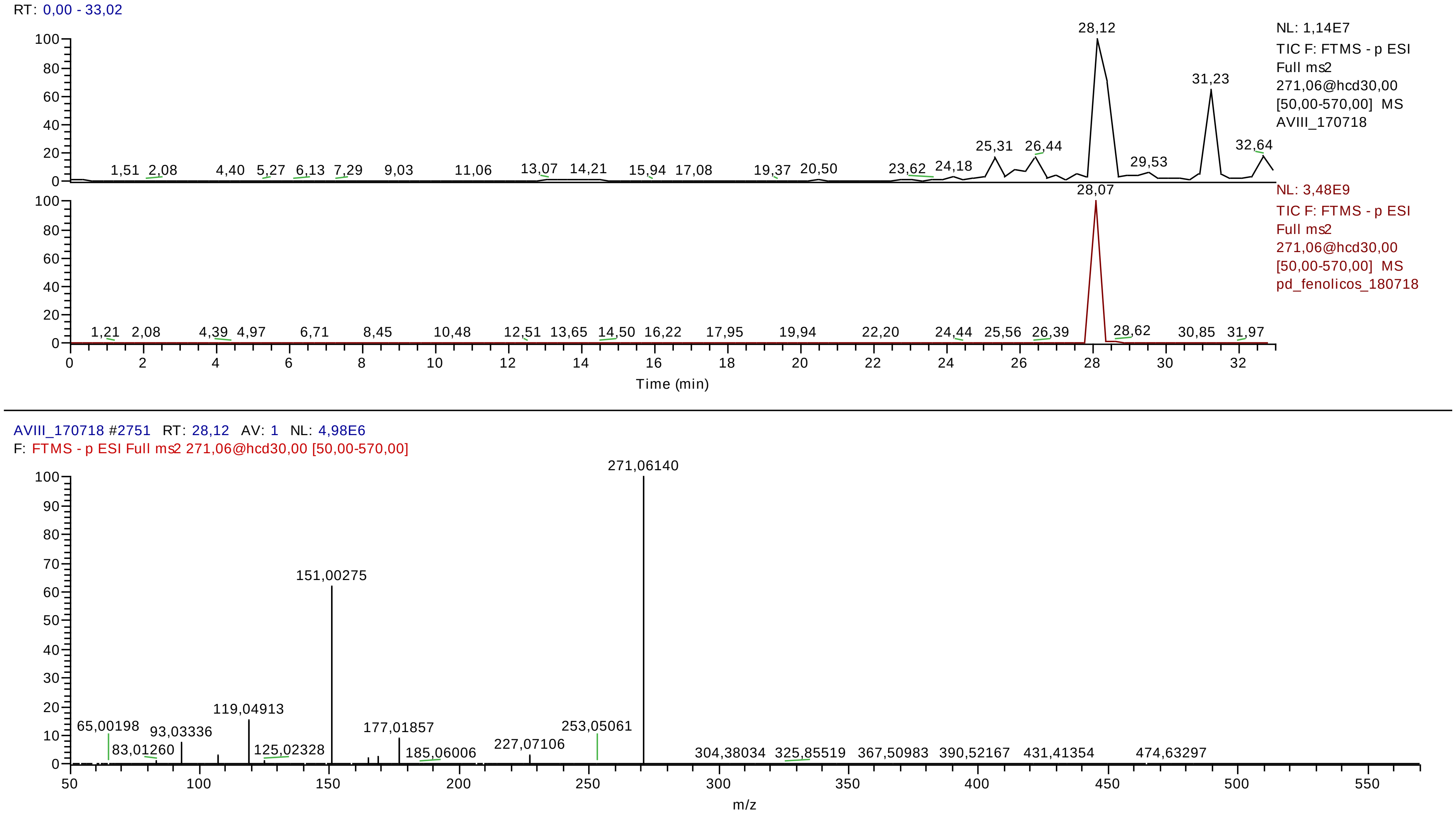

C:\Users\...\Claudiane\A\ 17/07/2018 14:31:05

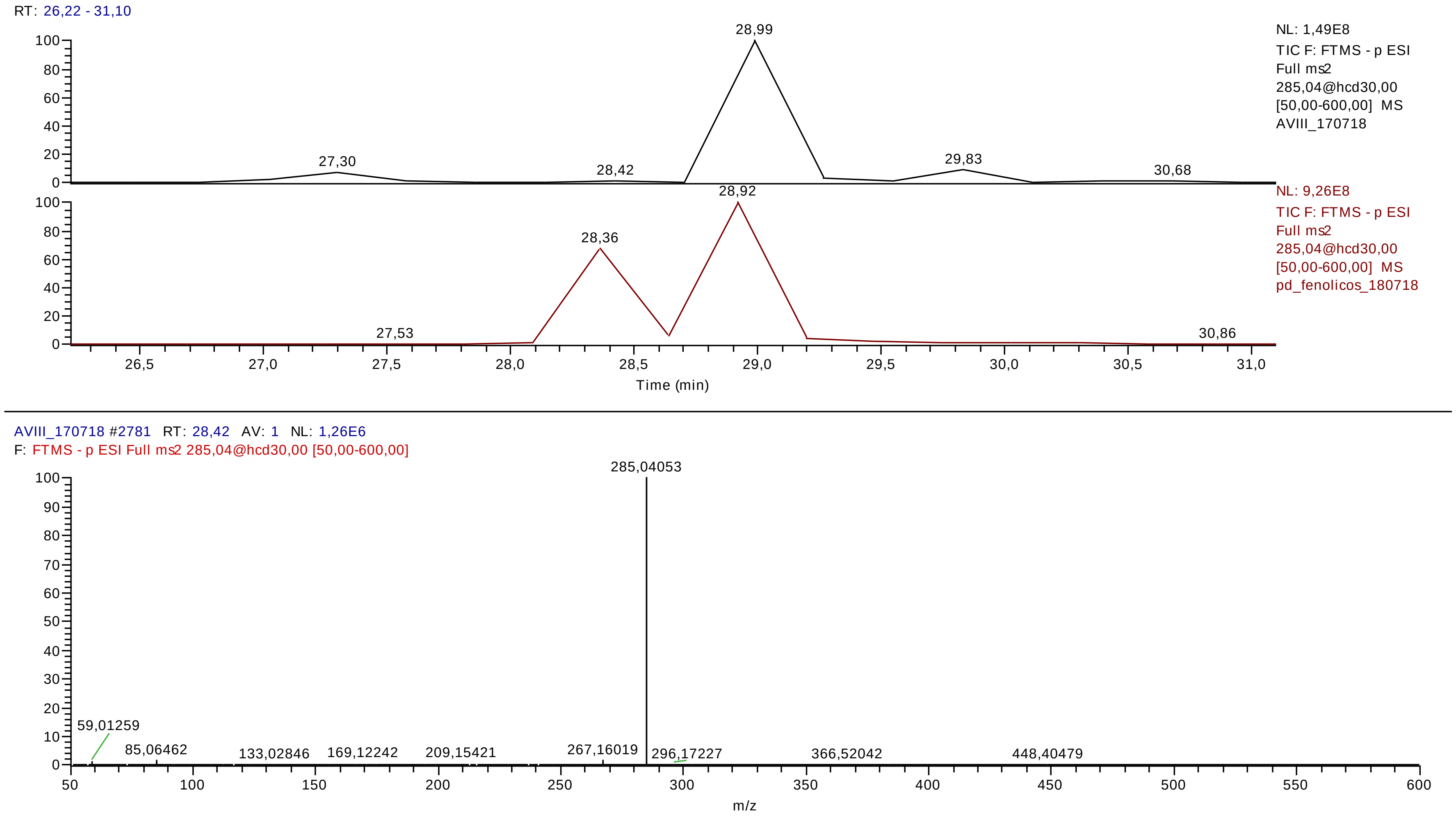

C:\Users\...\Claudiane\A\ 17/07/2018 14:31:05

RT: 26,22 - 31,10

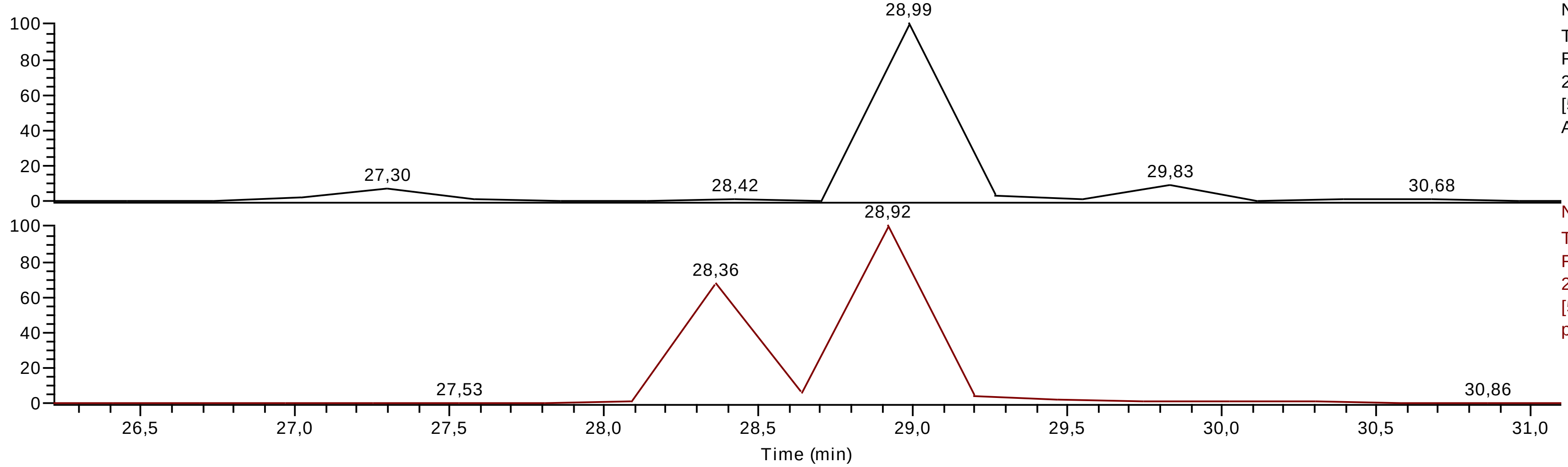

AVIII_170718 #2837 RT: 28,99 AV: 1 NL: 1,37E8
F: FTMS - p ESI Full ms2 285,04@hcd30,00 [50,00-600,00]

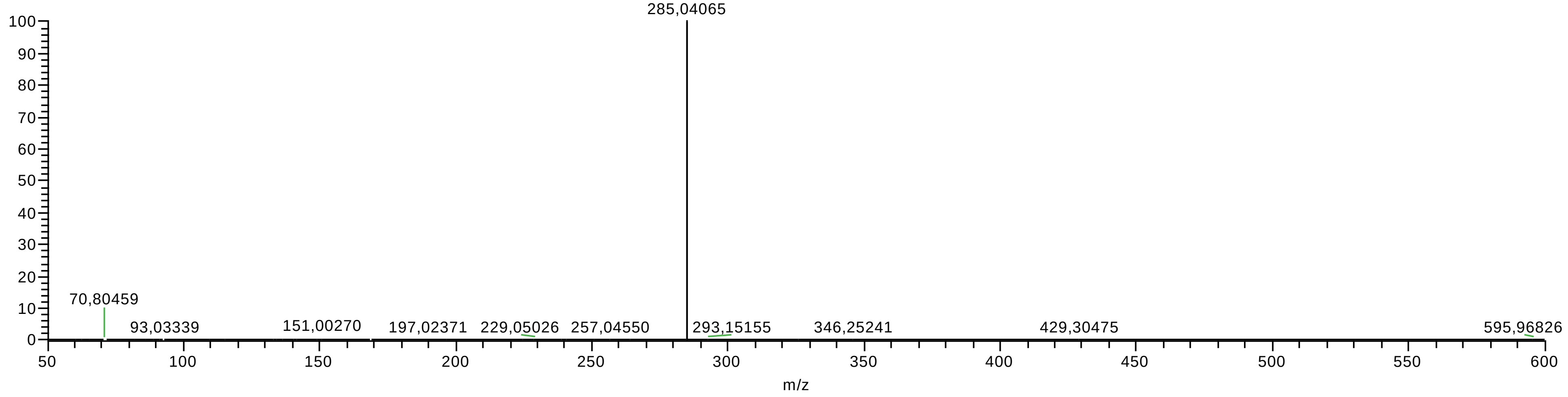

C:\Users\...\Claudiane\A\   17/07/2018 14:31:05

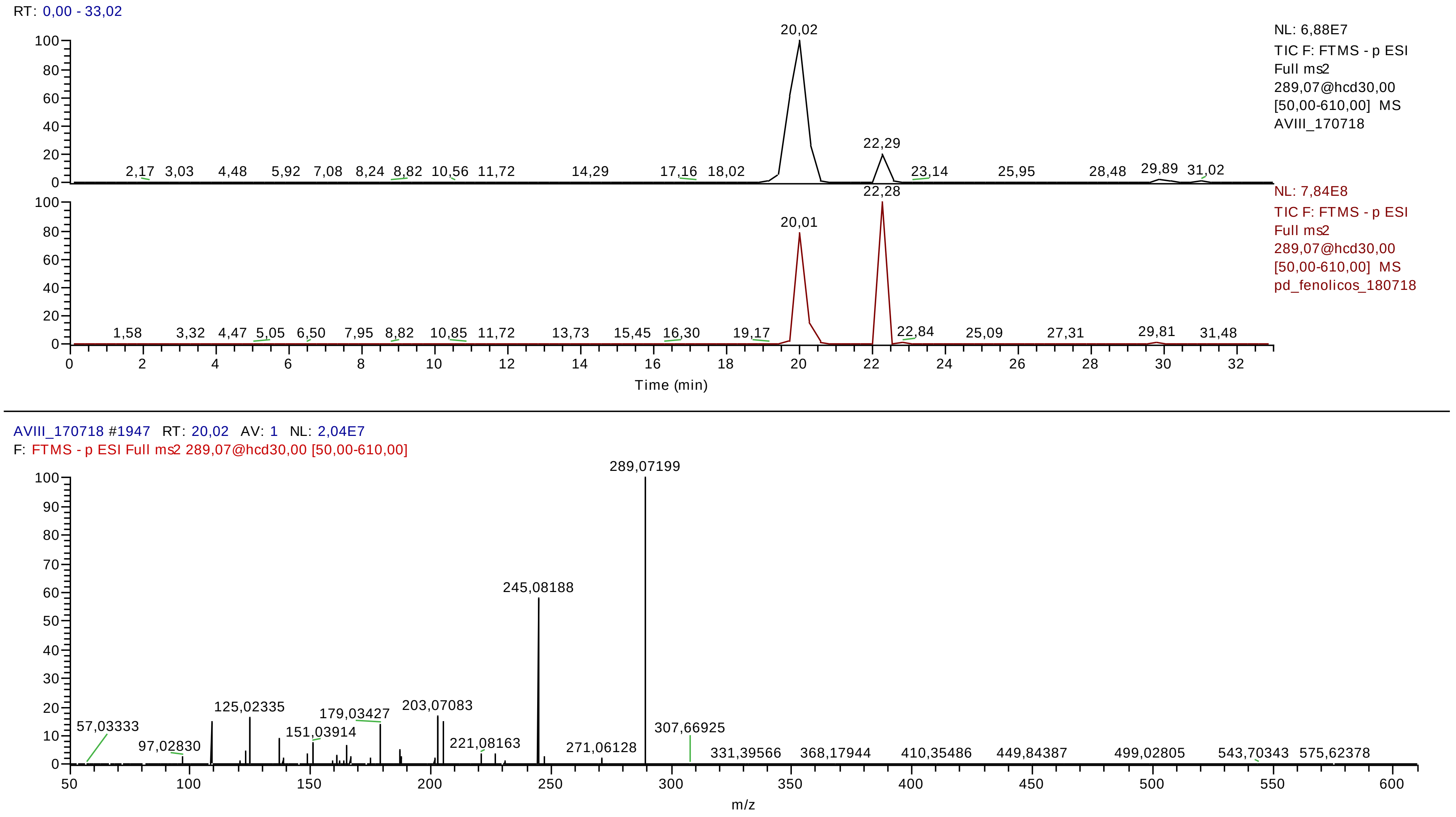

C:\Users\...\Claudiane\A\ 17/07/2018 14:31:05

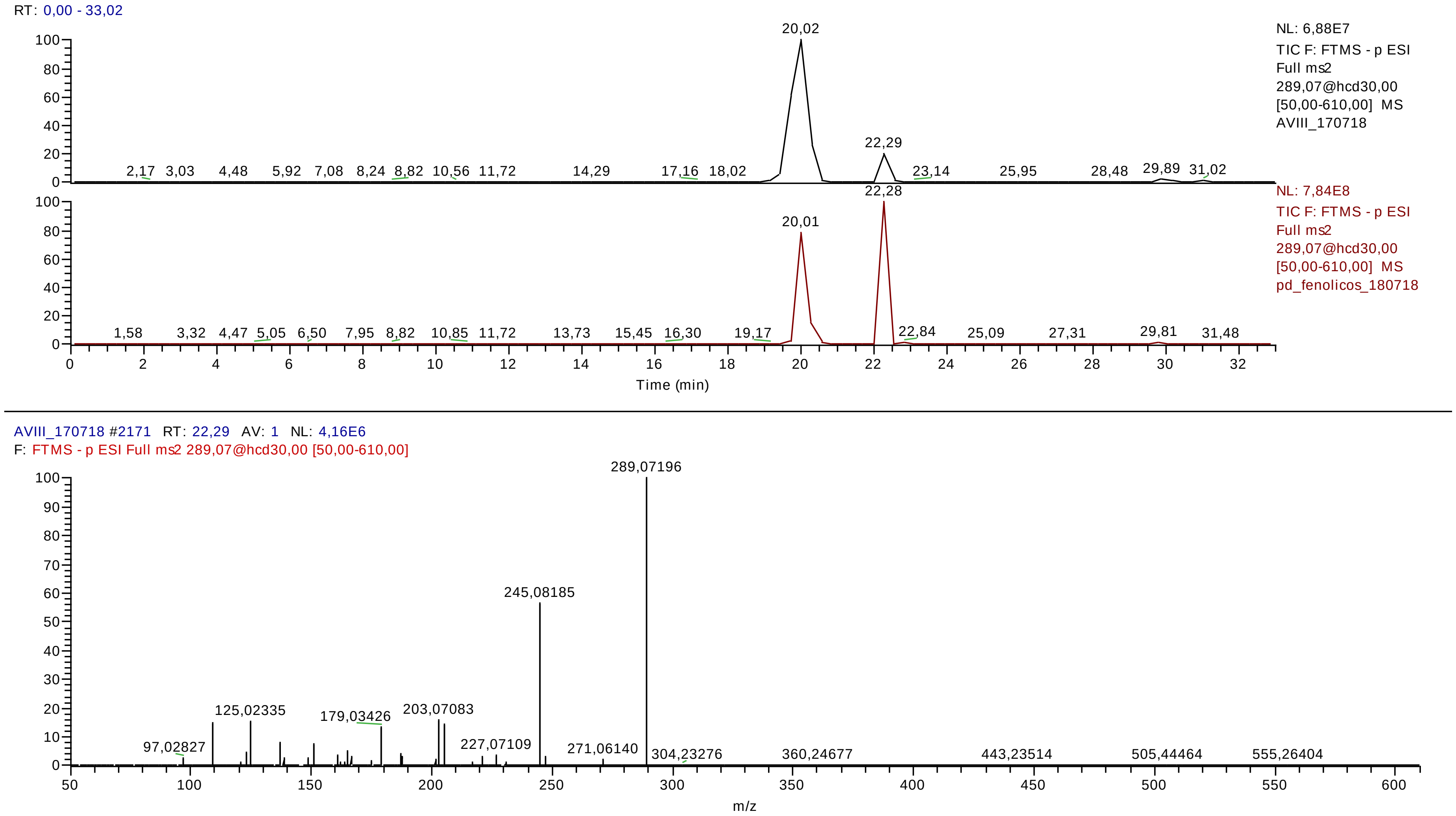

C:\Users\...\Claudiane\A\   17/07/2018 14:31:05

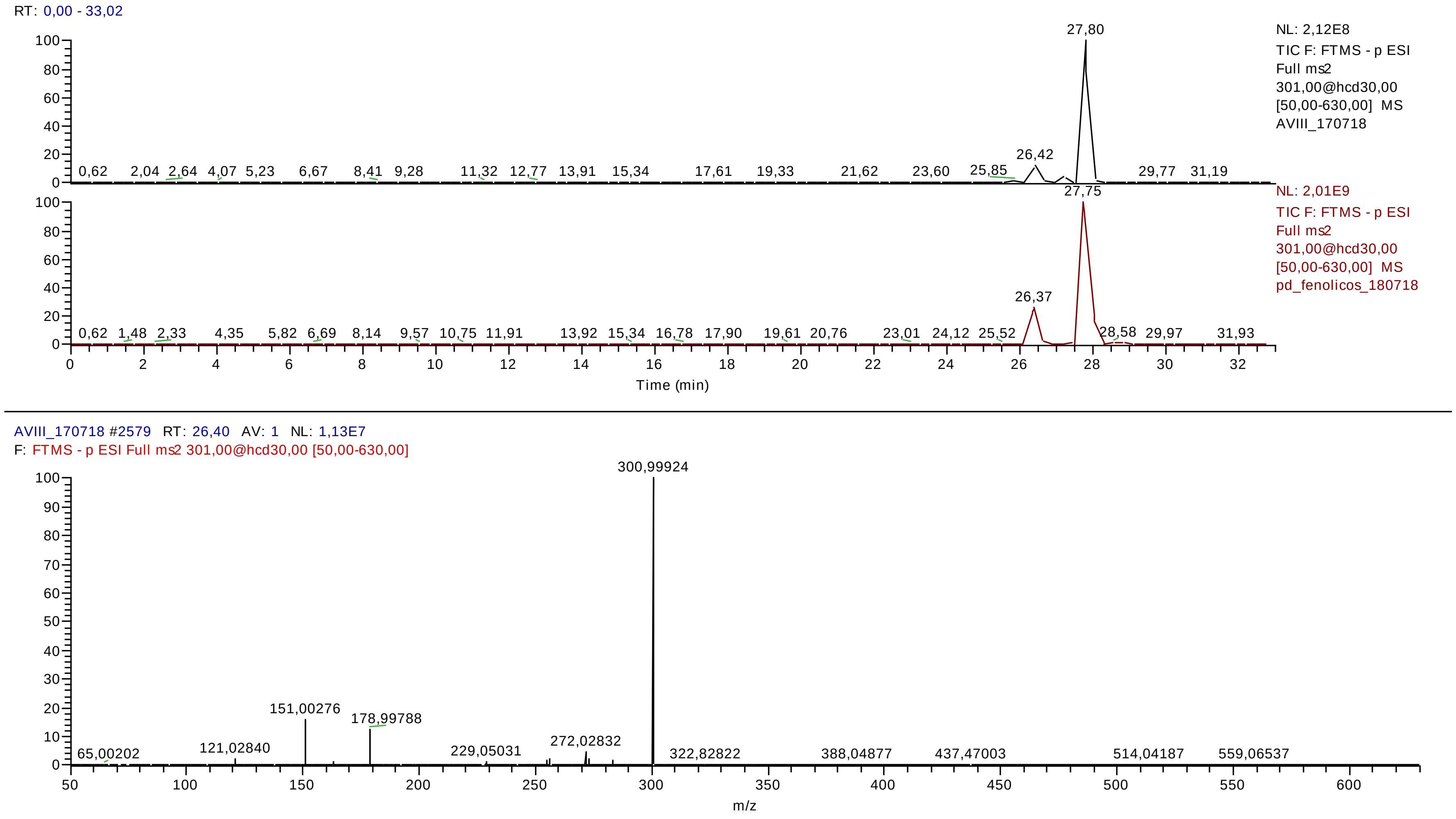

C:\Users\...\Claudiane\A\ 17/07/2018 14:31:05

RT: 0,00 - 33,02

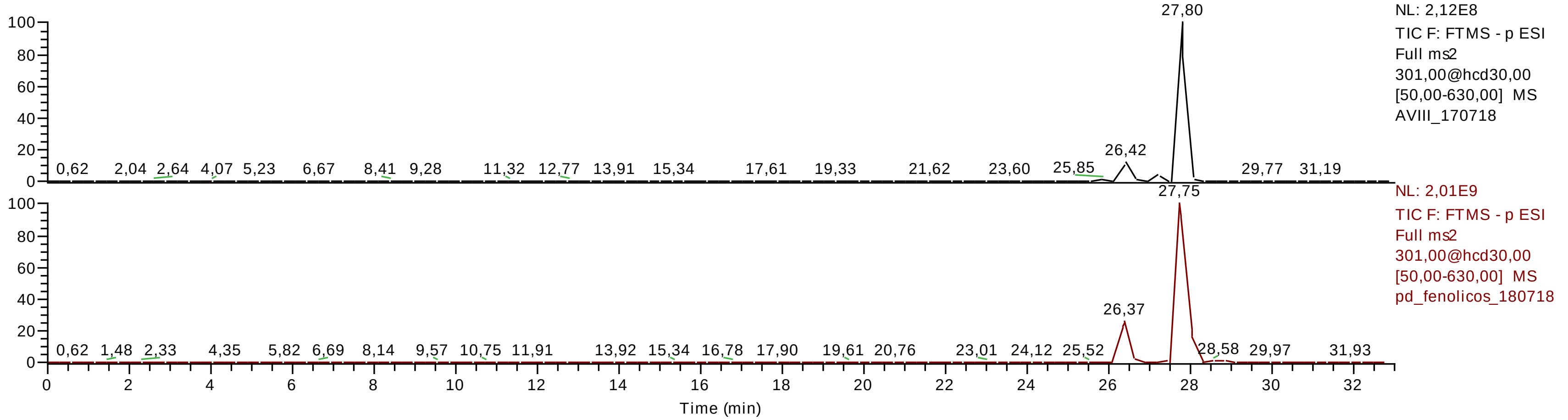

AVIII_170718 #2721 RT: 27,82 AV: 1 NL: 7,84E7

F: FTMS - p ESI Full ms2 301,00@hcd30,00 [50,00-630,00]

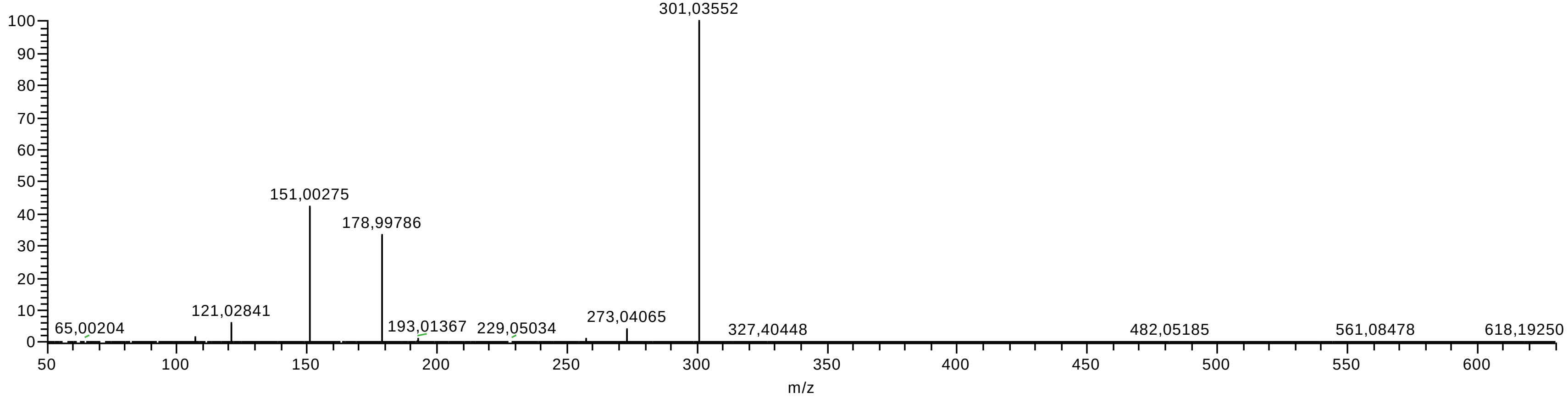

C:\Users\...\Claudian 17/07/2018 14:31:05

RT: 0,00 - 33,02

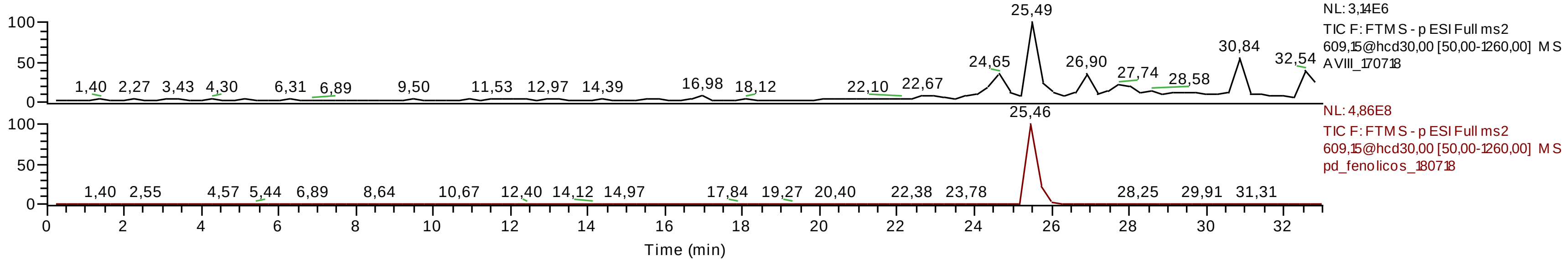

AVIII_170718 #2489 RT: 25,49 AV: 1 NL: 7,95E5
F: FTMS - p ESI Full ms2 609,15@hcd30,00 [50,00-1260,00]

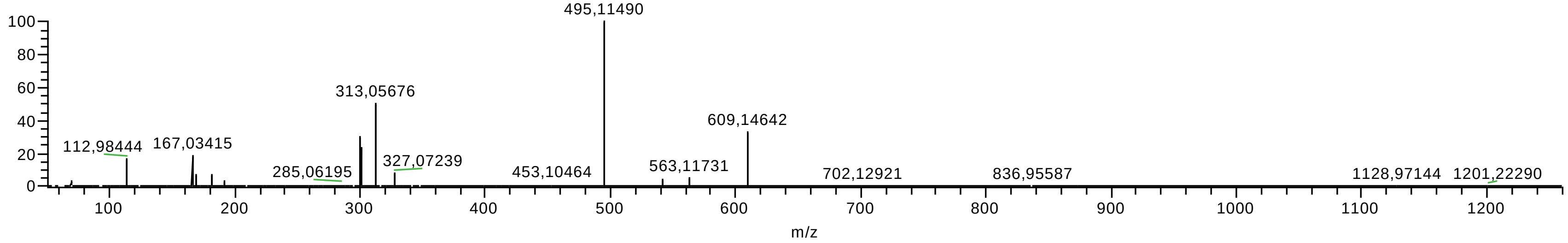

AVIII_170718 #2489 RT: 25,49 AV: 1 NL: 4,05E5
F: FTMS - p ESI Full ms2 609,15@hcd30,00 [50,00-1260,00]

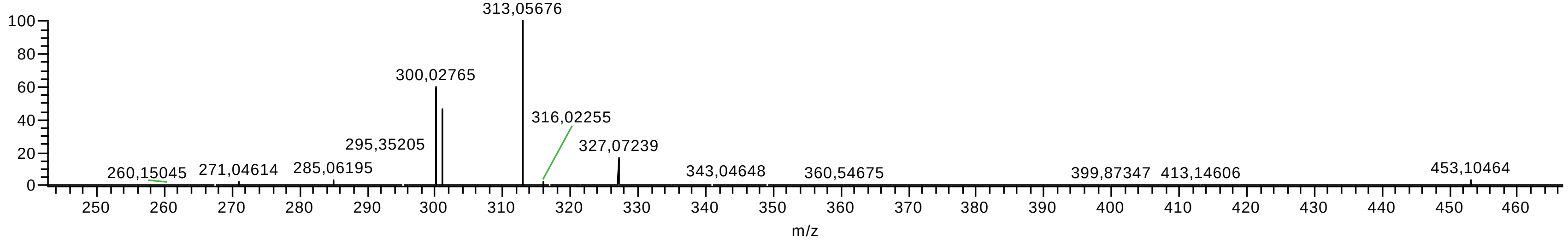

C:\Users\...\Claudiane\AI 17/07/2018 12:01:30

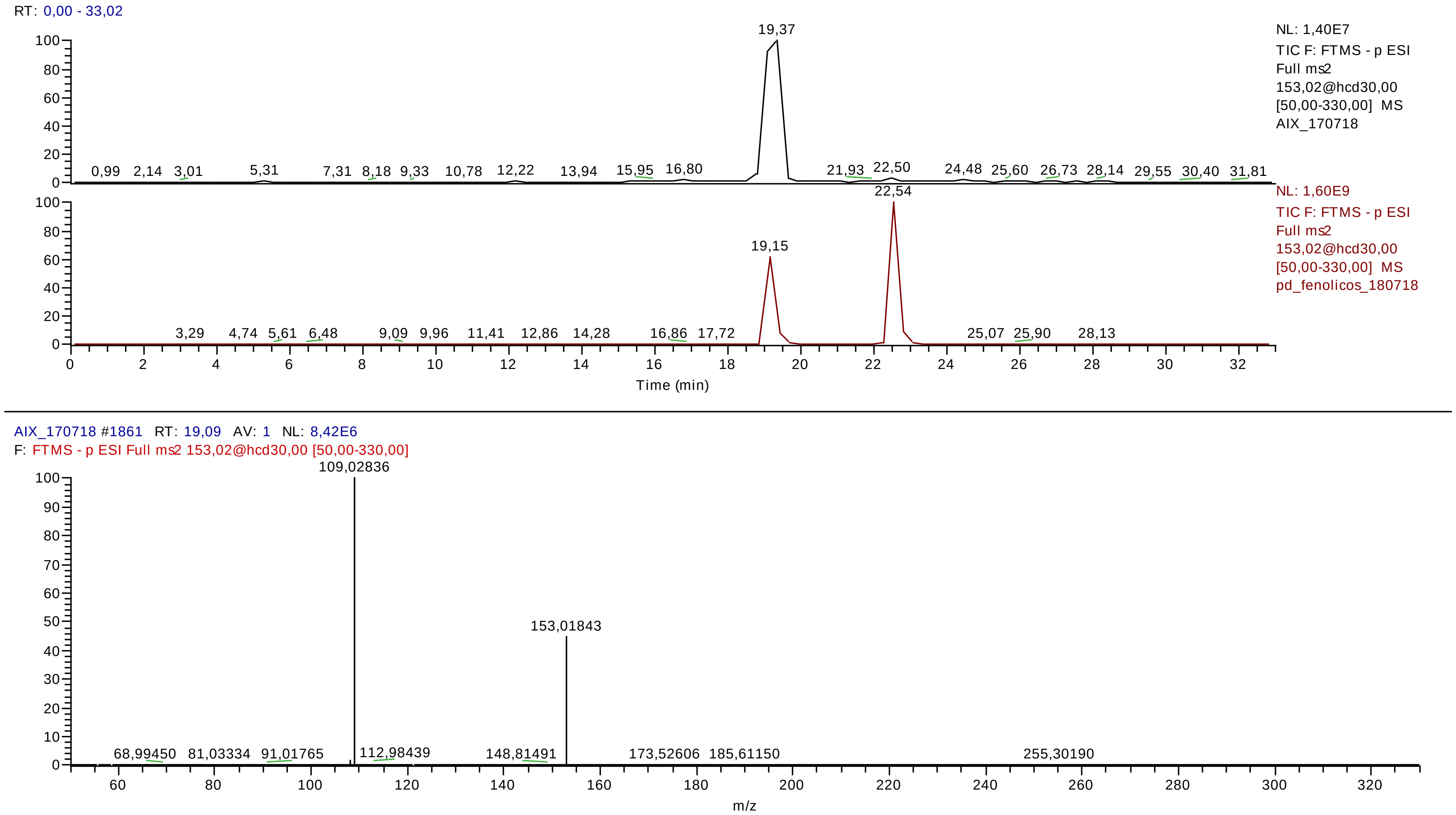

C:\Users\...\Claudiane\AI

17/07/2018 12:01:30

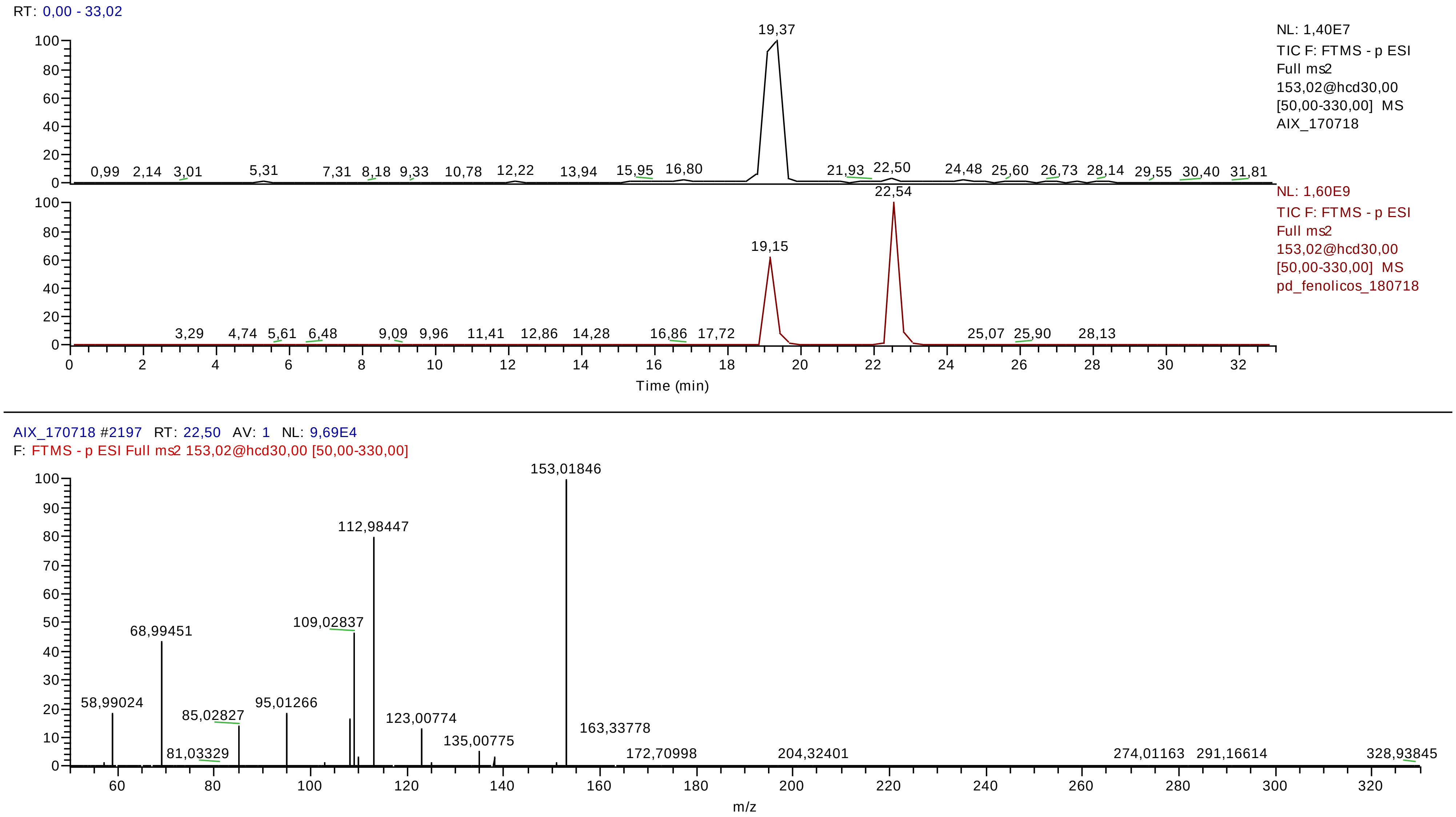

C:\Users\...\Claudiane\AI

17/07/2018 12:01:30

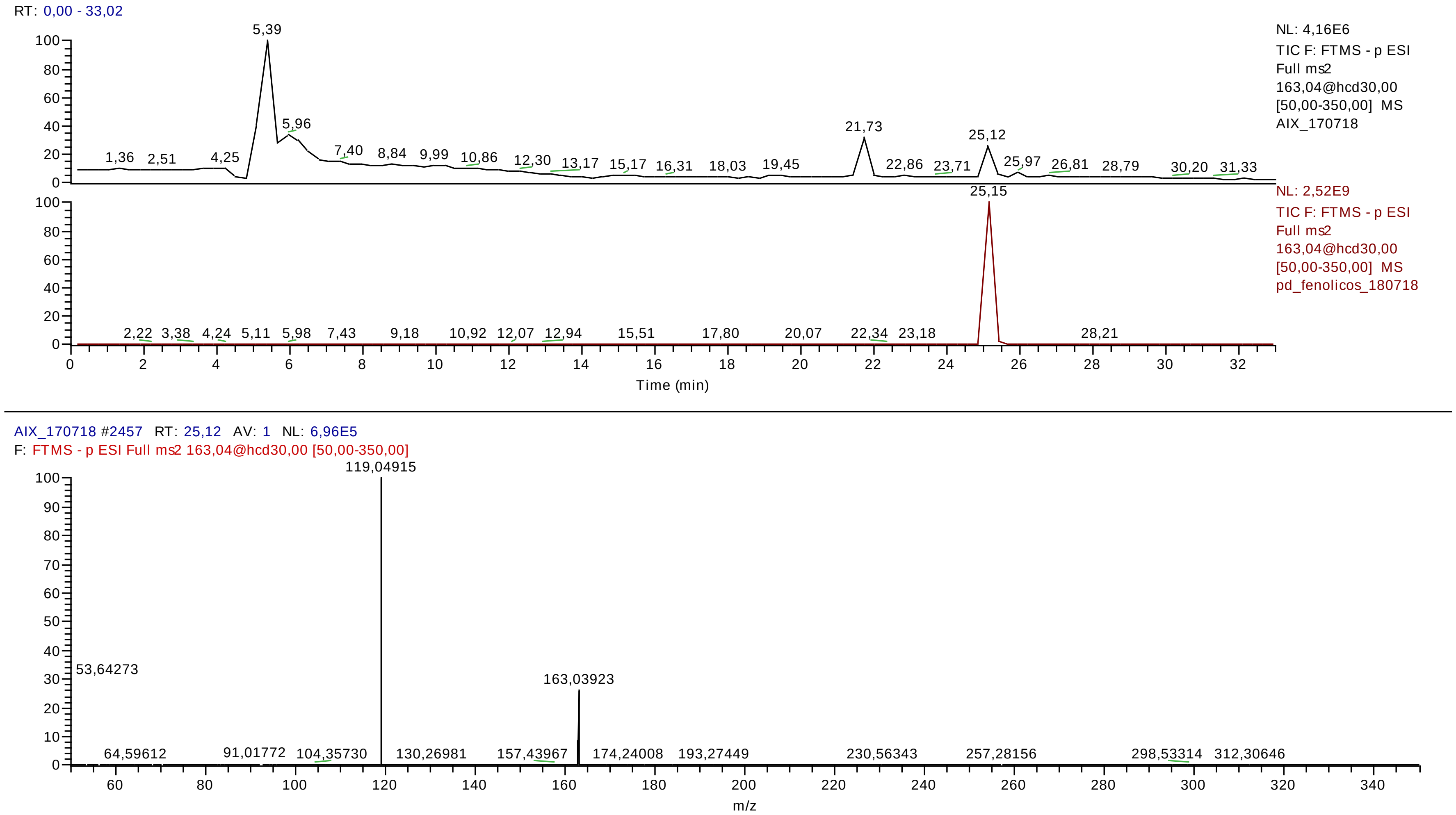

C:\Users\...\Claudiane\AI 17/07/2018 12:01:30

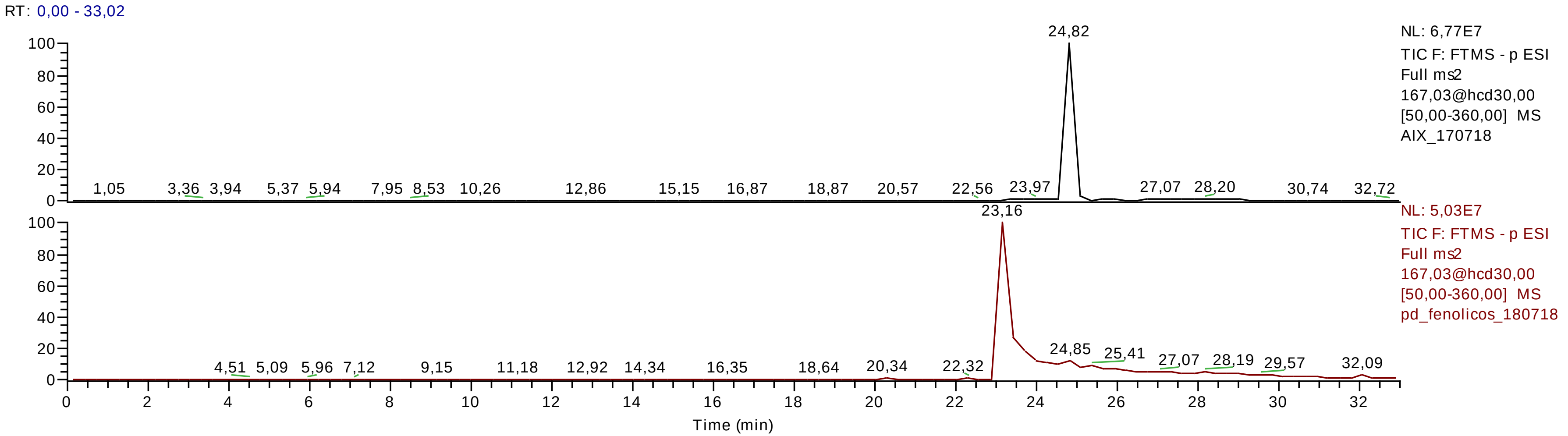

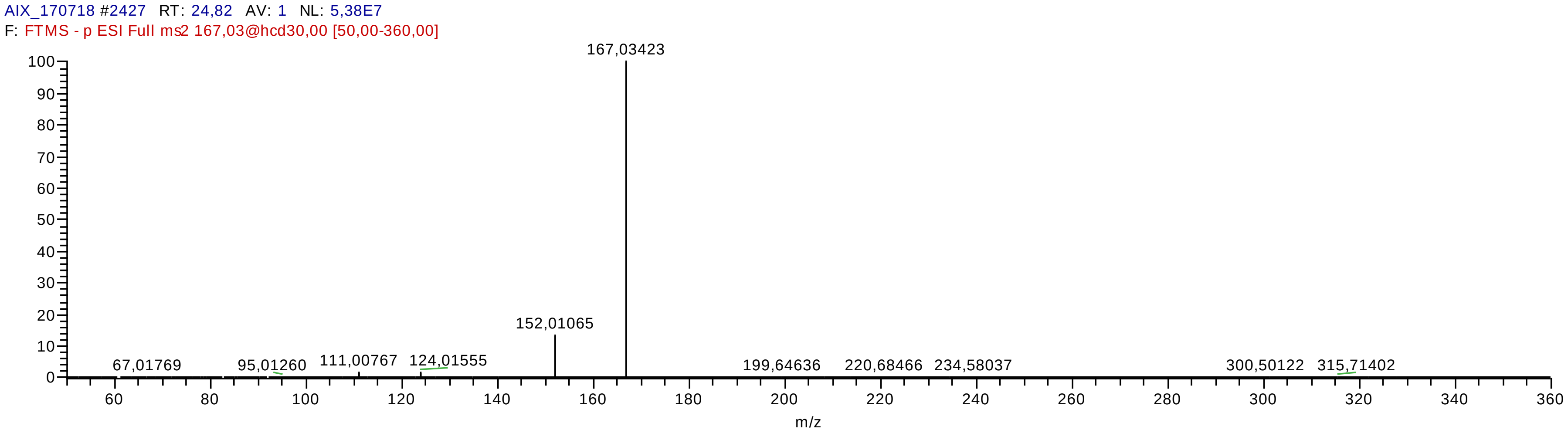

C:\Users\...\Claudiane\AI   17/07/2018 12:01:30

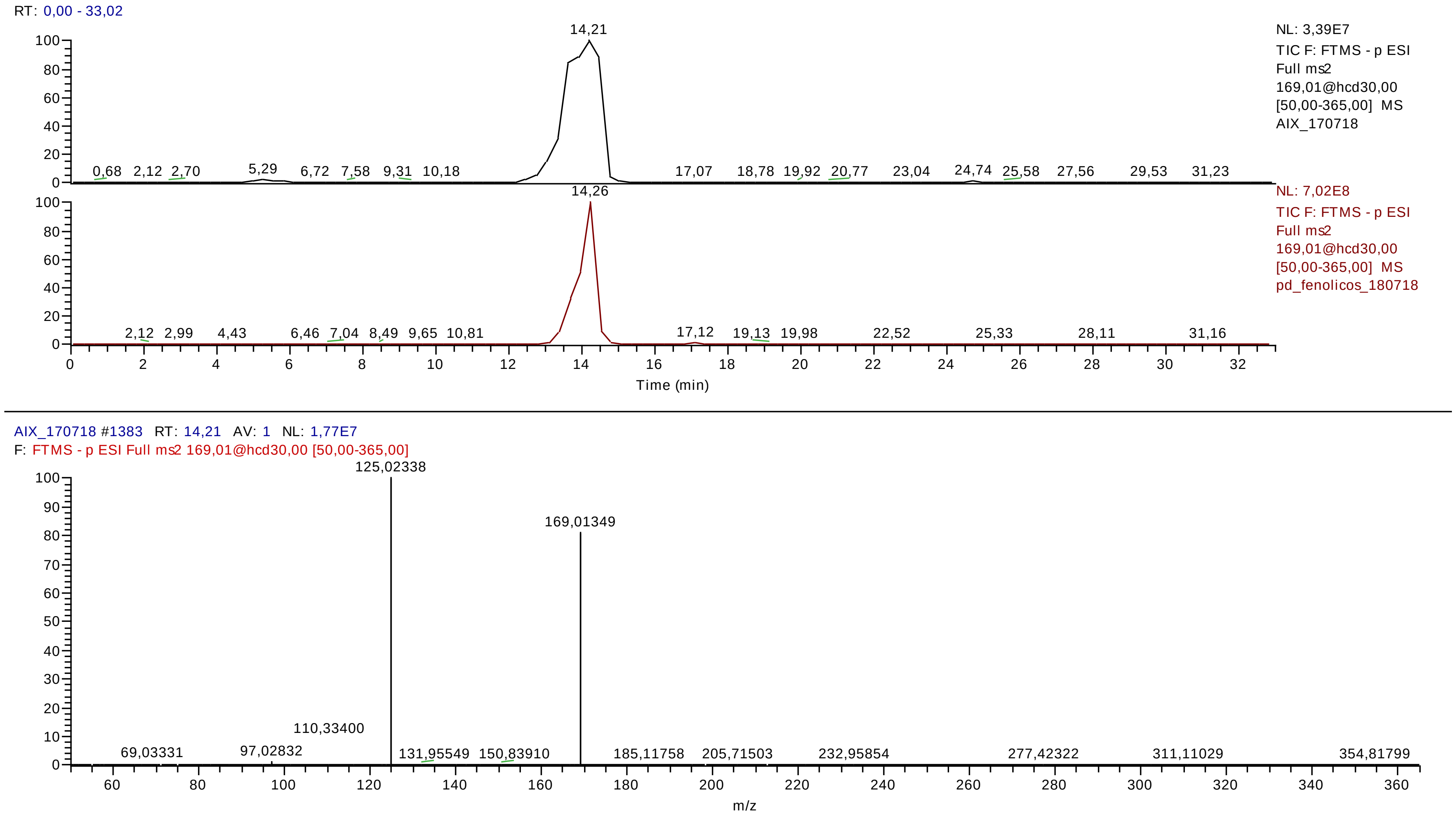

C:\Users\...\Claudiane\AI 17/07/2018 12:01:30

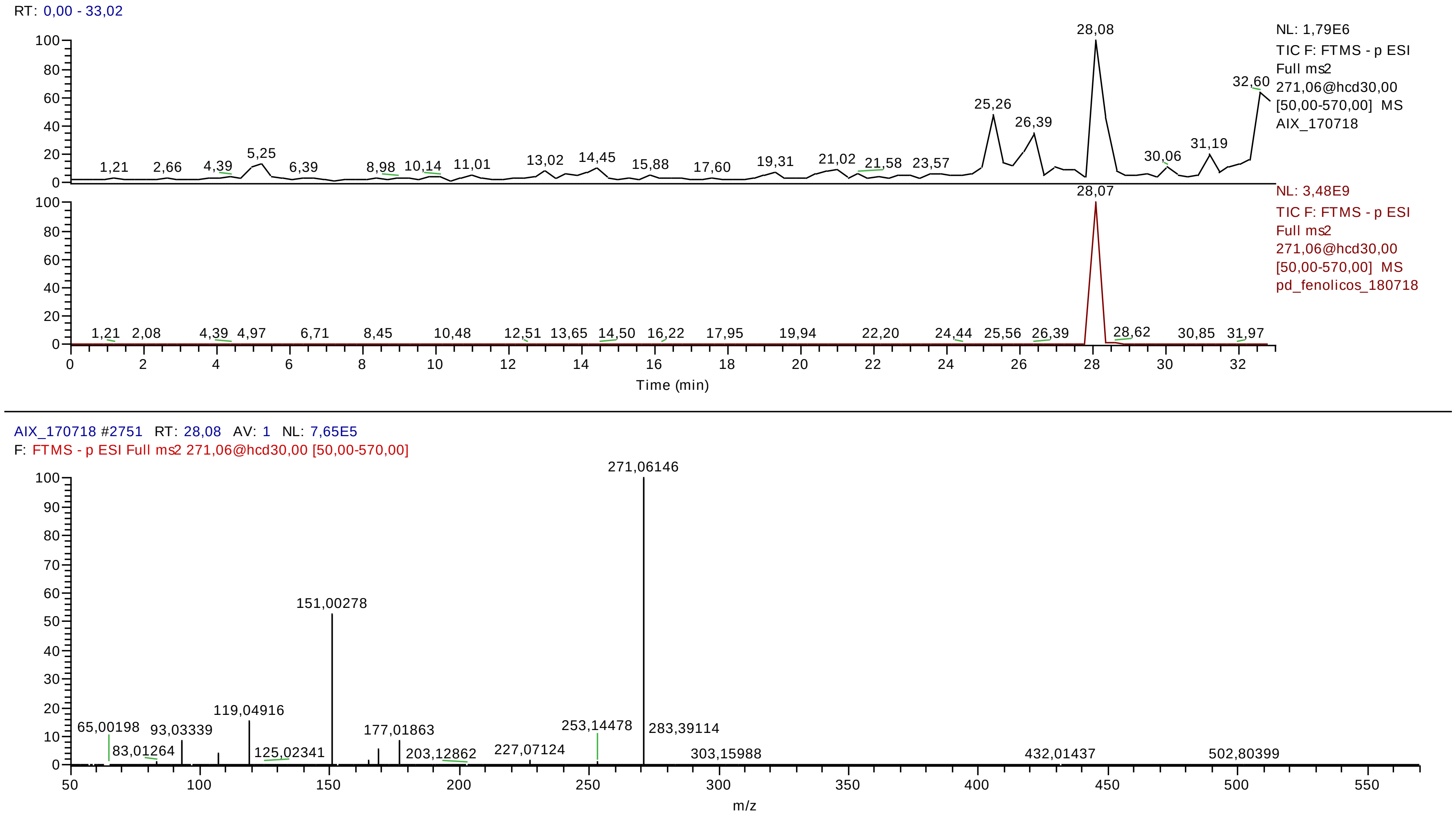

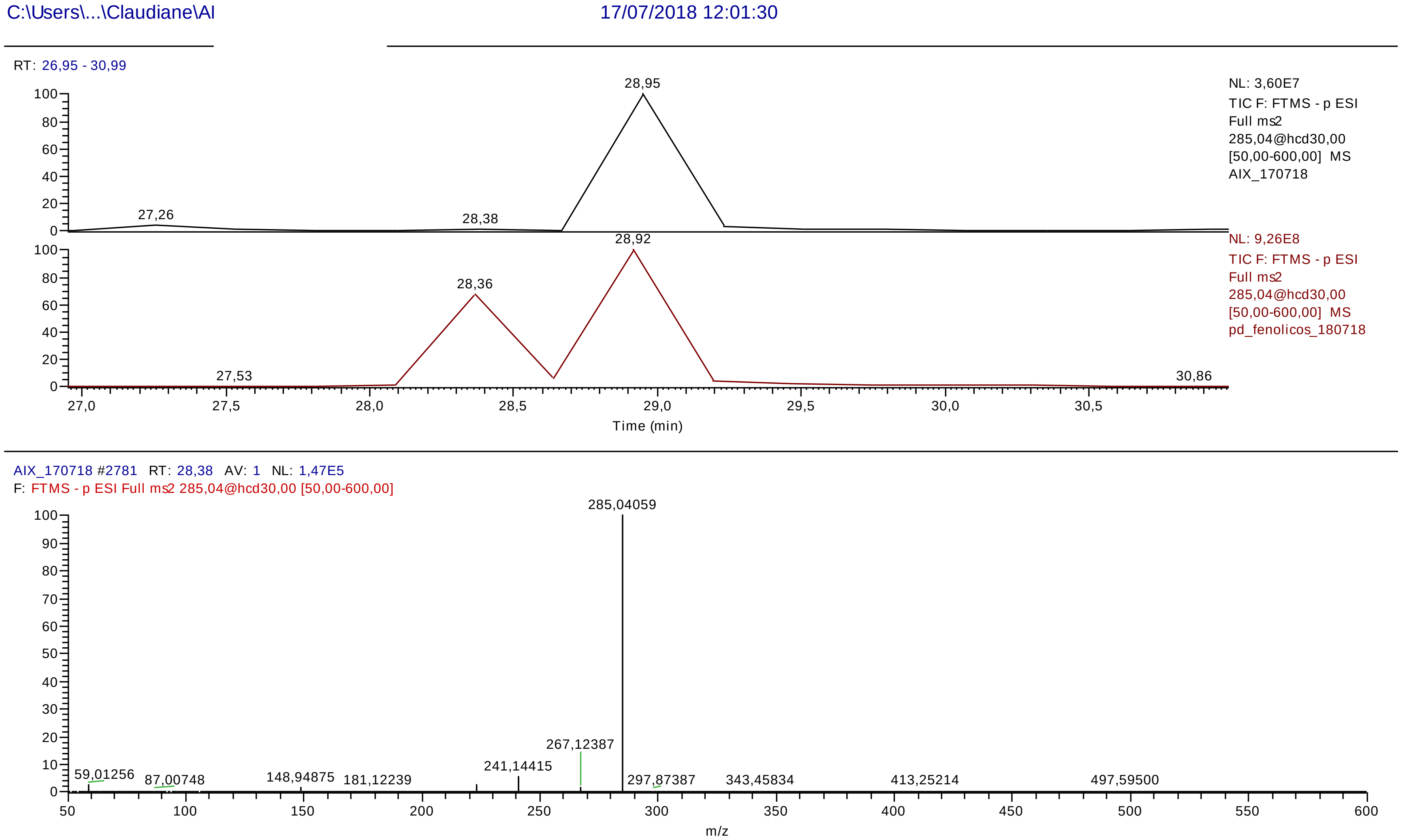
C:\Users\...\Claudiane\AI
17/07/2018 12:01:30
RT: 26,95 - 30,99
NL: 3,60E7
TIC F: FTMS - p ESI
Full ms2
285,04@hcd30,00
[50,00-600,00] MS
AIX_170718
28,95
27,26
28,38
NL: 9,26E8
TIC F: FTMS - p ESI
Full ms2
285,04@hcd30,00
[50,00-600,00] MS
pd_fenolicos_180718
28,92
28,36
27,53
30,86
Time (min)
AIX_170718 #2781 RT: 28,38 AV: 1 NL: 1,47E5
F: FTMS - p ESI Full ms2 285,04@hcd30,00 [50,00-600,00]
285,04059
267,12387
241,14415
59,01256
87,00748
148,94875
181,12239
297,87387
343,45834
413,25214
497,59500
m/z

C:\Users\...\Claudiane\Al   17/07/2018 12:01:30

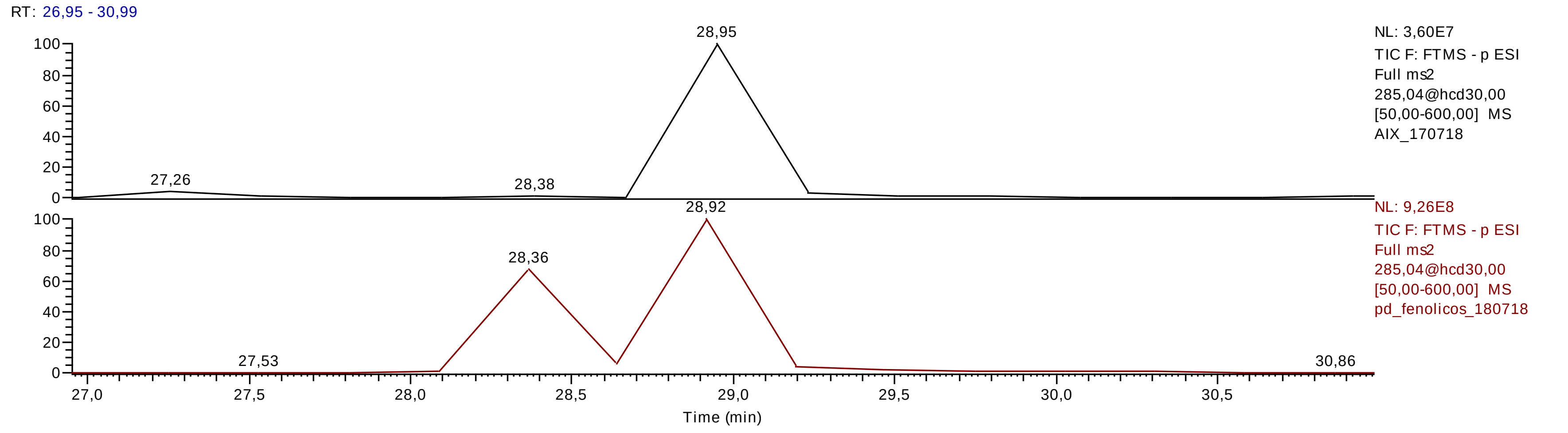

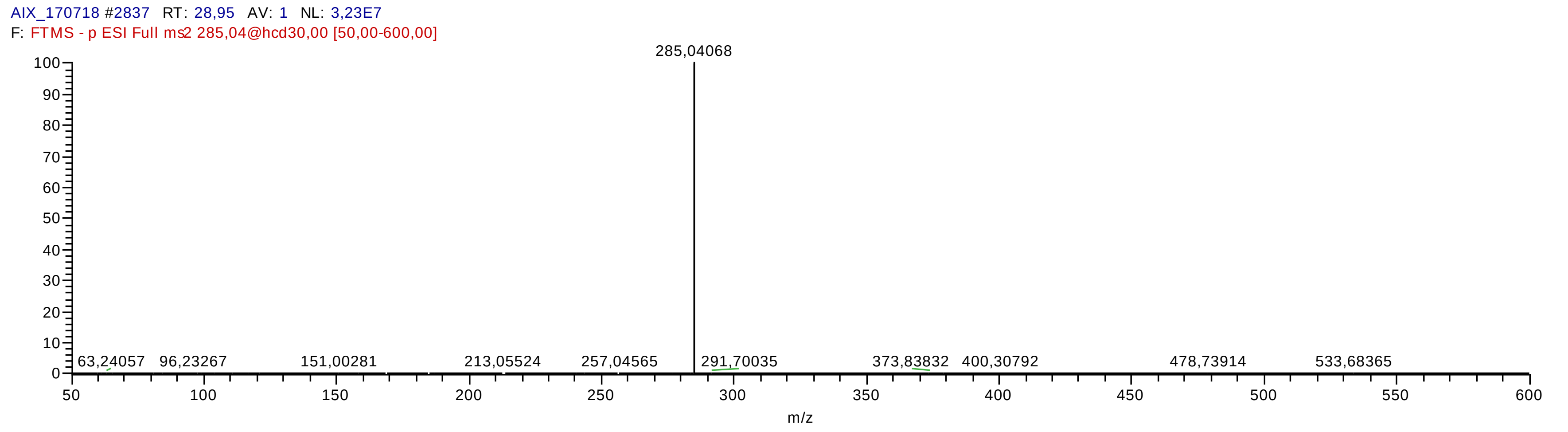

C:\Users\...\Claudiane\AI   17/07/2018 12:01:30

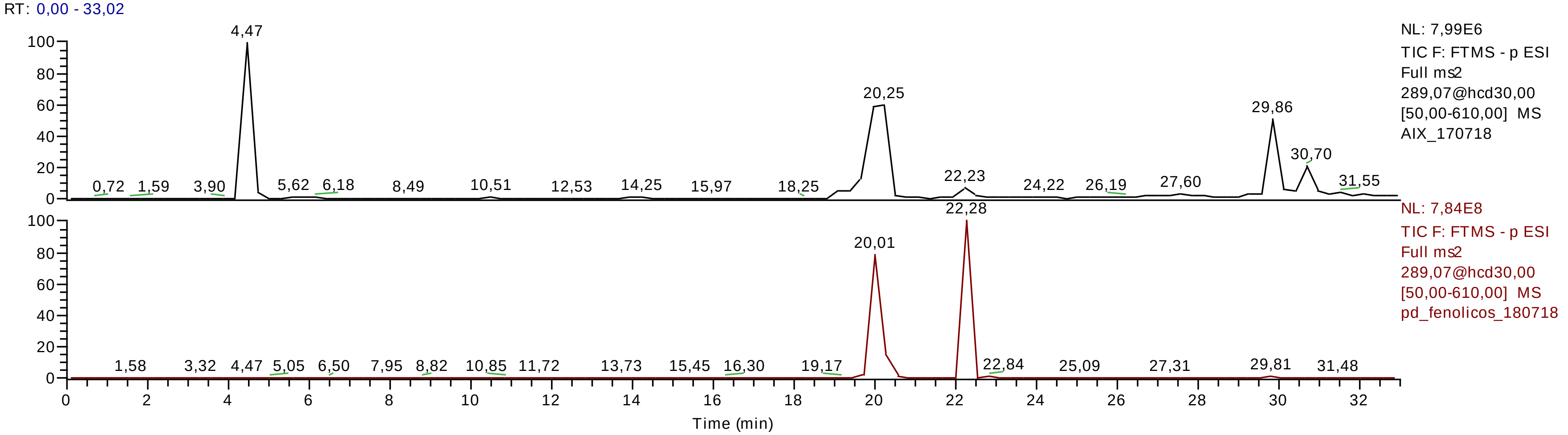

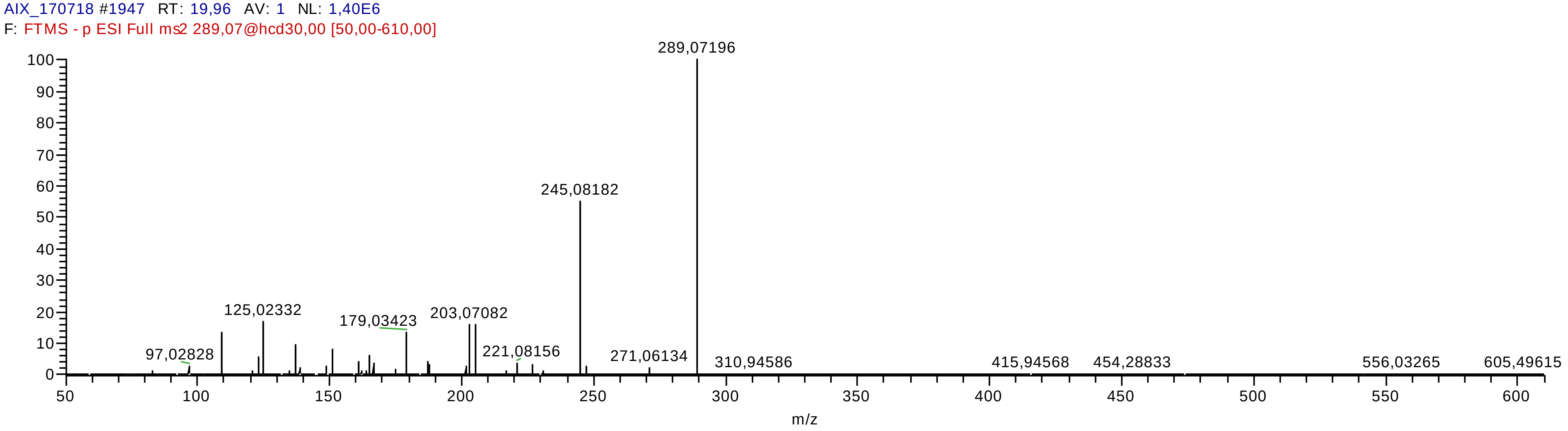

C:\Users\...\Claudiane\AI   17/07/2018 12:01:30

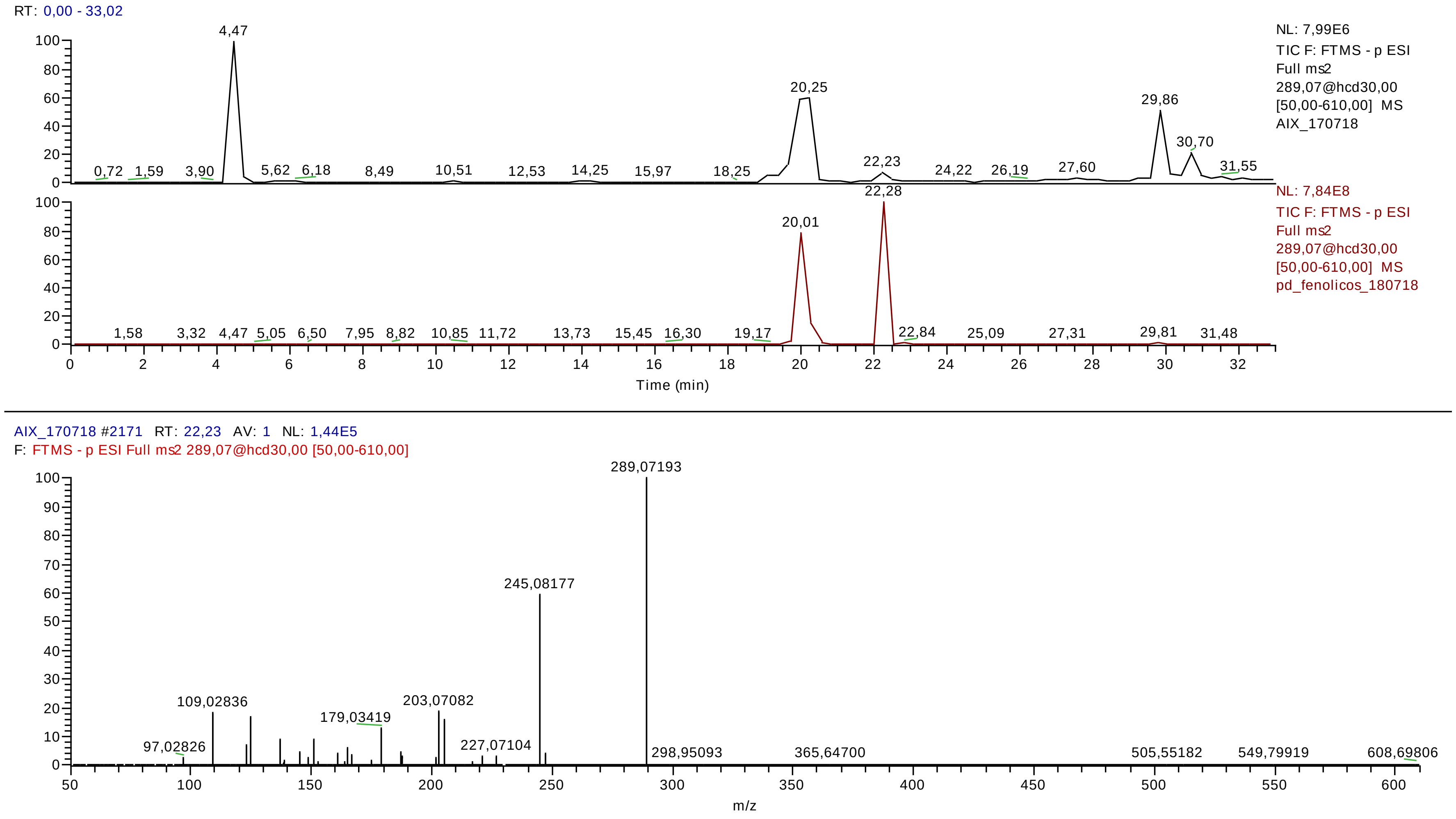

C:\Users\...\Claudiane\AI 17/07/2018 12:01:30

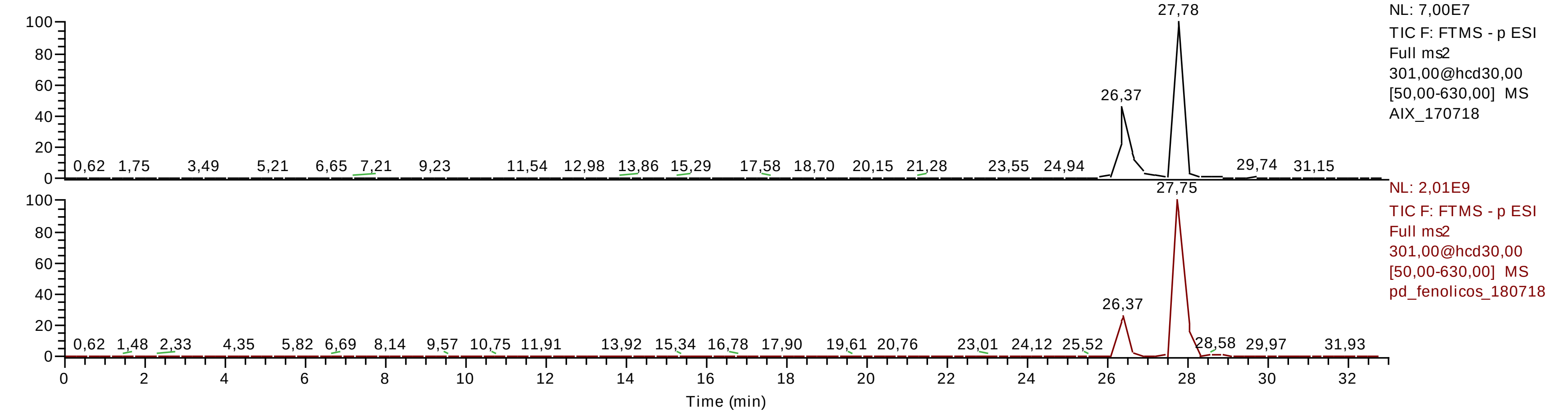

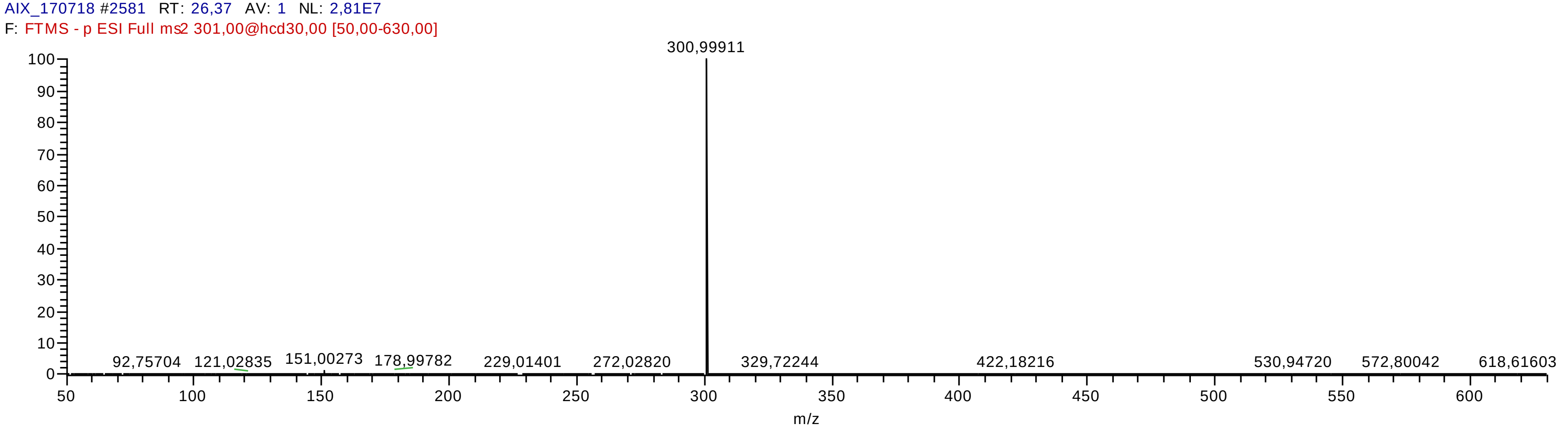

C:\Users\...\Claudiane\AI

17/07/2018 12:01:30

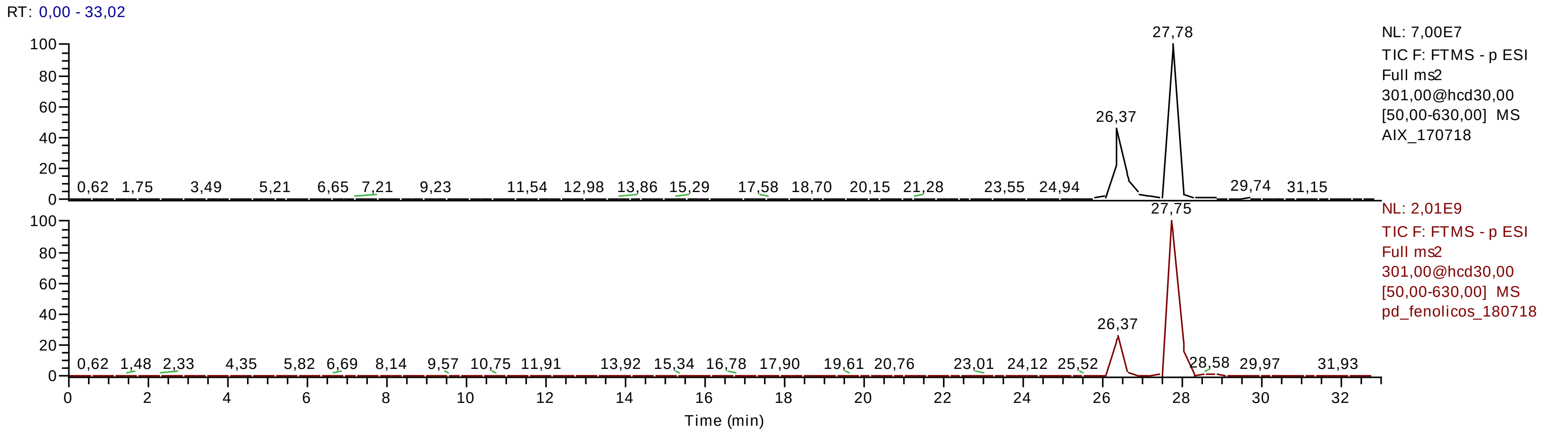

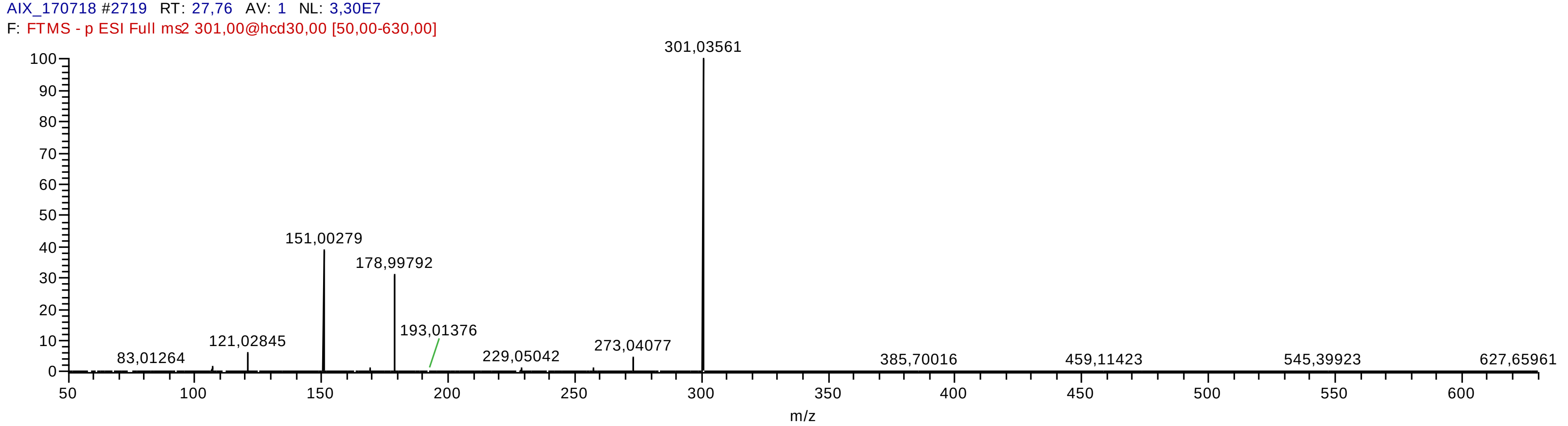

C:\Users\...\Claudiane\AI 17/07/2018 12:01:30

RT: 0,00 - 33,02

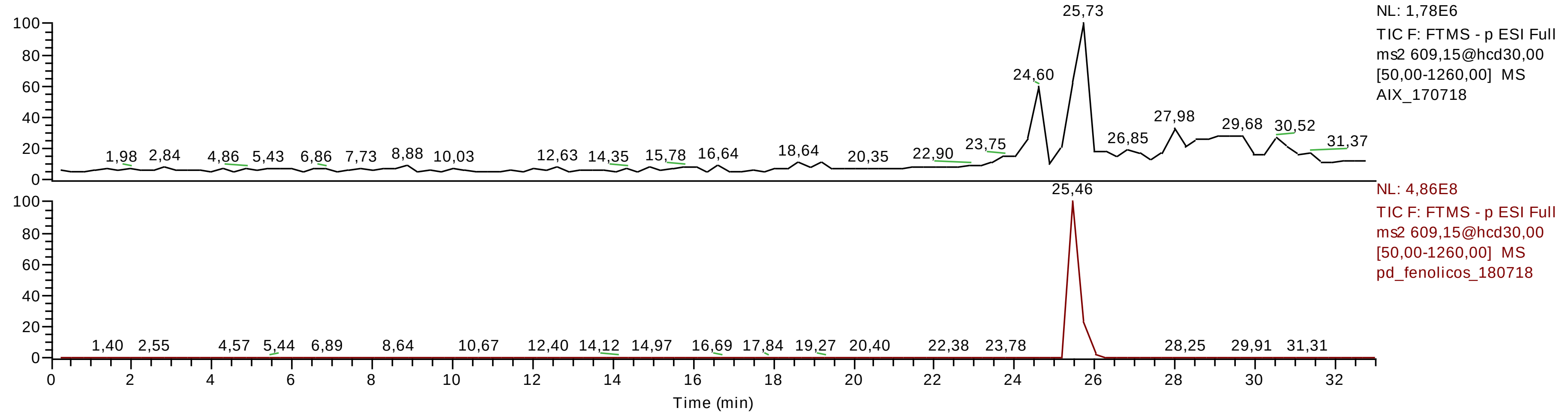

AIX_170718 #2517 RT: 25,73 AV: 1 NL: 5,08E5
F: FTMS - p ESI Full ms2 609,15@hcd30,00 [50,00-1260,00]

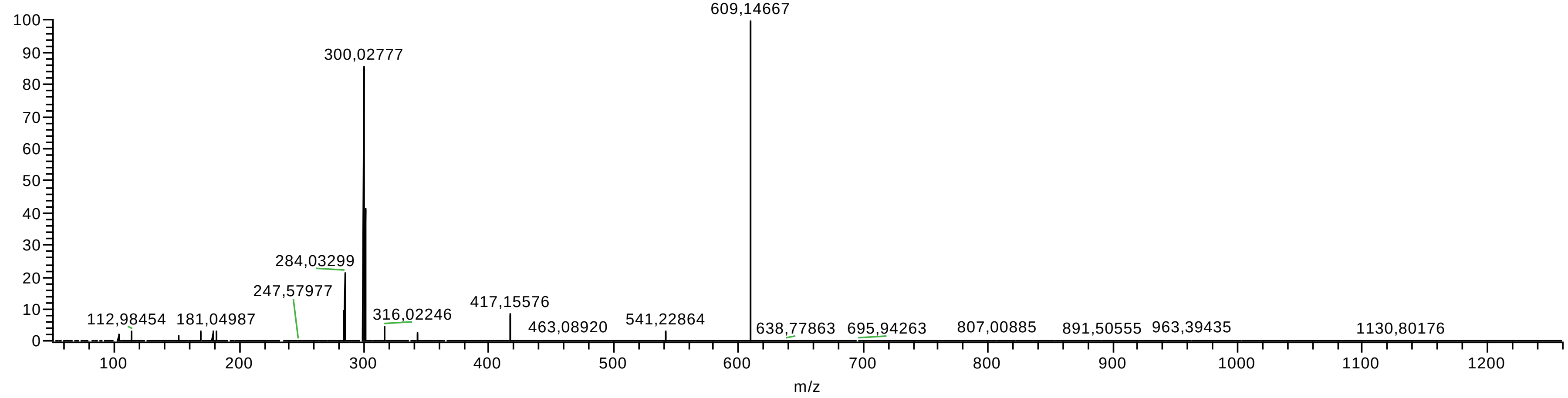

C:\Users\...\Claudiane\A[illegible]

17/07/2018 16:28:26

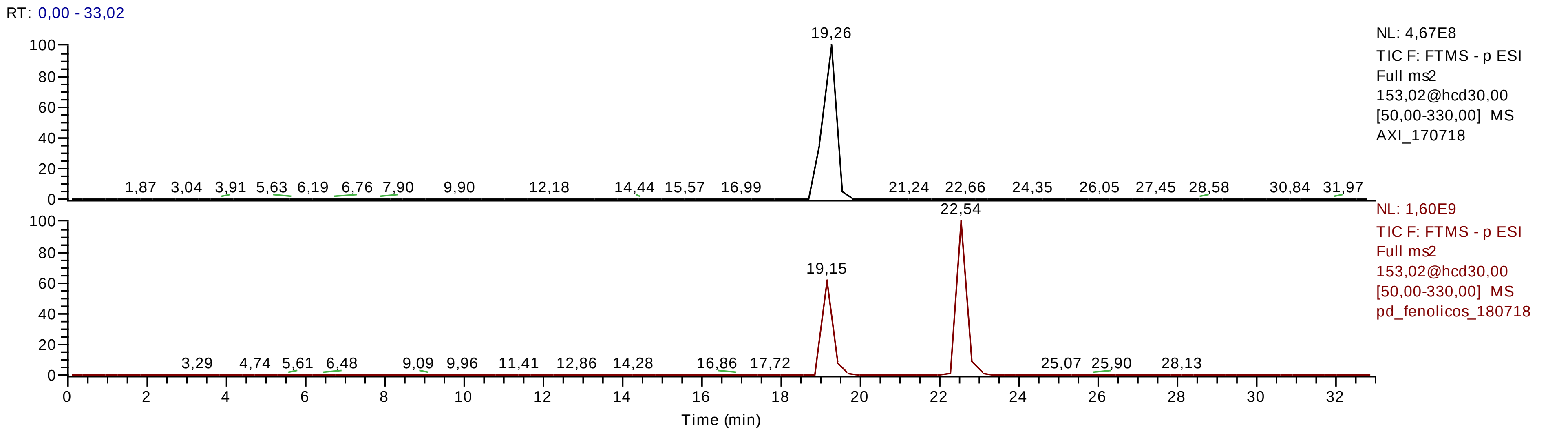

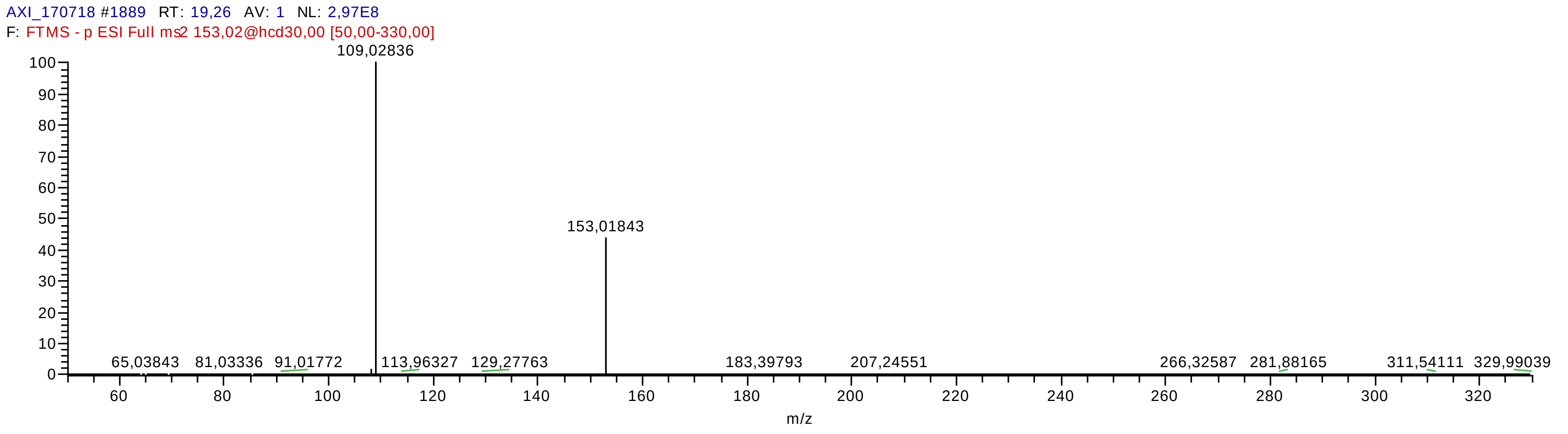

C:\Users\...\Claudiane\A) 17/07/2018 16:28:26

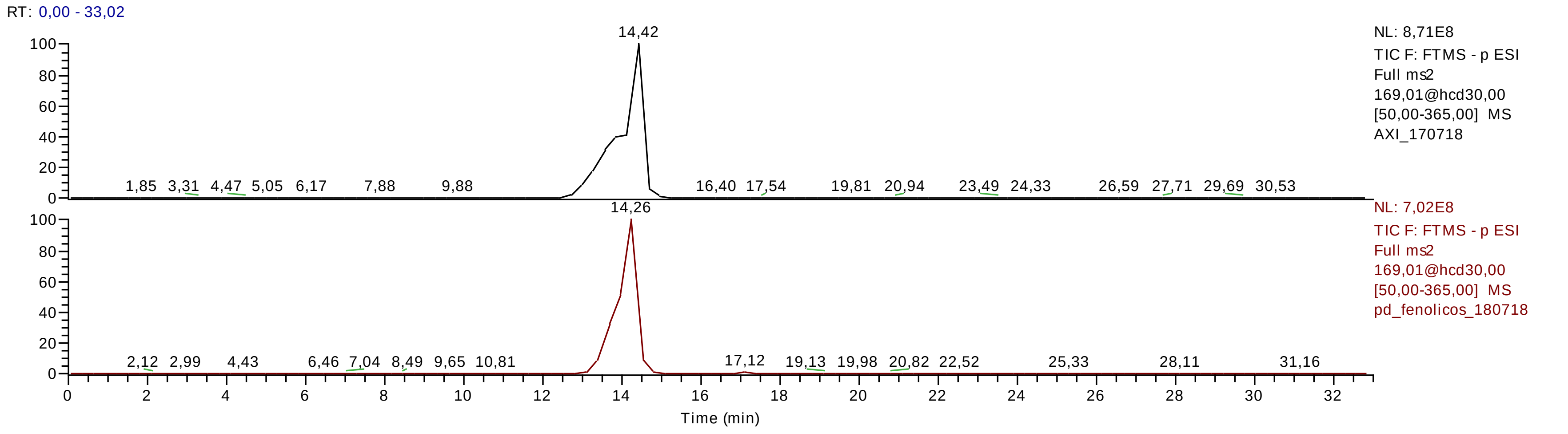

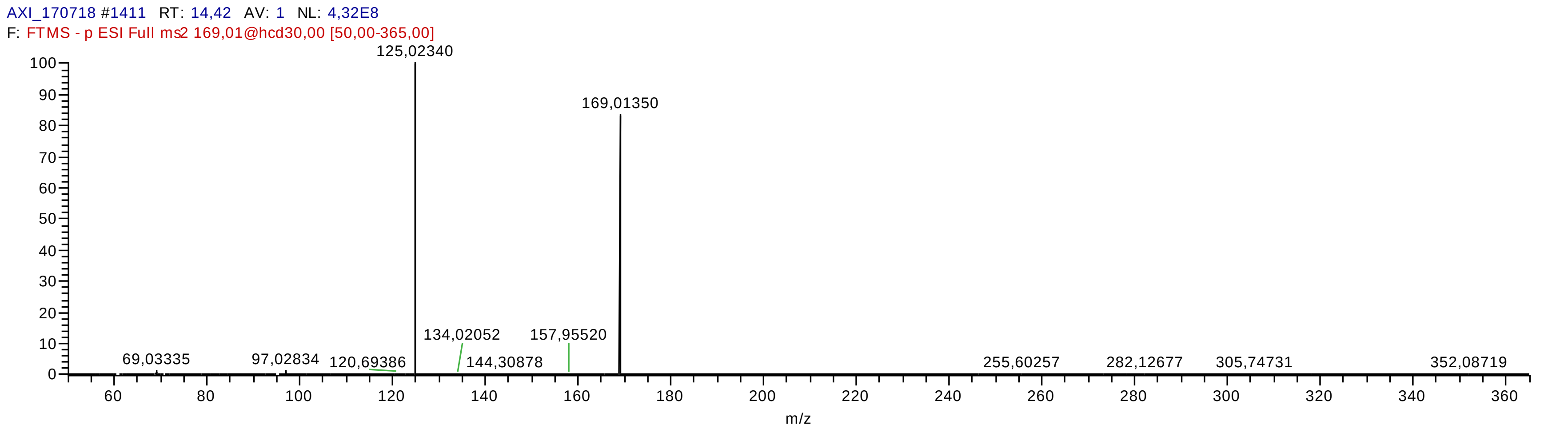

C:\Users\...\Claudiane\AX

17/07/2018 16:28:26

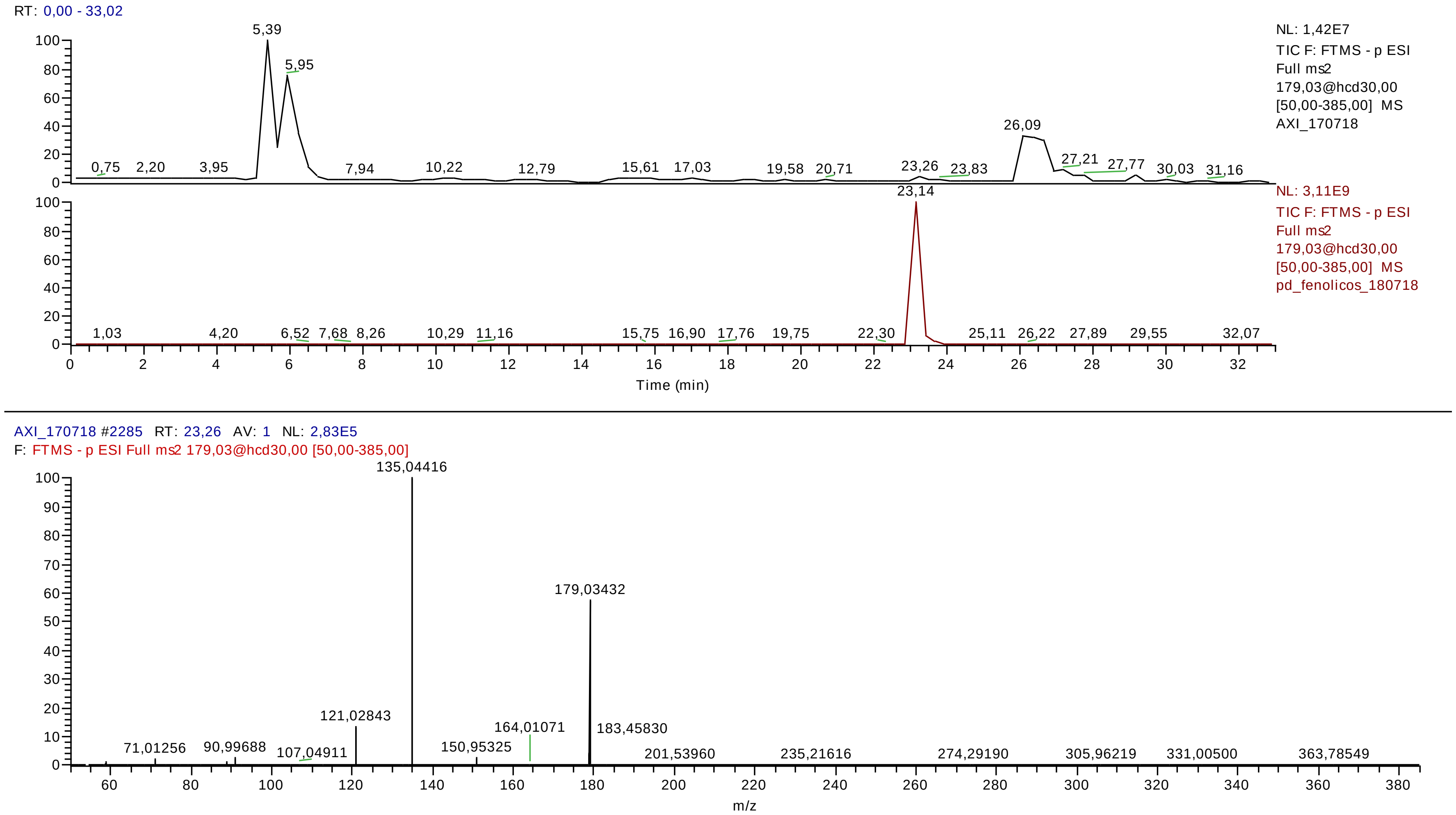

C:\Users\...\Claudiane\A)

17/07/2018 16:28:26

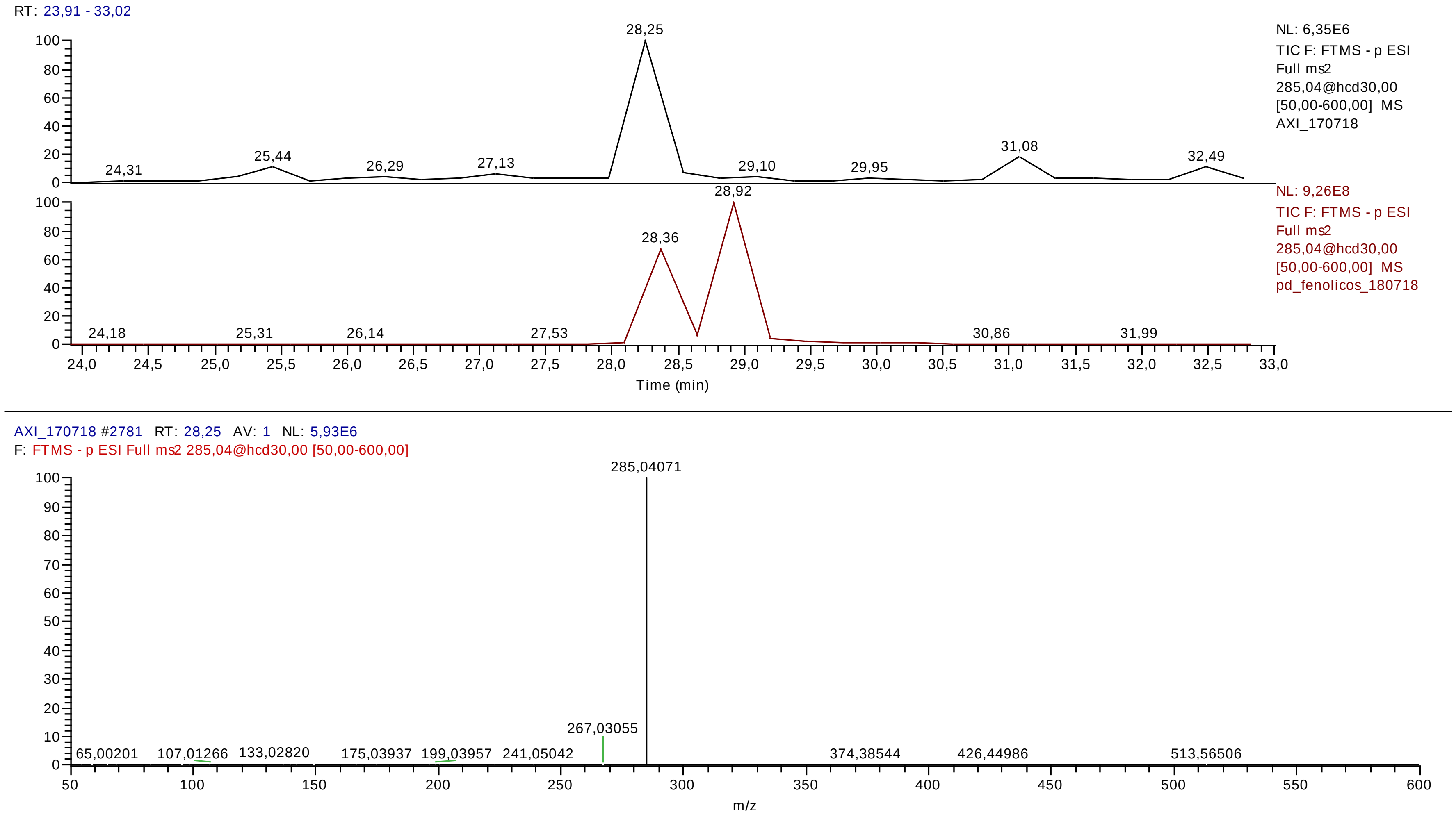

C:\Users\...\Claudiane\A[illegible] 17/07/2018 16:28:26

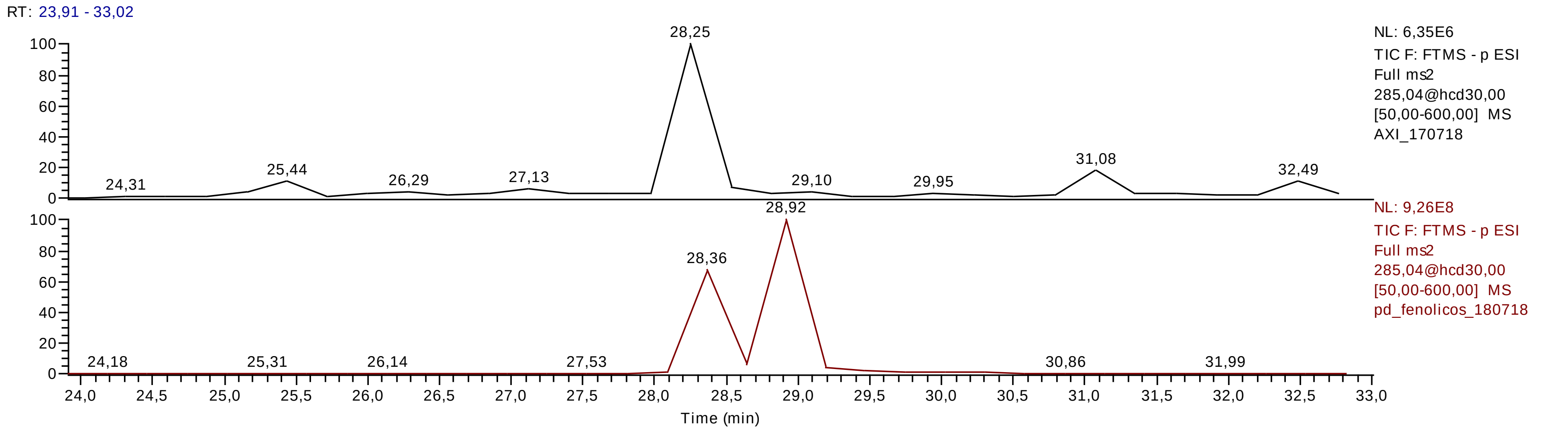

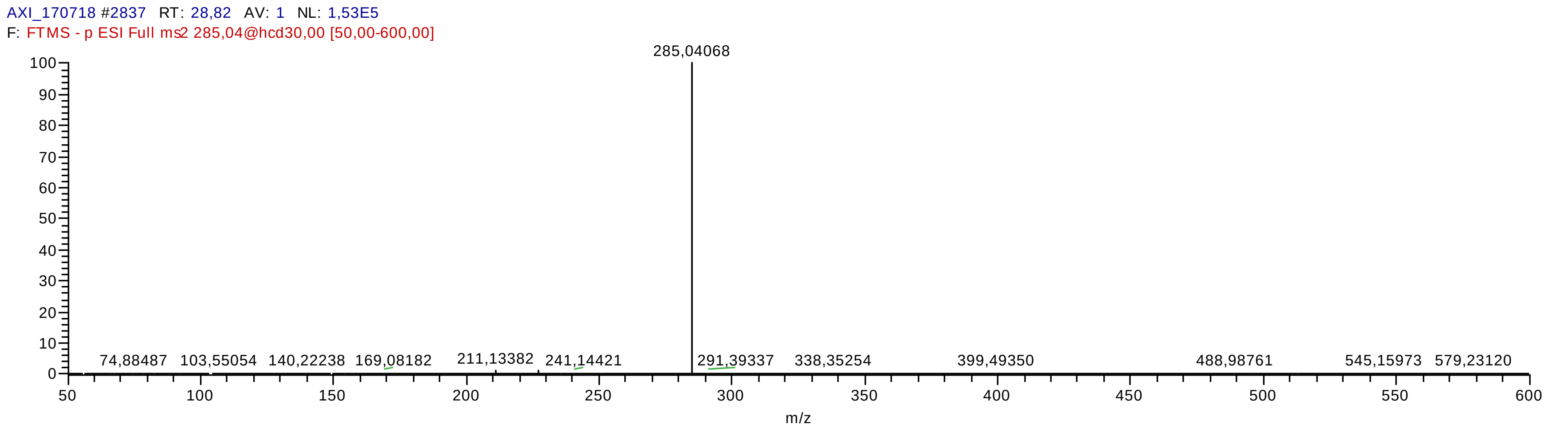

C:\Users\...\Claudiane\A\ 17/07/2018 16:28:26

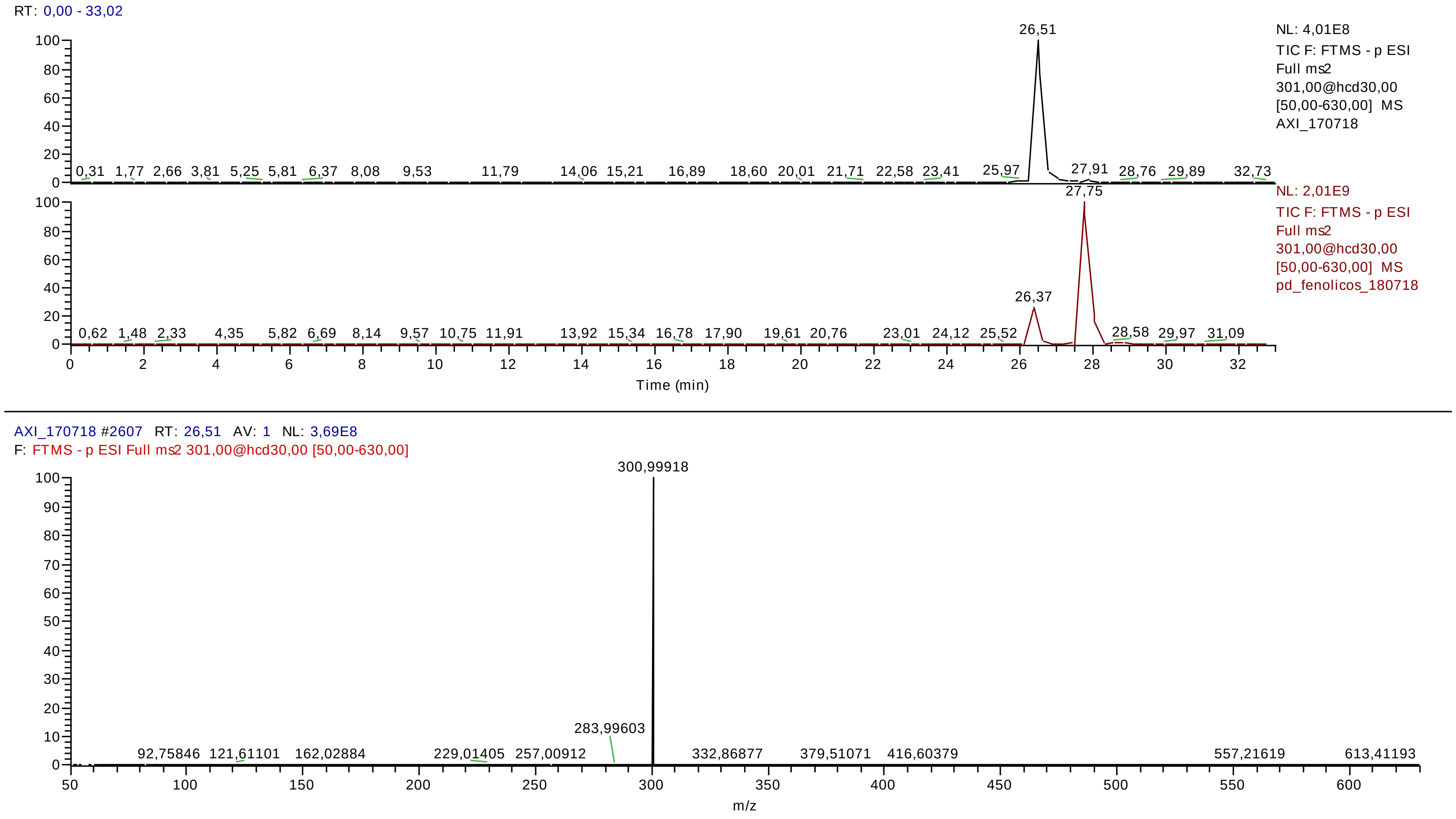

C:\Users\...\Claudiane\A[illegible]

17/07/2018 16:28:26

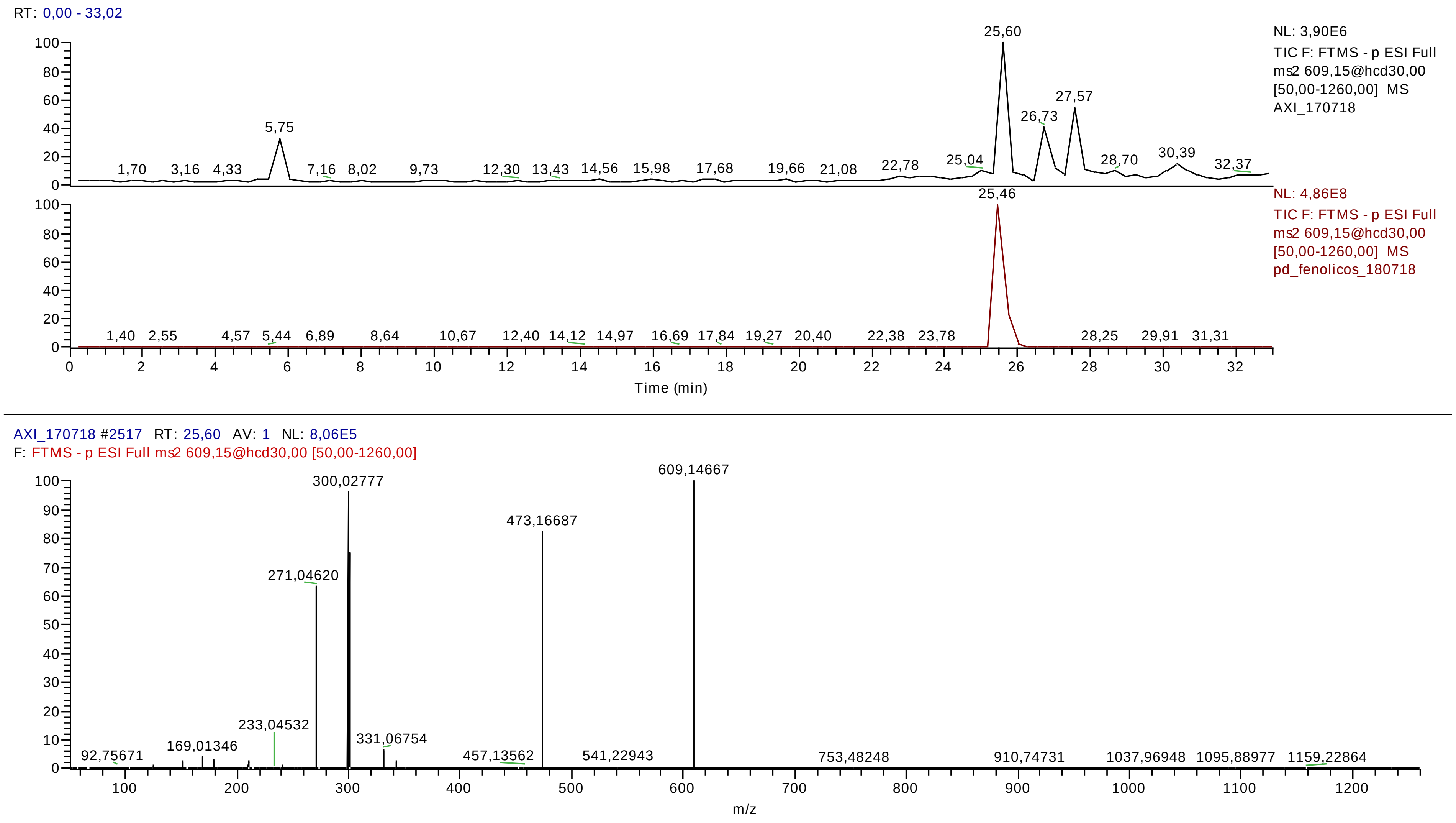

C:\Users\...\Claudiane\A

17/07/2018 17:38:16

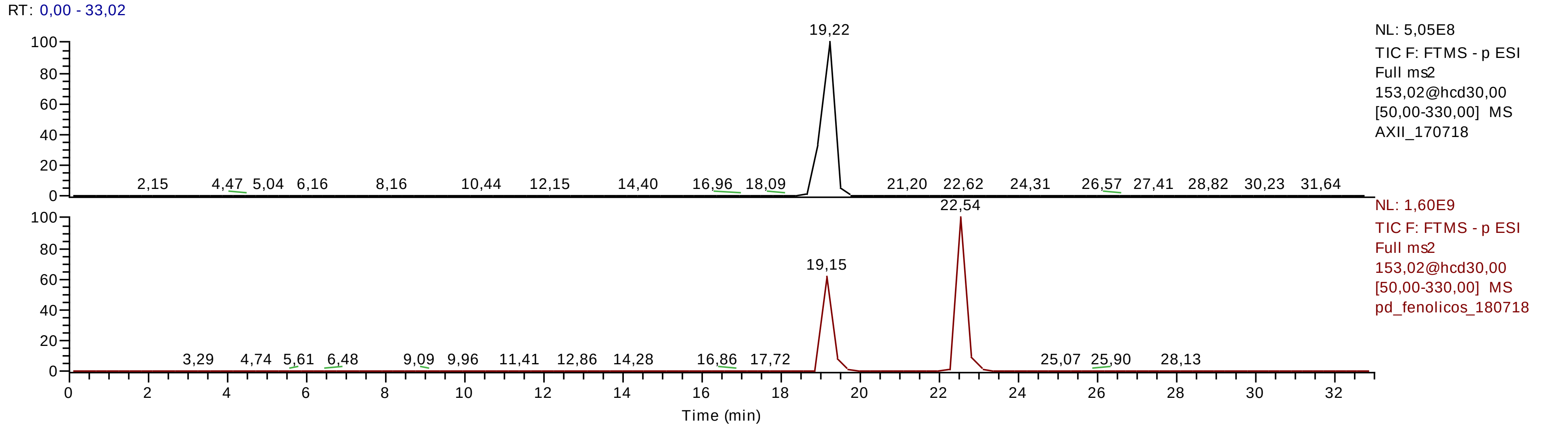

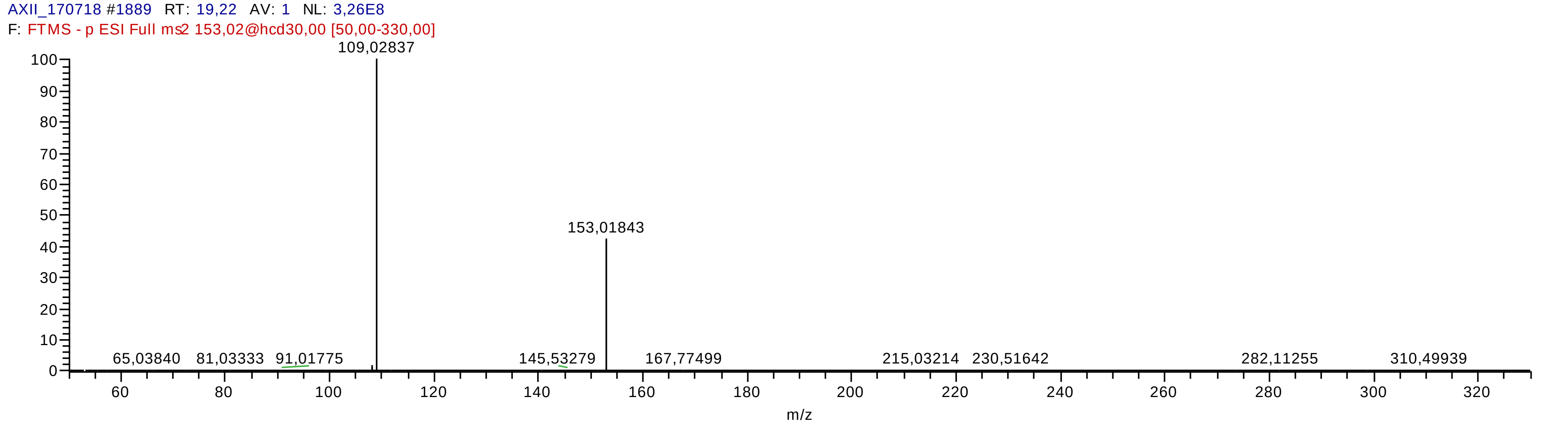

C:\Users\...\Claudiane\A[illegible]   17/07/2018 17:38:16

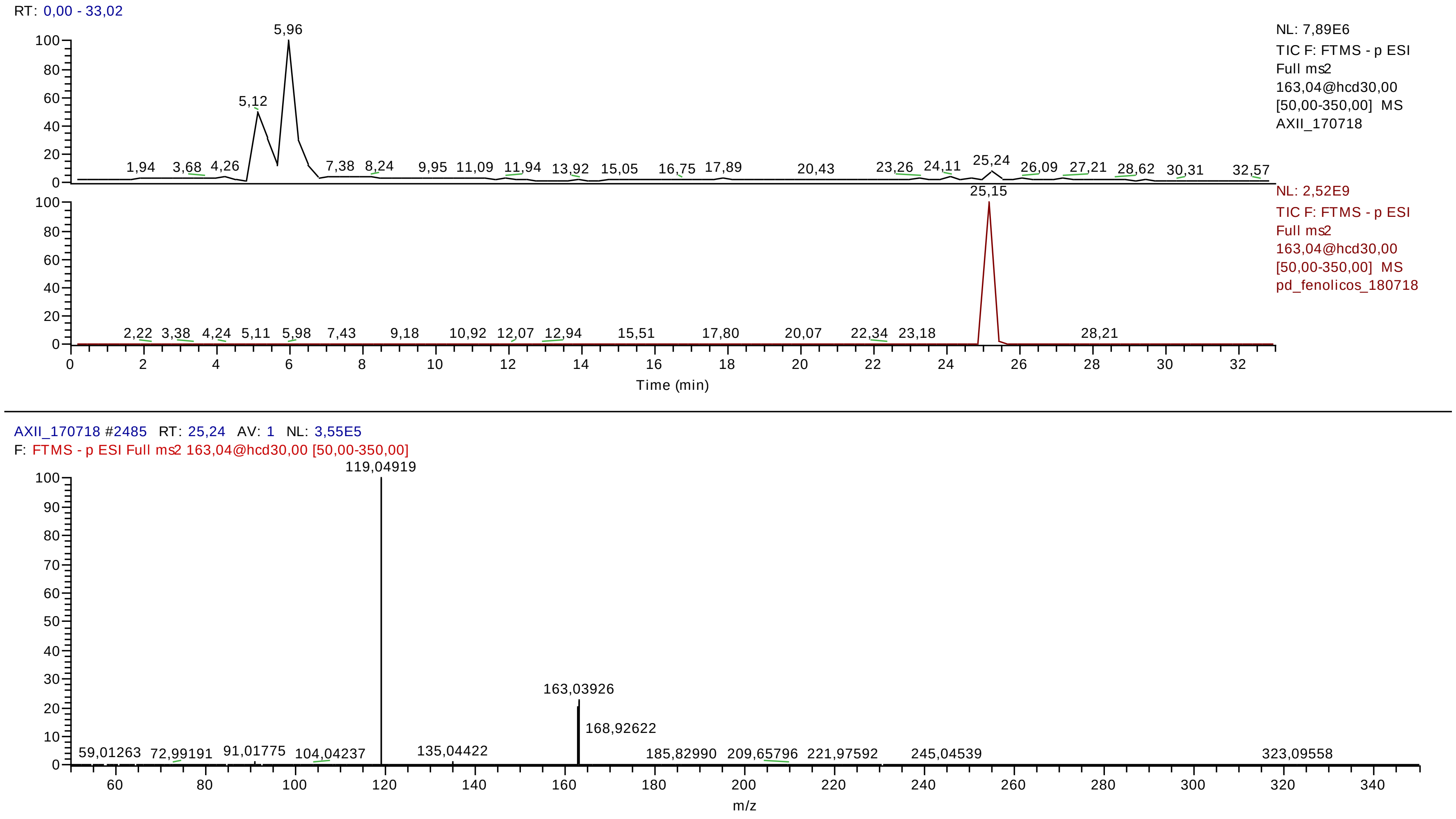

C:\Users\...\Claudiane\A

17/07/2018 17:38:16

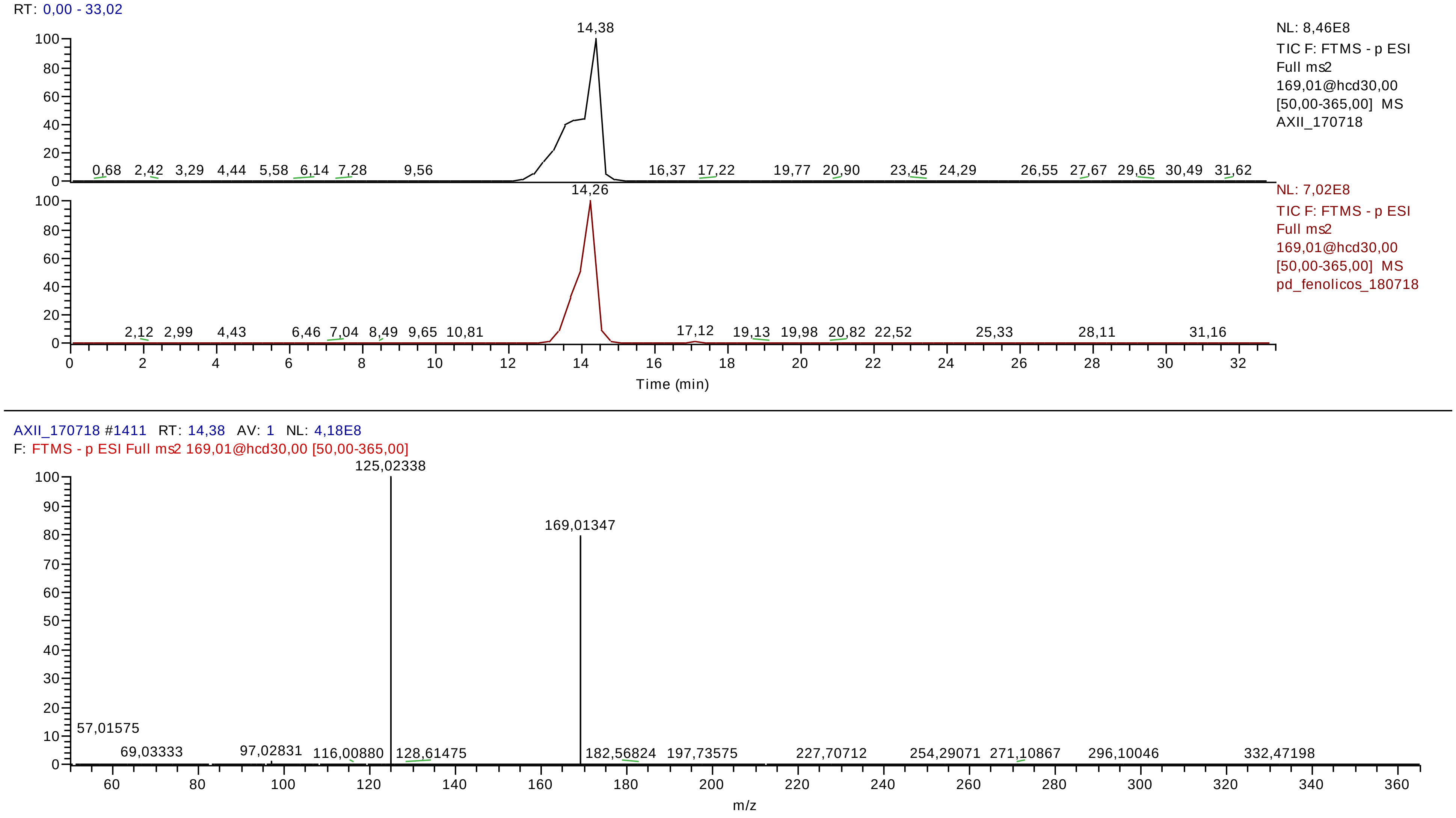

C:\Users\...\Claudiane\A[illegible] 17/07/2018 17:38:16

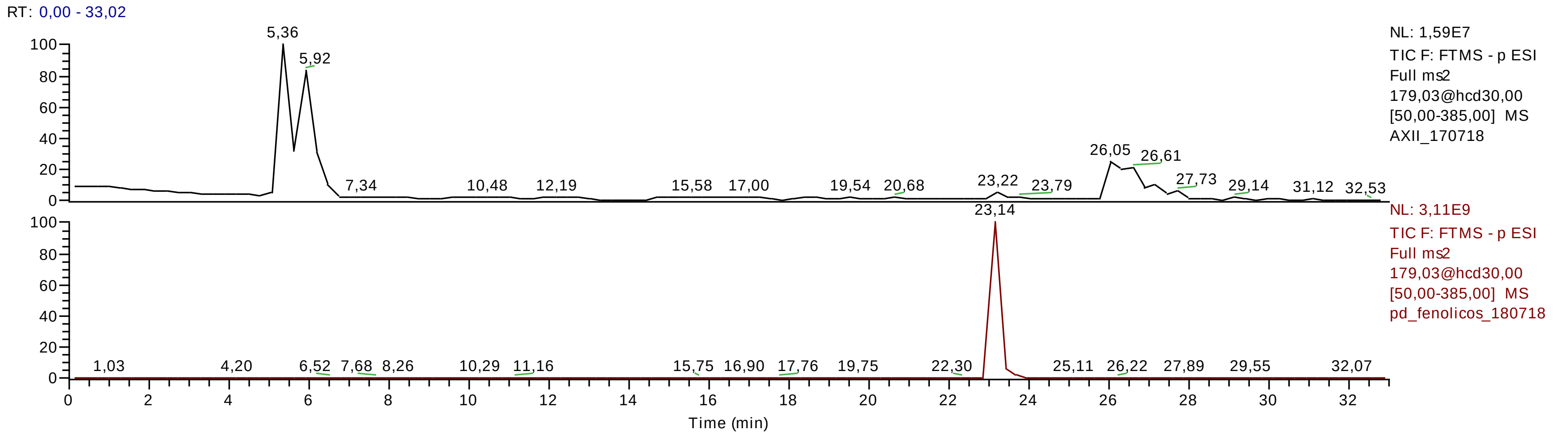

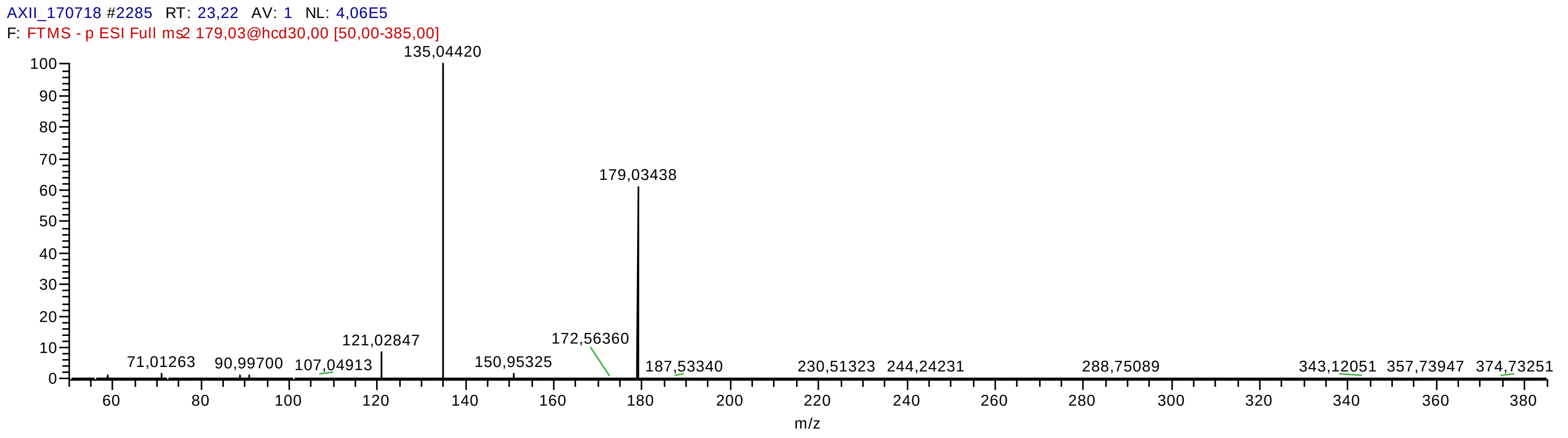

C:\Users\...\Claudiane\A) 17/07/2018 17:38:16

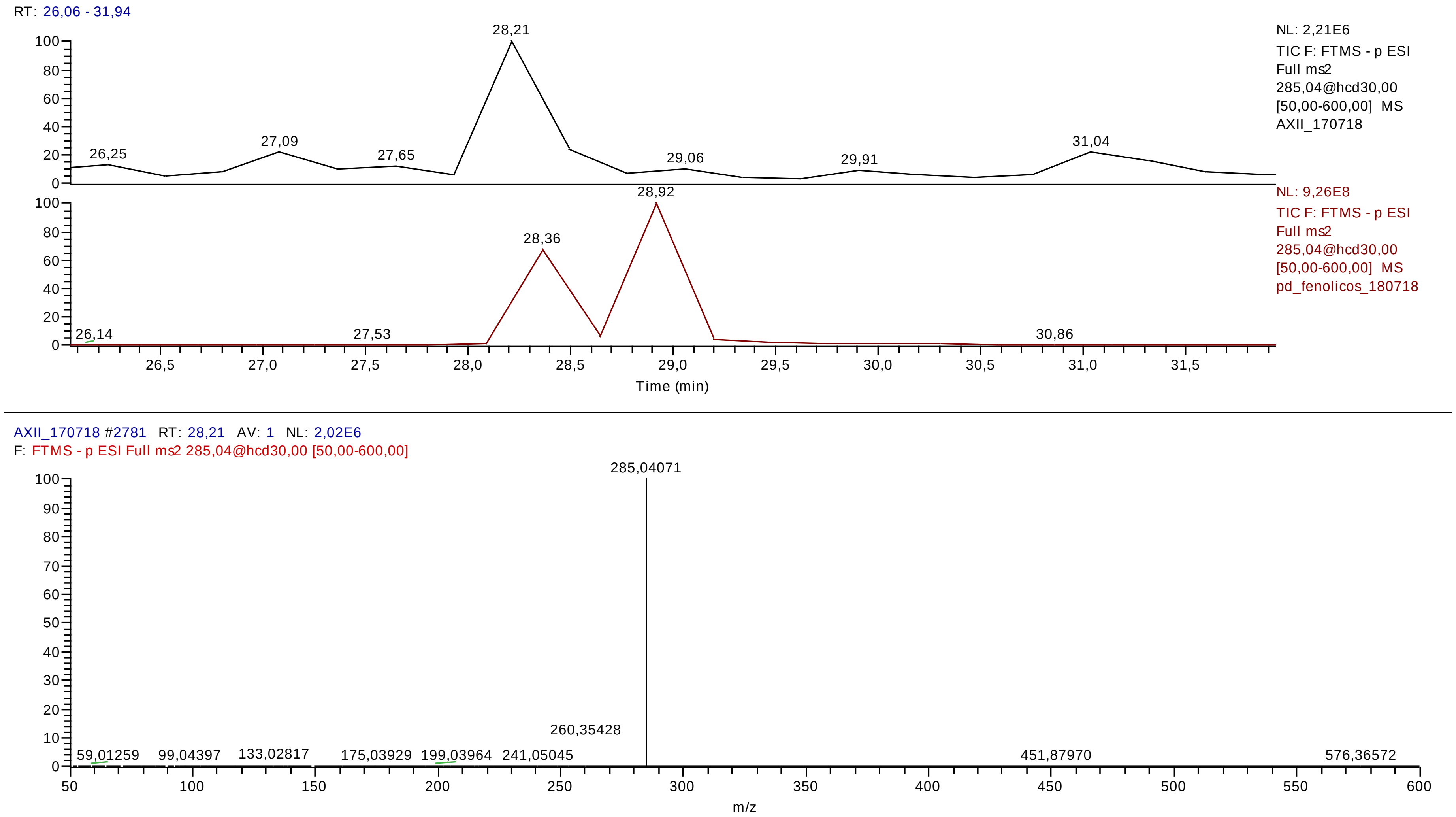

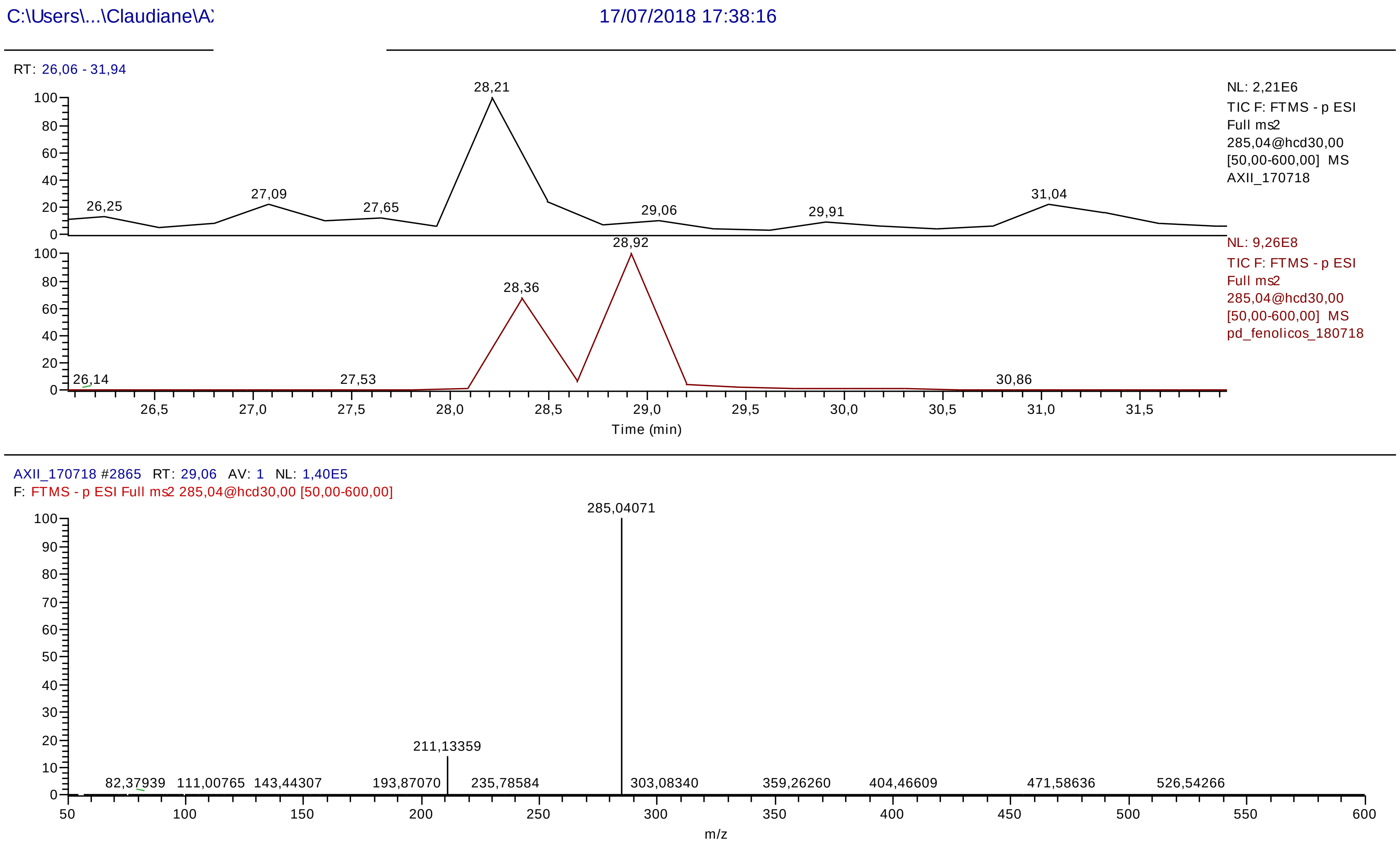
C:\Users\...\Claudiane\AX
17/07/2018 17:38:16
RT: 26,06 - 31,94
NL: 2,21E6
TIC F: FTMS - p ESI Full ms2 285,04@hcd30,00 [50,00-600,00] MS AXII_170718
28,21
26,25
27,09
27,65
29,06
29,91
31,04
NL: 9,26E8
TIC F: FTMS - p ESI Full ms2 285,04@hcd30,00 [50,00-600,00] MS pd_fenolicos_180718
28,92
28,36
26,14
27,53
30,86
Time (min)
AXII_170718 #2865 RT: 29,06 AV: 1 NL: 1,40E5
F: FTMS - p ESI Full ms2 285,04@hcd30,00 [50,00-600,00]
285,04071
211,13359
82,37939
111,00765
143,44307
193,87070
235,78584
303,08340
359,26260
404,46609
471,58636
526,54266
m/z

C:\Users\...\Claudiane\A[illegible] 17/07/2018 17:38:16

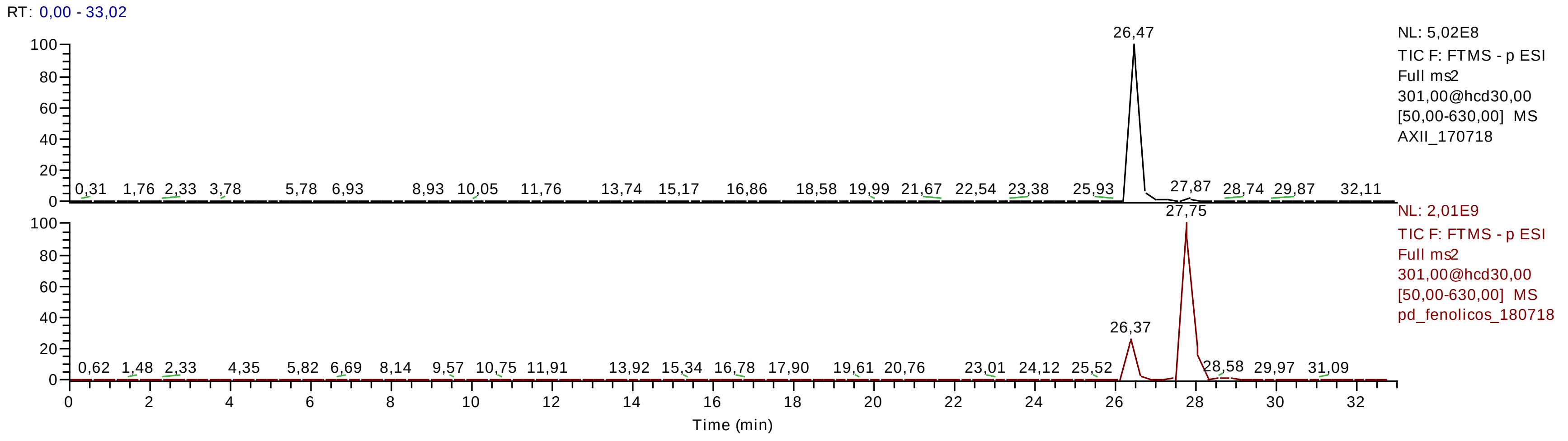

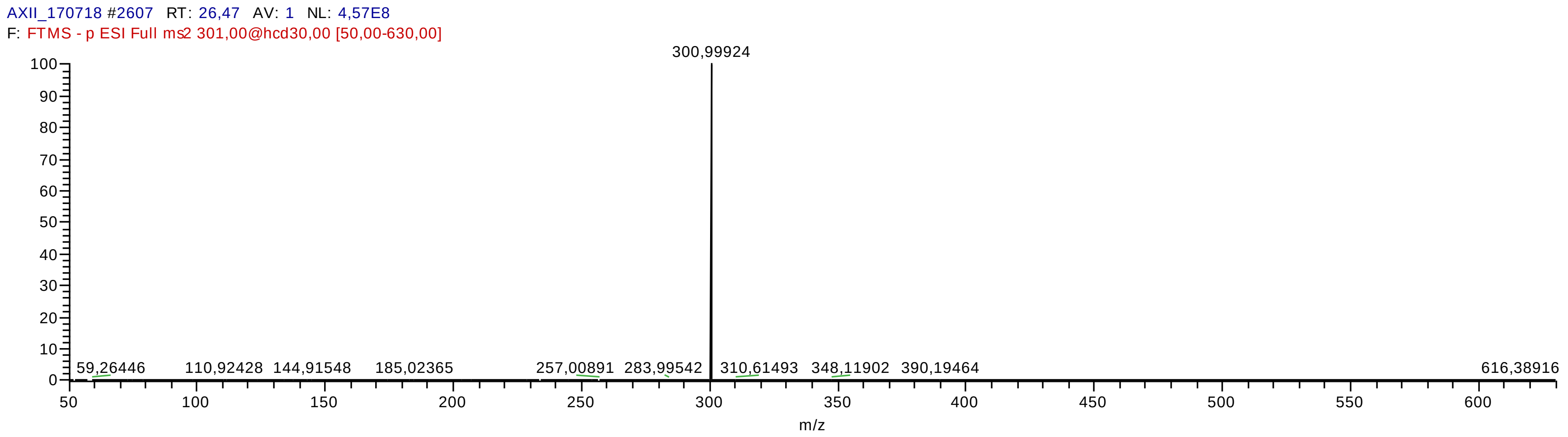

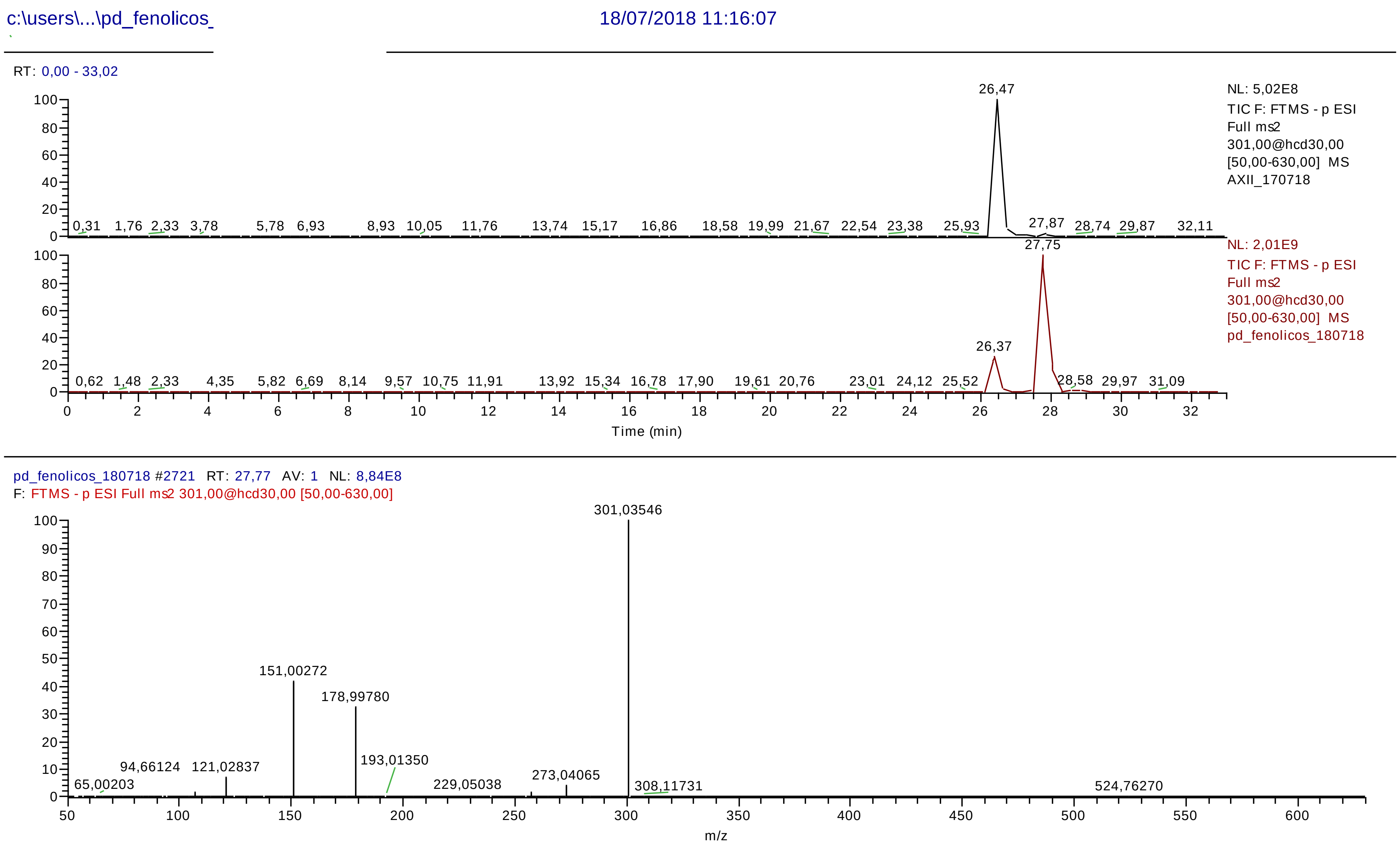
c:\users\...\pd_fenolicos_
18/07/2018 11:16:07
RT: 0,00 - 33,02
NL: 5,02E8
TIC F: FTMS - p ESI Full ms2 301,00@hcd30,00 [50,00-630,00] MS AXII_170718
NL: 2,01E9
TIC F: FTMS - p ESI Full ms2 301,00@hcd30,00 [50,00-630,00] MS pd_fenolicos_180718
Time (min)
pd_fenolicos_180718 #2721 RT: 27,77 AV: 1 NL: 8,84E8
F: FTMS - p ESI Full ms2 301,00@hcd30,00 [50,00-630,00]
301,03546
151,00272
178,99780
193,01350
121,02837
94,66124
65,00203
229,05038
273,04065
308,11731
524,76270
m/z

C:\Users\...\Claudiane\A

17/07/2018 17:38:16

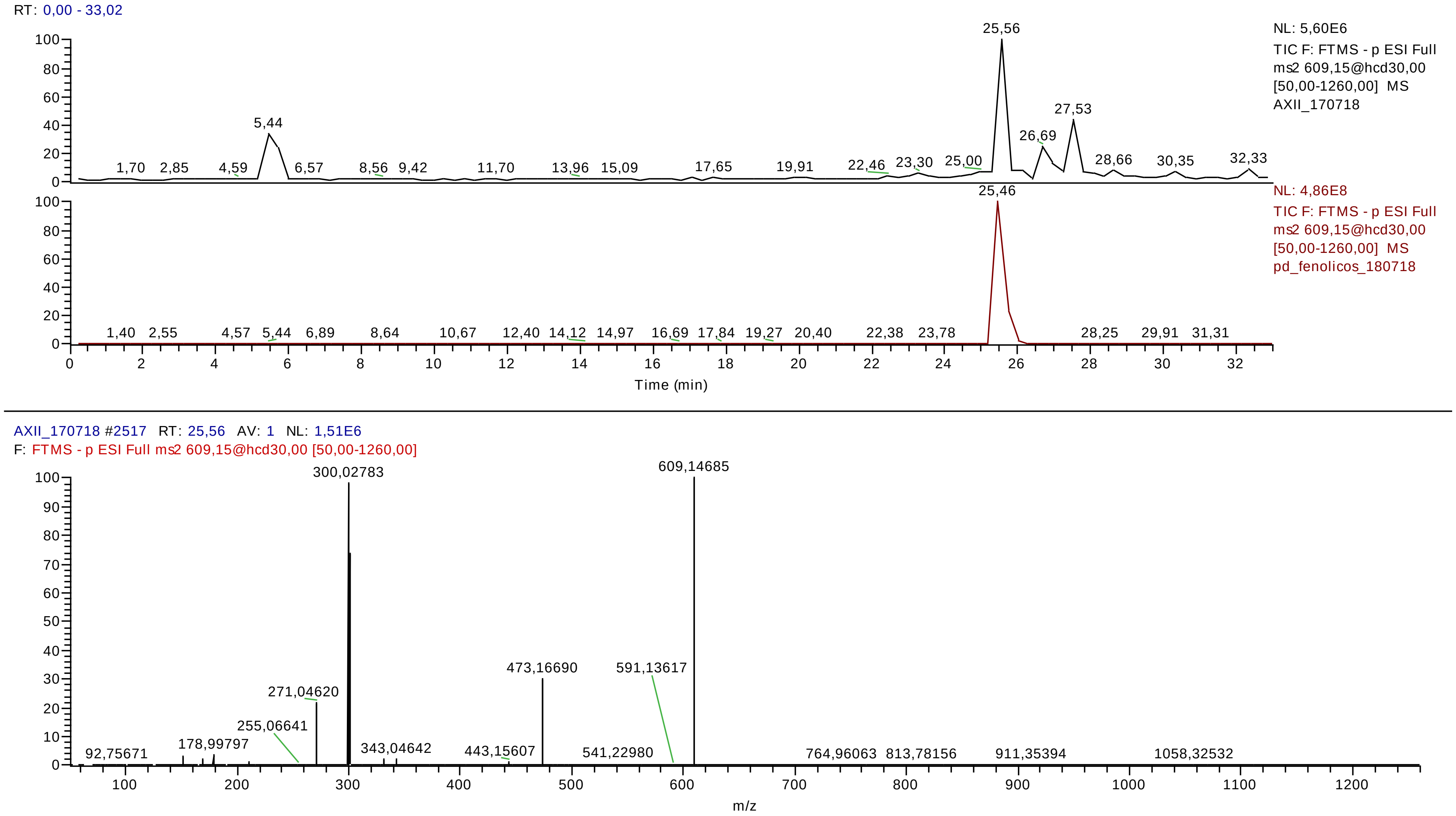

C:\Users\...\Claudiane\A)

17/07/2018 17:38:16

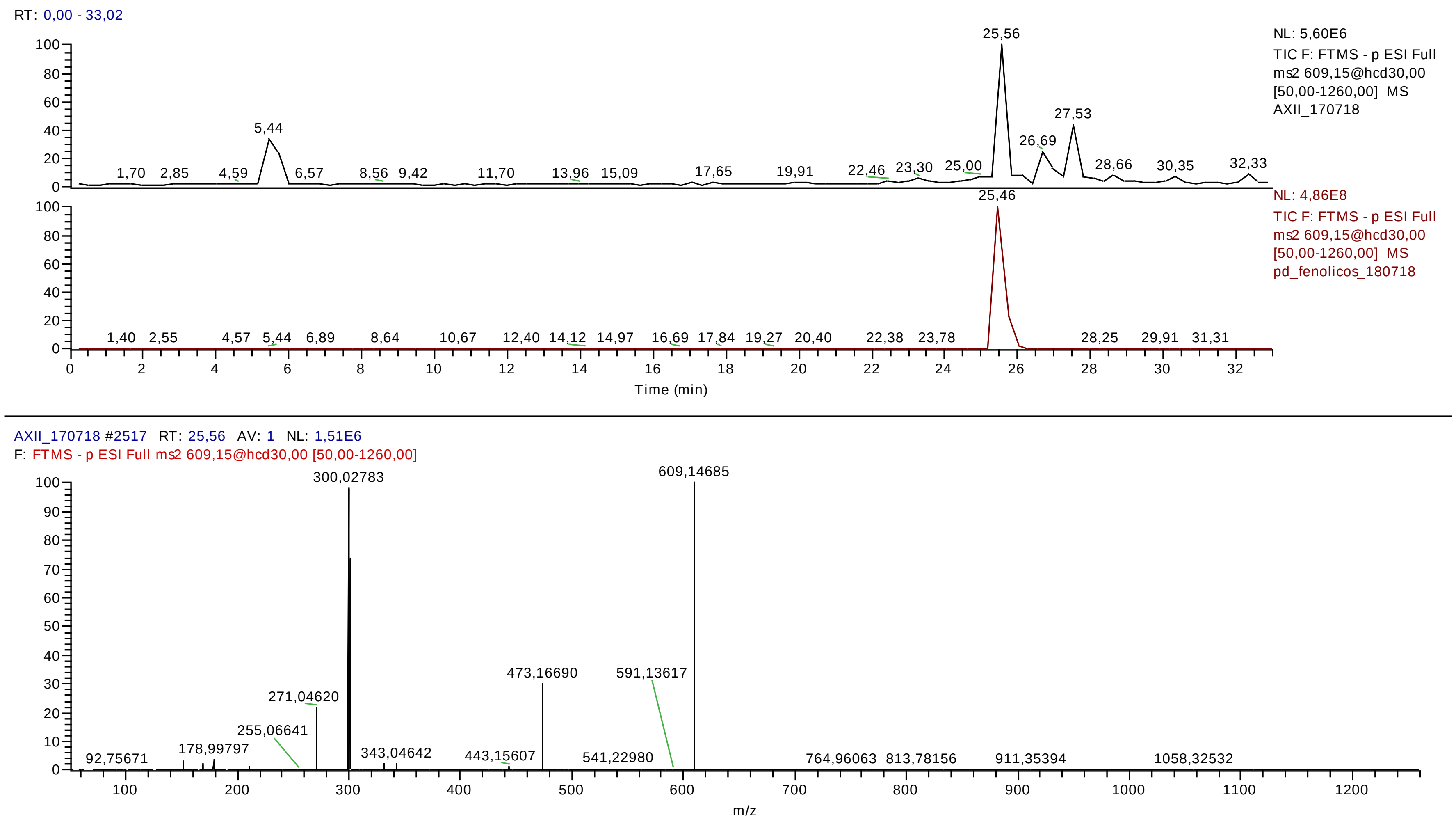

Supplement: Supplementary file 1 [file antioxidants-14-01167-s001.zip › antioxidants-3752896-supplementary.pdf]
